# Fine-tuning the cytotoxicity of ruthenium(ii) arene compounds to enhance selectivity against breast cancers†

**DOI:** 10.1039/d3dt02037a

**Published:** 2023-07-28

**Authors:** Sarah A. P. Pereira, Jan Romano-deGea, Ana Isabel Barbosa, Sofia A. Costa Lima, Paul J. Dyson, M. Lúcia M. F. S. Saraiva

**Affiliations:** a LAQV, REQUIMTE, Departamento de Ciências Químicas, Faculdade de Farmácia, Universidade do Porto Rua Jorge Viterbo Ferreira no 228 4050-313 Porto Portugal lsaraiva@ff.up.pt; b Institut des Sciences et Ingénierie Chimiques, École Polytechnique Fédérale de Lausanne (EPFL) 1015 Lausanne Switzerland paul.dyson@epfl.ch

## Abstract

Ruthenium-based complexes have been suggested as promising anticancer drugs exhibiting reduced general toxicity compared to platinum-based drugs. In particular, Ru(η^6^-arene)(PTA)Cl_2_ (PTA = 1,3,5-triaza-7-phosphaadamantane), or RAPTA, complexes have demonstrated efficacy against breast cancer by suppressing metastasis, tumorigenicity, and inhibiting the replication of the human tumor suppressor gene BRCA1. However, RAPTA compounds have limited cytotoxicity, and therefore comparatively high doses are required. This study explores the activity of a series of RAPTA-like ruthenium(ii) arene compounds against MCF-7 and MDA-MB-231 breast cancer cell lines and [Ru(η^6^-toluene)(PPh_3_)_2_Cl]^+^ was identified as a promising candidate. Notably, [Ru(η^6^-toluene)(PPh_3_)_2_Cl]Cl was found to be remarkably stable and highly cytotoxic, and selective to breast cancer cells. The minor groove of DNA was identified as a relevant target.

## Abstract 2

A ruthenium(ii) arene compound was identified as the most promising candidate for breast cancer treatment amongst a library of rationally designed and fully synthesized compounds where the impact of different counterions’ cytotoxicity was evaluated.

## Graphical abstract

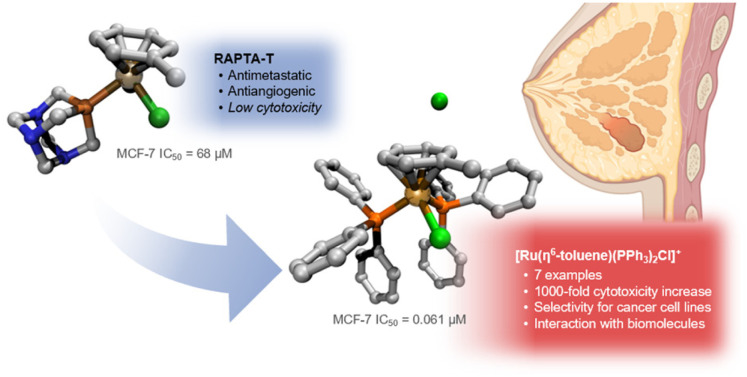

## Introduction

Breast cancer is the most common malignant diagnosed tumor in women and the leading cause of cancer death in the world. Several factors contribute to its high mortality, *e.g.*, acquired resistance during chemotherapy, active invasion, and advanced metastasis.^1,2^ Despite drug resistance, chemotherapy is the main treatment option for breast cancer once it is in an advanced state, and cisplatin is the most frequently used drug used to treat malignant breast cancers, both as a single agent or in combination with other chemotherapeutics.^3–5^ Despite the undoubted success of cisplatin and its derivatives in breast cancer treatment, platinum-based compounds exhibit severe side effects due to their lack of selectivity.^6^

Ruthenium complexes have attracted interest as promising alternatives to platinum-based anticancer drugs, apparently presenting lower side effects, and some show promising activity against different breast cancer cell lines.^7–9^ Although ruthenium-based compounds are not currently used in the clinic, two ruthenium(iii) compounds, NAMI A (ImH[*trans*-RuCl_4_(DMSO-S)(Im)], Im = imidazole)^10^ and KP1019 (IndH[*trans*-RuCl_4_(Ind)_2_], Ind = indazole),^11^ have completed phase I clinical trials. Recently, an analog of KP1019 with a different counterion (Na[*trans*-RuCl_4_(Ind)_2_], known as NKP-1339, IT-139, or BOLD-100) has recently been approved for clinical use as an orphan drug for the treatment of gastric cancers.^12^

Ruthenium(ii)-arene complexes, Ru(η^6^-arene)(PTA)Cl_2_ (PTA = 1,3,5-triaza-7-phosphaadamantane), frequently called RAPTA compounds, and related compounds, have been extensively studied for their anticancer properties.^13^ Although RAPTA compounds tend to display limited cytotoxicity to many cancer cell lines, RAPTA compounds bearing *p*-cymene (RAPTA-C) and toluene (RAPTA-T) have an impact on metastatic tumors *in vivo*,^14,15^ also exerting an antiangiogenic effect^16^ and normalizing blood vessels in the tumor environment.^17^ Subsequently, it has been shown that RAPTA-C reduces the growth of primary tumors in preclinical models for ovarian and colorectal carcinomas.^18^ RAPTA-T also sensitizes cancer cells towards other drugs when combined, and several promising *in vivo* studies against primary tumors were reported.^19–21^

Recently, BRCA1 gene damage in cancer cells has received much attention as a potential molecular target for metal-based anticancer compounds,^22,23^ and RAPTA-T has been shown to inhibit BRCA1 replication in a dose-dependent manner, causing more BRCA1 damage in BRCA1-defective breast cancer (HCC1937 – BRCA1 mutant, triple-negative breast cancer) compared to BRCA1-competent adenocarcinoma (MCF-7) at equal doses.^24^ RAPTA-T exhibits greater effects on highly invasive metastatic MDA-MB-231 breast cancer cells compared to non-invasive MCF-7 cells or non-tumorigenic HBL-100 cells,^25^ selectively inhibiting several steps involved in metastasis progression, *i.e.*, detachment, migration, invasion, and re-adhesion.^15^ Polymeric micelles containing RAPTA-C inhibit the growth of MCF-7 and MD-MB-231 cells grown in both 2D and 3D cultures.^26^ Note that many other ruthenium complexes also display activity against breast cancers *in vivo.*^27–29^

Since RAPTA compounds display promising activity in MCF-7 and MDA-MB-231 breast cancer cells but are not strongly cytotoxic, we hypothesized that more cytotoxic RAPTA-like complexes might offer enhanced activity against breast cancers while potentially retaining selectivity. Hence, we report a study assessing the *in vitro* effects of a series of ruthenium–arene (RAPTA-type or RAPTA-like) compounds on MCF-7 and MDA-MB-231 breast cancer cell lines and non-tumorigenic L929 fibroblasts. For a promising cationic complex, *i.e.* [Ru(η^6^-toluene)(PPh_3_)_2_Cl]^+^, the impact of different counter-anions on the cytotoxicity was explored – an approach rarely explored in inorganic medicinal chemistry beyond changing the counterion to facilitate synthesis, purification, or crystallization.^30–32^

## Results and discussion

### Synthesis and characterization of the compounds

**Fig. 1:**
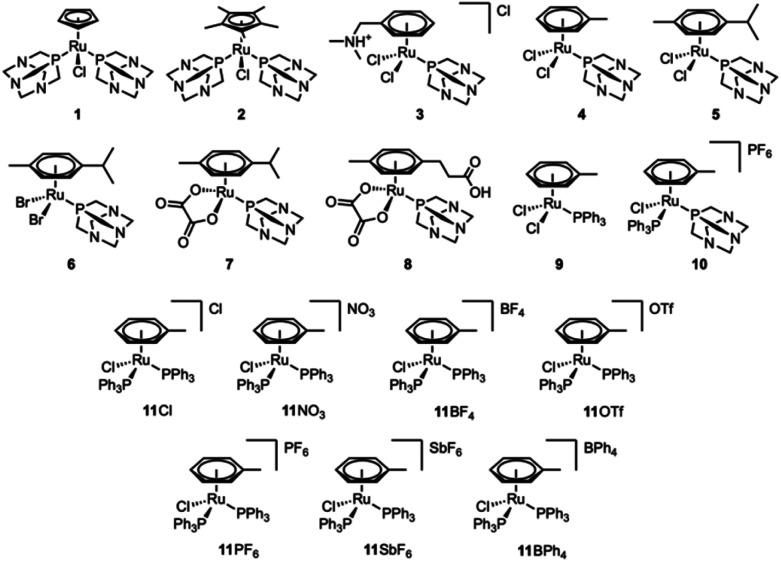
Structures of the compounds used in this study.

A collection of ruthenium(ii)–arene compounds (Fig. 1) was used in the study. Compounds 1,^33^2,^33^3,^34^4,^14^5,^35^6,^35^7,^34^8,^36^ and 9 ^37^ have been reported previously and were prepared according to the literature procedures. Compounds 10, 11NO3, 11BF4, 11OTf, 11PF6, 11SbF6, and 11BPh4 were prepared by the treatment of [Ru(η^6^-toluene)(PPh_3_)Cl_2_] with PTA (10) or triphenylphosphine (11^+^) in the presence of the appropriate silver or sodium counterion salt. Compound 11Cl was prepared by resin anion exchange. Compound 10 is similar to [Ru(η^6^-*p*-cymene)(PTA)(PPh_3_)Cl] reported previously.^38^ Compounds 11BF4 ^39^ and 11PF6 ^37^ have been reported previously, but in this work, they were prepared following a different synthetic route (see Materials and methods).

^31^P NMR spectroscopy confirmed the successful synthesis of 10 and 11CA, which show an upfield shift of the triphenylphosphine singlet in [Ru(η^6^-toluene)(PPh_3_)Cl_2_]. The spectrum of the asymmetrical bis-phosphine complex 10 exhibits two doublets of equal intensity with large ^2^*J*_PP_ couplings of *ca.* 54 Hz, confirming the presence of the two phosphines coupling to each other. Chemical shifts of the coordinated PPh_3_ ligands in 11CA range from 22.1 to 22.8 ppm. No major differences were observed in the chemical shifts of cation 11^+^ in the ^1^H and ^13^C NMR spectra of 11CA. Additionally, complexes 10 and 11CA were analyzed by ESI-MS in positive ion mode, which gave intense peaks with the expected isotopic patterns corresponding to molecular species at *m*/*z* 648 and 753, respectively. Fragmentation of the peak at *m*/*z* 753 corresponding to intact 11^+^ revealed that a phosphine ligand is lost in preference to the arene or the chloride, followed by the loss of the halide and then arene at higher dissociation energies.

**Fig. 2:**
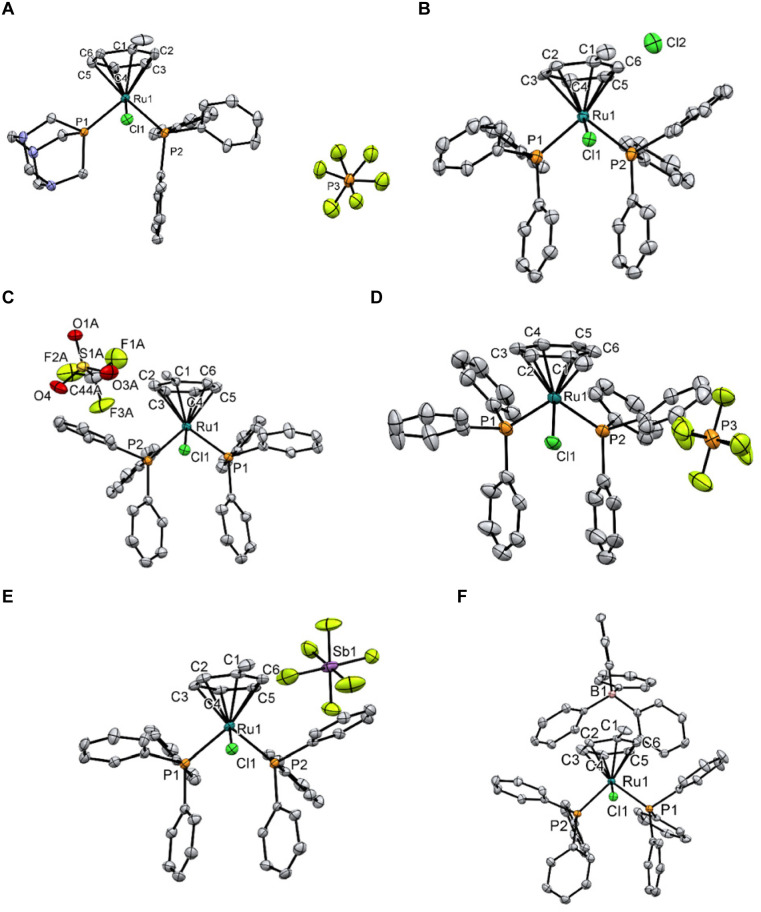
ORTEP diagrams of 10 (A), 11Cl (B), 11OTf (C), 11PF_6_ (D), 11SbF_6 (E)_, and 11BPh_4_ (F). Thermal ellipsoids are drawn with a 50% probability. Solvates and disorders have been omitted for clarity.

Single crystal X-ray structures of 10, 11Cl, 11OTf, 11PF6, 11SbF6, and 11BPh4 were obtained and are shown in Fig. 2 (see ESI† for crystal growth, data collection, and refinement details). All the complexes have the expected piano-stool-type conformation with the methyl group of the coordinated arene locked in the same plane as the Ru–Cl bond. No significant structural differences were observed in the selected bond lengths and angles of the solid-state structures of the cation in 11CA (CA – counter-anion) (Table S1†), highlighting the absence of an effect in the crystal due to the different counterions.

Compounds containing labile metal–halide bonds, such as cisplatin or RAPTA-C, undergo hydrolysis and/or ligand substitution as a key activation hallmark of their therapeutical activity.^40^ Among other methods, NMR spectroscopy and mass spectrometry are widely employed to study these reactions and elucidate the fate of a compound in a physiological environment. The stability of 11CA under pseudo-physiological conditions was evaluated by ESI-MS with speciation and the relative intensity of the different ruthenium-containing ions recorded over 72 h. All complexes remained stable under the conditions (Fig. S2–S8†), and no noticeable differences were observed as a consequence of the different counterions. As 11Cl displays higher solubility than the other salts in aqueous media (Table S3†), its stability was additionally studied by NMR spectroscopy in neutral conditions. No signs of hydrolysis or decomposition were observed (Fig. S9 and S10†). To further confirm the inertness of the Ru–Cl bond, 11Cl and 11BPh4 were incubated for 72 h in DMSO, a strongly coordinating solvent. No changes were observed, confirming the overall resistance of 11CA to solvolysis (Fig. S11–S14†). The inertness of 11CA is likely due to the difficulty of eliminating a negatively charged Cl^−^ ligand from a cationic complex to afford a doubly charged species. For example, note that the second hydrolysis of RAPTA-C is only facilitated after the deprotonation of first-coordinated water.^40^

### *In vitro* studies

The cytotoxicity of 1–11CA was assessed against human breast cancer MCF-7 and MDA-MB-231 cells, and non-tumorigenic adherent mouse fibroblast connective tissue L929 cells using the MTT assay after 24 and 72 h (Tables S5† and Table 1, respectively). The dose–response curves of compounds 10 and 11CA at 24 h and 72 h are presented in Fig. S15.†

**Table 1:** Cytotoxicity of 1–11CA against breast cancer (MCF-7 and MDA-MB-231), and non-tumorigenic adherent mouse fibroblast connective tissue (L929) cells after 72 h of exposure. See Table S6 in the ESI† for the full statistical analysis

Compound	IC_50_ (μM) (average ± standard deviation) 72 h			SI^a^
	MCF-7	MDA-MB-231	L929	
1	48 ± 10	41 ± 3	58 ± 4	1.3
2	51 ± 6	63 ± 3	64 ± 4	1.1
3	51 ± 4	60 ± 3	84 ± 5	1.5
4	68 ± 7	83 ± 4	79 ± 4	1.0
5	57 ± 5	71 ± 4	84 ± 4	1.3
6	74 ± 7	63 ± 5	82 ± 3	1.2
7	62 ± 4	83 ± 5	104 ± 4	1.4
8	41 ± 5	50 ± 2	50 ± 3	1.1
9	52 ± 7	44 ± 3	70 ± 4	1.5
10	3.1 ± 0.2	2.8 ± 0.2	31 ± 3	10.5
11Cl	0.061 ± 0.003	0.086 ± 0.006	0.87 ± 0.05	11.8
11NO_3_	0.078 ± 0.005	0.109 ± 0.009	1.04 ± 0.05	11.1
11BF_4_	0.14 ± 0.01	0.19 ± 0.01	1.13 ± 0.05	6.8
11OTf	0.60 ± 0.07	1.4 ± 0.1	1.8 ± 0.2	1.8
11PF_6_	0.15 ± 0.01	0.16 ± 0.01	0.17 ± 0.01	1.1
11SbF_6_	0.08 ± 0.01	0.131 ± 0.009	1.35 ± 0.09	12.8
11BPh4	0.52 ± 0.06	1.7 ± 0.3	3.9 ± 0.3	3.5

a The selectivity index (SI) was calculated as IC_50_ non-cancerous L929 cells divided by the average of the IC_50_ of the breast cancer cell lines.

There is a direct correlation between cell growth inhibition and the lipophilicity of the complexes (Table S3†). Compounds 1–9 exhibit IC_50_ values >40 μM and low selectivity indexes. The presence of hydrophobic triphenylphosphine (PPh_3_) ligands increases the cytotoxicity of the complexes. Compound 10, bearing a hydrophilic PTA and a hydrophobic PPh_3_ ligand, is considerably more cytotoxic than 1–9 and shows good cancer cell selectivity. Compounds 11CA with two PPh_3_ ligands even reach cytotoxicity values in the nanomolar range with some having a selectivity index (between non-tumoral and breast cancer cell lines) above 10. Note that since the sodium salt of BPh_4_^−^ presented the lowest IC_50_ values in all the cell lines at both incubation times (see Table S7 in the ESI†), it might be expected that 11BPh_4_ would be the most cytotoxic in the series, but that was not the case. Indeed, 11BPh_4_ exhibits the lowest cytotoxicity among all the 11CA compounds (*cf*. IC_50_ of 11BPh_4_ and 11Cl in MCF-7 cells 0.52 ± 0.06 μM and 0.061 ± 0.003 μM, respectively), highlighting that the activity of these salts is not correlated with the intrinsic toxicity of the counterions. Possibly, the toxicity is much more affected by the solvation and dissociation of the complex in aqueous media and the availability of the complex to interact within the cell environment as highlighted by the conductivity measurements and calculated hydration energies (Tables S3 and S4†).

Compounds 11CA are more active against breast cancer cell lines than platinum-based chemotherapeutics currently used in the clinic (cisplatin, 28 ± 6 μM (ref. 41) and 38.70 ± 0.03 μM;^42^ carboplatin, 27.3 μM (ref. 43) and 15.4 ± 3.3 μM (ref. 44) for MCF-7 and MDA-MB-231 cells, respectively). Compared to previously reported complexes tested against breast cancers, the most active representatives of the 11CA series (11Cl, 11NO_3_, and 11SbF_6_) are comparable to the most cytotoxic ruthenium-based^45^ or metal drugs^46^ reported for breast cancers. Furthermore, 11CA are more cytotoxic than RAPTA-EA, a selective glutathione transferase (GSTP-1) inhibitor, which shows good cytotoxicity profiles against breast cancer cell lines (20.0 ± 2.2 μM and 10.5 ± 0.5 μM for MCF-7 and MDA-MB-231 cells, respectively).^47^

**Fig. 3:**
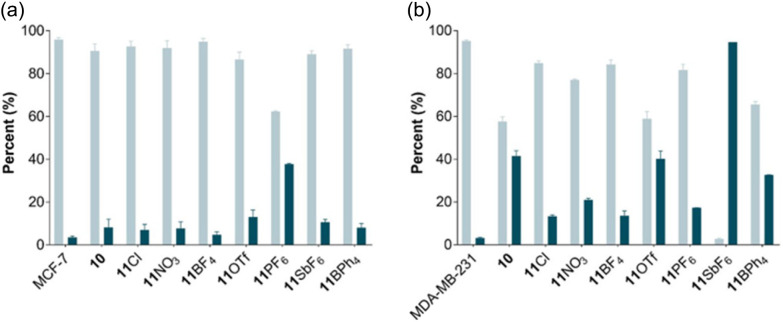
Induction of apoptosis by 10 and 11CA on breast cancer cell lines. Determination of live and apoptotic events treated with 10 and 11CA for 24 h. (a), MCF-7 and (b), MDA-MB-231; 
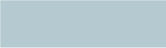
 viable and 
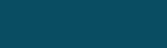
 apoptotic events. The flow cytometry graphs for the tested compounds are provided in Fig. S16 and the IC_50_ values at 24 h are provided in Table S5 of the ESI.†

The most active and selective compounds identified from the cytotoxicity studies are 10 and 11CA and were therefore tested for their ability to induce cell death *via* apoptosis, the preferred mechanism of cell death^48^ and sometimes associated with multidrug-resistance.^49^ The compounds were incubated with MCF-7 and MDA-MB-231 cells at their 24 h IC_50_ dose and Annexin V caspase assay was used to determine the extent of apoptosis (Fig. 3).

With the exception of 11PF_6_, the tested compounds induce a slight increase in the number of apoptotic MCF-7 cells to approximately 10% compared to the control. Induction of apoptosis was more pronounced in the MDA-MB-231 cells, especially for 10, 11OTf, 11SbF_6_, and 11BPh_4_, presenting average apoptosis percentages of 41, 40, 95, and 33%, respectively. Negligible necrosis (<2%) was observed for all the compounds (Fig. S3†). Ruthenium compounds inducing >60% of apoptotic cells are rare, but not unreported. For example, cationic ruthenium complexes containing heterocyclic thioamidates induce nearly 75% of cell apoptosis at 24 h and 82% at 48 h in a hepatocarcinoma cell line.^50^ Some cationic chloroquine-functionalized ruthenium complexes also induce apoptosis in *ca.* 90% after 16 h of incubation in acute T-cells leukemia Jurkat cells.^51^ Conversely, the high apoptotic cell values of 11SbF_6_ could be related to the antimony counterion as other antimony-containing compounds, including sodium stibogluconate (an antileishmanial drug), SbCl_3_, or Sb_2_O_3_, induce significant apoptosis in acute promyelocytic leukemia cell lines.^52,53^ Hence, the antiproliferative activity of 11SbF_6_ (Table 1) might be partially linked to apoptosis induction of the antimony counterion. However, the induction of apoptosis of the remaining complexes does not necessarily correlate with the higher cytotoxicity or the selectivity of the complexes (Table 1), indicating that other cell death mechanisms may be involved.^54,55^ Compounds that are able to induce programmed cell death *via* alternative mechanisms might be able to overcome apoptosis-related resistance, suggesting that 11Cl and 11BF_4_ could have potential applications in cancers that develop apoptosis resistance.^56^

### Interaction of 11CA with biomolecules

Nucleophilic model biomolecules were initially employed to explore the reactivity between 11Cl and potential biological targets. Compound 11Cl was incubated with three nucleophilic amino acids, *i.e.*, glutamic acid (Glu), histidine (His), and sulfur-bearing cysteine (Cys), and the nucleotide deoxyguanosine monophosphate (dGMP) and the reactions were monitored by NMR spectroscopy and mass spectrometry. No changes in the model biomolecules, the metal complex, or adduct formation were observed during the incubation of 11Cl with any of the model compounds (Fig. S17–S28†), suggesting that the 11^+^ cation does not exert its anticancer effect *via* covalent binding. To exclude the possibility of a catalytic mode of action, common to other ruthenium complexes,^57–59^11Cl was evaluated as a catalyst for the oxidation of GSH and in the transfer of hydrogenation of NAD^+^ to 1,4-NADH. No catalytic or stoichiometric conversion was observed, and the compound remained unchanged (Fig. S29–S32†).

These results indicate that 11Cl might engage in non-covalent interactions with biomolecules, similar to other inert ruthenium complexes.^60–62^ Hence, the interaction between 11Cl and bovine serum albumin (BSA), employed as a model plasma protein, was evaluated by measuring the fluorescence quenching of tryptophan. The calculated value for the bimolecular quenching rate due to short-range interactions, *k*_q_, is 1.9 × 10^12^ M^−1^ s^−1^. This value is higher than the average diffusion-controlled quenching rates of representative biomolecules in aqueous media (2 × 10^10^ M^−1^ s^−1^),^63^ indicating that fluorescence quenching is due to specific interactions between 11Cl and the protein. The calculated value for the bimolecular quenching rate due to short-range interactions, *k*_q_, indicates that the decrease in fluorescence is a result of specific interactions between 11Cl and the protein. The binding constant of 11Cl to BSA (5.6 × 10^5^ M^−1^) is moderate compared to the reported binding constants of other ruthenium(ii) arene complexes.^64,65^ Furthermore, the estimated value of *n* (∼1) supports the likelihood of an unimolecular single binding site for 11Cl in BSA (Table S8 in ESI†).

To gain insights into potential interactions between 11Cl and DNA, a fluorescence-based competitive binding assay was conducted using three DNA probes (Table 2), *i.e.*, DAPI (minor groove binder), MG (major groove binder), and PI (intercalator). From the obtained fluorescence data, apparent DNA binding constants, *K*_app_, were calculated according to the competitive binding model described by Tse and Boger.^66^ A decrease in the emission intensity of DAPI and MG was observed as the concentration of 11Cl increased, without their respective emission wavelengths changing. In contrast, no linear quenching was observed with PI. This data suggests that 11Cl does not intercalate DNA but interacts with the major or minor groove of DNA *via* non-covalent interactions. The difference between the obtained apparent binding constants, *K*_app_, (4.4 × 10^5^ M^−1^ and 0.43 × 10^5^ M^−1^, calculated from the competitive binding of MG and DAPI, respectively) suggests that 11Cl shows a 10-fold preference towards the minor groove (Table S9 in ESI†). The calculated binding constants are in line with those reported for other DNA-binding ruthenium(ii)–arene complexes^65,67^ and for ruthenium(ii) groove-binding compounds.^68,69^

**Table 2:** Summary of the docking results between 11^+^ and the different DNA structures, including the number of minor (minG) and major (MajG) grooves and other conformations and the lowest (and average) binding energy of the different conformations

PDB structure	*N*_minG_ (%)	Δ*G*_minG_ (kcal mol^−1^)	*N*_MajG_ (%)	Δ*G*_MajG_ (kcal mol^−1^)	Other	*N*_other_ (%)	Δ*G*_other_ (kcal mol^−1^)
1BNA (*native*)	59	−5.62 (−4.65)	41	−5.38 (−4.77)	0	—	—
5T4W (*minor groove*)	79	−6.41 (−5.39)	21	−5.68 (−5.12)	0	—	—
1G3X (*intercalator*)	23	−8.89 (−8.23)	0	—	Int. + minG	76	−8.95 (−8.63)
					Int. + MajG	1	−6.42

**Fig. 4:**
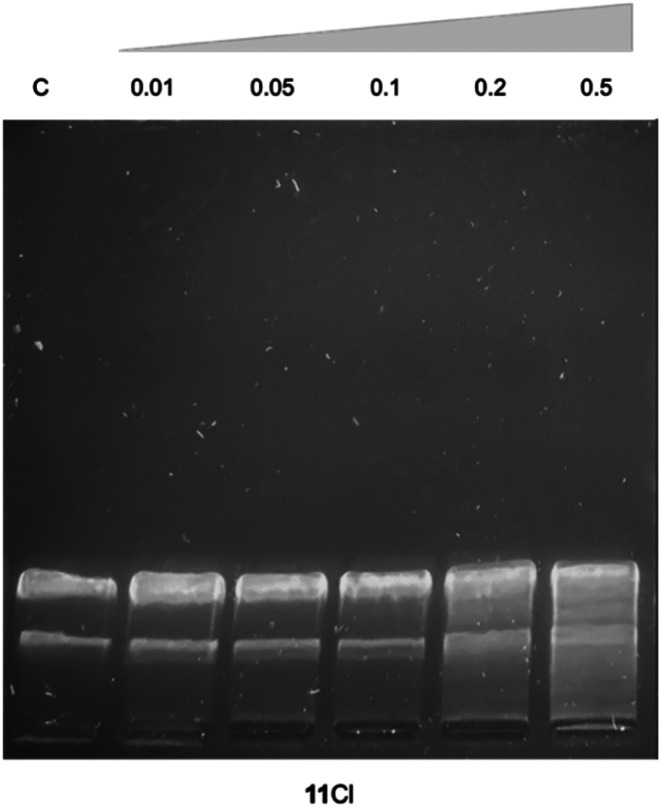
Gel electrophoresis of 11Cl with pBR322 DNA. C – control; 11Cl at *r*_i_ 0.01; 0.05; 0.1; 0.2 and 0.5 (*r*_i_ = [11Cl]/[DNA bp]).

The interaction of 11Cl with plasmid DNA was also assessed by visualizing the relaxation of the plasmid using agarose gel electrophoresis (Fig. 4). Different concentrations of the 11Cl were incubated with pBR322 DNA at 37 °C for 24 h and then analyzed by gel electrophoresis (Fig. 4). Compound 11Cl did not induce the stabilization of the linear form, further corroborating that 11Cl does not interact covalently with DNA.^70^ However, at ratios from 0.2, 11Cl produces alterations in the supercoiled structure of the plasmid in the form of tailing and a slight retardation on the bands, which might be an indication of non-covalent interactions with the DNA.^71^ Notably, no precipitation was observed in any of the studied concentrations, indicating that the 11Cl charge does not neutralize DNA and, therefore, implying that strong electrostatic binding and aggregation of DNA by 11CA as the mechanism of action is unlikely.^72,73^

**Fig. 5:**
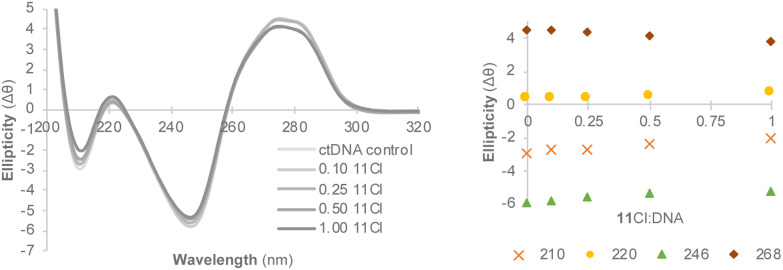
Circular dichroism spectra of 11Cl (left) and changes in the intensity of the main bands (right).

Circular dichroism (CD) was used to identify the non-covalent binding mode of 11Cl.^74^ The CD profile of right-handed B-DNA (its most common form) displays two positive (220 and 268 nm) and two negative (210 and 246 nm) elliptical signals.^75^ Compound 11Cl induced changes in the intensity of the bands of ctDNA (Fig. 5), characterized by a decrease in the ellipticity of the bands at 210, 246 and 270 nm, and an increase in the circularity of the band at 220 nm. A small bathochromic (red) shift was also observed for the band at 220 nm, whereas the maximum wavelength remained unchanged for the other the bands. This data indicates that 11Cl induces changes in the circularity of DNA and distorts its secondary structure.

The CD spectra of 11Cl with B-DNA were compared to the spectra of the probes DAPI (minG), MG (MajG) and PI (Int.) (Fig. S33 and Table S10 in the ESI†) indicate that 11Cl interacts with both the minor and major grooves of DNA. This finding is further supported by the induced circular dichroism (ICD) spectra of the DNA probes before and after the addition of 11Cl (Fig. S34†). The intensity of the ICD bands of DAPI and MG decreased by 35% and 20%, respectively, following treatment with 11Cl. Moreover, the difference in quenching between these probes confirms that 11Cl prefers to interact with the minor groove. Additionally, the induced changes in the CD spectra were not coherent with those observed for previously reported DNA condensation agents,^76^ further rejecting DNA aggregation as a mode of action.

Tandem mass spectrometry was used to investigate the binding mode interaction of 11Cl with single- (ss) and double-strand (ds) DNA oligomers.^77,78^ After incubation, 43 and 23% of the identified ssDNA and dsDNA ions contained ruthenium, respectively, with [Ru(η^6^-toluene)(PPh_3_)]^2+^ as the main ruthenium adduct in the gas phase (in agreement with the fragmentation behavior observed during the characterization of the complexes, see above). The interaction between the oligonucleotides and 11Cl is non-covalent in nature (in agreement with fluorescence and circular dichroism data) as loss of the ruthenium from the isolated adduct can be observed even when no collision-induced dissociation energy (to fragment the ions) is applied. Fragmentation of 14-mer ssDNA ions containing [Ru(η^6^-toluene)(PPh_3_)]^2+^ resulted in longer nucleotide fragments compared to the intact oligomer (12 ± 2 *vs.* 6 ± 4 nucleotides, Fig. S35 and S36†), arising from the stabilization of the ruthenium-bound DNA,^79^ likely through the interaction of the cationic 11^+^ with negatively-charged phosphate groups.^80^ Likewise, fragmentation of 6-mer dsDNA ruthenium-bound ions led to a similar outcome (5 ± 1 *vs.* 3 ± 1 nucleotides, Fig. S35 and S37†). In both studied oligonucleotides, w-type fragmentation, and base loss remained the main fragmentation mechanisms in intact DNA (native) and metal-free (fragments originating from the isolated ruthenium-DNA adduct but not containing the metal) ions.^81,82^ However, ruthenium-containing ions displayed a decrease in the number of fragments (more unfragmented ions were present) and in the number of internal fragments, as well as enhancing base-loss fragmentation pathways (Fig. S38 and S39†). This behavior indicates that 11Cl influences the fragmentation behavior of the oligonucleotides, supporting the stabilization of DNA by the ruthenium complex through an interaction with its secondary structure. Additionally, the lack of an apparent nucleotide selectivity or sequence binding preference,^83,84^ and the promotion of base-loss pathways,^85^ while retaining the backbone and sugar fragments, are coherent with the absence of covalent interactions between the nucleobases and 11Cl, further confirming its interaction with the phosphate backbone in the DNA grooves.

**Fig. 6:**
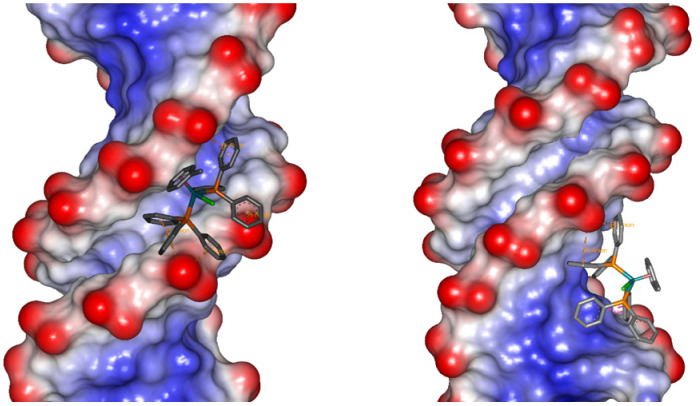
Docked conformations of 11^+^ with the minor (right) and major (left) grooves of 1BNA.

Molecular docking studies were performed to probe the nature of the interactions of 11^+^ with the major and minor grooves of DNA. Since 11^+^ is chemically inert, standard biomolecule-ligand docking methodologies were used as the metal mainly plays a structural role and does not require a precise electronic description.^86^ The molecular docking studies support the experimental results, confirming that both the major and the minor grooves in B-DNA are likely binding sites for 11^+^ (Fig. 6. The binding of the minor groove is slightly favored, supported by the larger number of poses and lower binding energies (Table 2). This preference is also observed in other B-DNA structures (Fig. S40†). The nature of the interaction of 11^+^ with DNA depends principally on the hydrophobic interactions and electrostatic attraction between the ruthenium cation and the negatively charged DNA phosphate backbone (Fig. S41†). The electrostatic component only represents an average of 15% or 8% of the total binding energy, for the minor groove and the major groove poses respectively. To a lesser extent, these conformations are also favored by π-interactions between the aromatic rings on 11^+^ and the nucleobases.

The performed studies have helped elucidate the possible biological targets of 11CA. The hydrolysis-resistant complexes interact with biomolecules in non-covalent fashion, deviating from the more typical mechanisms associated with metallic drugs. 11Cl binds both proteins and nucleotides and, as expected from the highly lipophilic character of the compound, it displays a preference for binding the minor groove of DNA. It can be expected that these interactions are fundamental for the high antiproliferative activity displayed by the developed cationic complexes.

## Conclusions

From a collection of ruthenium(ii)–arene compounds a hydrophobic, cationic complex, 11Cl, was identified as a promising candidate for the treatment of breast cancers, presenting inhibitory growth rates in the nanomolar range, and displaying around a 10-fold selectivity for the breast cancer cell lines compared to the healthy ones. The interaction studies confirmed that 11Cl interacts with biomolecules in a non-covalent fashion with a preference for binding the minor groove of DNA.

## Experimental

[Ru(η^6^-toluene)Cl_2_]_2_,^87^ [Ru(η^6^-toluene)(PPh_3_)Cl_2_]^37^ and 1,^33^2,^33^3,^34^4,^14^5,^35^6,^35^7,^34^8 ^88^ and 9 ^37^ were prepared according to reported procedures and their spectroscopic data are in agreement with those reported. Compounds 10 and 11CA (CA = counteranion, 7 examples) were prepared by the treatment of [Ru(η^6^-toluene)(PPh_3_)Cl_2_] with PTA or triphenylphosphine in the presence of the appropriate silver or sodium counterion salt, except in the case of 11Cl which was prepared by resin anion exchange. Compounds 11BF_4_ ^39^ and 11PF_6_ ^37^ were previously reported, but in this work, they were prepared following a different synthetic route. Full experimental details and characterization data are provided in the ESI (Fig. S1 and Tables S1 and S2).†

Adherent mouse fibroblast connective tissue cells (L929) and human breast cancer cells (MCF-7 and MDA-MB-231) were obtained from the European Collection of Authenticated Cell Cultures (ECACC), Public Health England, Salisbury, UK and ATCC, Middlesex, UK, respectively. All cell culture reagents *i.e.*, Dulbecco's modified Eagle's medium (DMEM), fetal bovine serum (FBS), penicillin–streptomycin mixture, and trypsin-EDTA 0.25% (v/v) were purchased from Gibco® (Invitrogen Corporation, UK). Deoxyribonucleic acid (calf thymus) and Dimethyl sulfoxide (DMSO) were purchased from Merck® (EMD Millipore Corp., USA). FITC Annexin V Apoptosis Detection Kit with 7-AAD was purchased from BioLegend®. Agarose basic was purchased from PanReac AppliChem®. pBR322 DNA, GeneRuler 1 kb Plus DNA ladder, and SYBR™ Safe DNA Gel Stain were purchased from Thermo Fisher Scientific®. Methyl green, DAPI, and propidium iodide 95% were purchased from Sigma-Aldrich®, Ivalua®, and Acros Organics®, respectively.

### Stability in solution and reactivity with model biomolecules

The stability of 11CA under pseudo-physiological conditions was assessed using ESI-MS and ^1^H and ^31^P NMR spectroscopy. Furthermore, the interaction between 11Cl and different model biomolecules (amino acids: glutamic acid, histidine and cysteine, and nucleotide, dGMP), was also studied. The catalytic activity of 11Cl in transfer hydrogenation and glutathione (GSH) oxidation reactions was monitored by ^1^H and ^31^P NMR spectroscopy under previously reported biologically relevant conditions.^57^

### Preparation of stock solutions of the compounds

Stock solutions were prepared in supplemented DMEM with 10% DMSO to ensure solubility. Then, necessary dilutions were made using the supplement DMEM, to prepare the range of tested concentrations. Since DMSO was used to solubilize the compounds, a supplemented DMEM + DMSO (at the same percentage as in the sequential dilutions) was considered as a control in the 3-[4,5-dimetylthiazole-2-yl]-2,5-diphenyltetrazolium bromide (MTT) assay.

### Cell lines and culture conditions

L929, MCF-7, and MDA-MB-231 cell lines were cultured in DMEM supplemented with 10% (v/v) FBS and 1% (v/v) of penicillin–streptomycin. The cells were incubated in a humidified chamber at 37 °C under saturated air with a 5% CO_2_ atmosphere. Cells were passed at 80%–90% of confluency with 0.25% (w/v) trypsin and the cell culture media was changed every 2–3 days.

### Cell viability assessment

The compounds were tested on two breast cancer cell lines (MCF-7 and MDA-MB-231) and L929 fibroblasts, used as a reference for the safety evaluation according to ISO 10993-1:2009 “Biological evaluation of medical devices”. Cells were seeded on sterile 96-well plates at 50 000 cells per well (L929) and 20 000 cells per well (MCF-7 and MDA-MB-231) in supplemented DMEM medium (100 μL) for 24 h. The culture medium was removed and new media containing different concentrations of the compounds (0–1000 μM) was added to the wells (100 μL). Control cells were incubated under the same conditions in the absence of a compound. Cells were incubated for 24 and 72 h under the same conditions described above. Then, the medium was removed, and the cells were incubated with 3-(4,5-dimethylthiazol-2-yl)-2,5-diphenyltetrazolium bromide (MTT; 0.5 mg mL^−1^) for 3 h. After this time, the medium was removed, and formazan crystals were solubilized with DMSO (100 μL per well). The absorbance in each well was read on a SynergyTM HT Multimode microplate reader (BioTek Instruments; Winooski, VT, USA) at 570 and 630 nm (for background correction), and the cell viability was calculated according to the following equation (eqn (1)):1
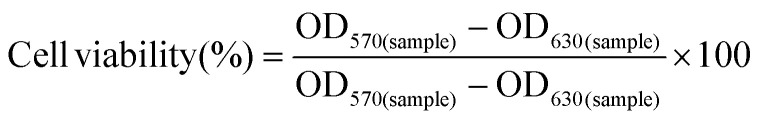
where OD_570(sample)_ and OD_630(sample)_ are the optical densities of the solutions containing the compounds and OD_570(control)_ and OD_630(control)_ are controls without compound.

### Analysis of cell apoptosis

Apoptosis in MCF-7 and MDA-MB-231 cells was determined using FITC Annexin V with a 7-aminoactinomycin D (7-AAD) staining kit (BioLegend®, California, US) according to the manufacturer's instructions. Briefly, cells (120 000 cells per well) were incubated in sterile 24-well plates with the IC_50_ concentration of the compounds for 24 h. Following incubation, the cells were washed with PBS twice and detached using 0.25% trypsin. After obtaining cell pellets by centrifugation (300*g* for 5 min), the cells were resuspended in 100 μL of PBS, followed by the addition of 10 μL of FITC Annexin V and 7-AAD, and then incubated for 15 min at room temperature while protected from light. After the incubation period, 200 μL of binding buffer was added to the cell suspension and the samples were analyzed immediately using a BD Accuri C6 flow cytometer with a minimum of 10 000 events. The assays were performed in quadruplicate and the results were analyzed with Accuri C6 analysis software.

### BSA binding assay

BSA (16 μM) in 10 mM Tris-HCl pH 7.6 + 1 mM EDTA solution and varying concentrations of 11Cl (0–32 μM) were incubated at 37 °C for 24 h. Afterward, the fluorescence spectra were measured for each solution between 300 and 500 nm (*λ*_ex_ = 280 nm). The intensity at *λ*_max_ (340 nm) was recorded. Quenching parameters were obtained by applying the Stern–Volmer equation (eqn (2)):2
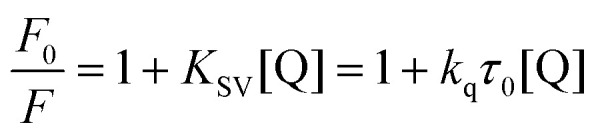
where *F*_0_ and *F* represent the fluorescence intensities of the DNA–dye complex in the absence and presence of 11Cl, respectively; *K*_SV_ is the linear Stern–Volmer quenching constant; [Q] is the molar concentration of the quencher, *i.e.*, metal complex; *k*_q_ is the bimolecular quenching rate constant; and *τ*_0_ is the lifetime of BSA fluorescence in the absence of any quencher (6.0 ns.^89^ The modified Stern–Volmer plot (eqn (3)) was used to obtain the binding parameters:3
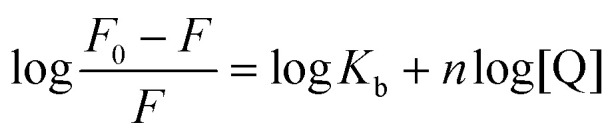
where, *F*_0_ and *F* are the fluorescence intensities of BSA before and after metal complex binding; *K*_b_ is the binding constant; *n* is the number of binding sites, and [Q] is the molar concentration of the quencher.

### Competitive DNA binding assay

Emission intensity measurements were performed using a SpectraMax M5e reader at room temperature. A 50 μM (bp) of ct-DNA was pre-incubated with the groove binding agents (MG and DAPI) (2 μM) and the intercalating agent propidium iodide (2 μM) for 30 min at 37 °C, to allow full interaction of the dyes into the ctDNA. Increasing amounts of 11Cl (0–100 μM) were added to this mixture and the final samples were incubated for 24 h at 37 °C. After incubation, the emission spectra were recorded at excitations of 633, 358, and 535 nm for the measurements of MG, DAPI, and PI, respectively. Quenching parameters were obtained by applying the Stern–Volmer equation (eqn (2)). The apparent binding constants, *K*_app_, were calculated according to the competitive binding model described by Tse and Boger^66^ using eqn (4):^90^

4 *K*_probe_[probe] = *K*_app_*c*_50_

where *K*_probe_ is the binding constant of the probe, 9 × 10^5^ and 5 × 10^6^ M^−1^ for MG^91^ and DAPI,^92^ respectively; [probe] is the concentration of probe used, 10 μM for both MG and DAPI; and *c*_50_ is the complex concentration when the fluorescence intensity of the probe is 50%. This value is obtained from the plot *F*_0_/*F vs.* [Q] when *F*_0_/*F* = 2.

### Interaction of 11Cl with plasmid DNA by gel electrophoresis

Assays were performed using 1.2% (w/v) agarose gels. A plasmid pBR322 DNA was purchased from Thermofisher Scientific® at 0.5 μg μL^−1^ concentration in phosphate buffer 50 mM (pH 7.4). A 20 μL portion containing 0.125 μg μL^−1^ of DNA in 10 mM Tris-HCl (pH 7.6) and 1 mM EDTA was incubated with increasing volumes of the stock solution of 11Cl (1 mM in DMSO) at *r*_i_ values ranging from 0.05 to 0.5 (*r*_i_ = [complex]/[no. nucleotide]). Samples were incubated at 37 °C for 24 h, after which 2 μL of the TriTrack DNA loading dye buffer was added. 20 μL of the sample was loaded in the agarose gel (1.2% w/v), and electrophoresis was carried out for a period of 100 min at 40 V in TAE 1× (Tris-acetate/EDTA) buffer. After electrophoresis, the gel was immersed in 200 mL of TAE 1× buffer containing 20 μL of a stock solution of SYBR safe for 30 min to stain the DNA. Then, the gel was washed with Mili Q water for 10 min and the stained gel was analyzed with a blue light in a Safe Imager™ 2.0 (Invitrogen).

### Interaction of 11Cl with calf thymus DNA by circular dichroism

Calf thymus (ct) DNA (Merck®) was used to perform the circular dichroism assay to detect conformational changes in the ctDNA induced by the compounds. CD spectral data was collected on a Chirascan™ V100 (Applied Photophysics) spectropolarimeter equipped with a large area Avalanche photodiodes detector and computer-controlled thermoelectrical cell holder using a 1 cm path length quartz cuvette. CD spectra of ctDNA in the absence and presence of different concentrations of 11Cl were recorded at wavelengths ranging from 320 to 190 nm under constant nitrogen flush at 25 °C. For each CD spectrum, the number of scans was set to 5, and spectral data were collected at 0.1 ms intervals. Additionally, methyl green (MG) and 4′,6-diamidino-2-phenylindole (DAPI), and propidium iodide (PI) were analyzed as minor and major groove binders and as an intercalator, respectively, to help identify the binding mode of 11Cl to DNA. Samples were prepared in 10 mM Tris-HCl buffer (pH 7.6) with 1 mM EDTA. The concentration of ctDNA was fixed at 100 μM (concerning base pairs) and the ctDNA to 11Cl ratio was varied between 0 and 1. The samples were incubated for 24 h at 37 °C and then analyzed in the CD spectropolarimeter.

### Tandem mass spectrometry of 11Cl and DNA oligomers

11Cl was incubated with 14-mer ssDNA 5′–3′ (ATACATGGTACATA) and 6-mer dsDNA 5′–3′ (AGGCAG) in a 3 : 1 ratio at 37 °C in MilliQ water. After 24 h the solution was centrifuged with an Amicon® Ultra 0.5 mL centrifugal filter 3 kDa MWCO to remove the unreacted metal complex and excess salts. Mass spectra were recorded in an LTQ Orbitrap operating in negative mode and analyzed using Analysis of Oligonucleotide Modifications from Mass Spectra (Aom^2^s).^93^

### Molecular docking studies

Molecular docking studies were performed using AutoDock 4.2.^94^ The crystal structure of a B-DNA dodecamer d(CGCGAATTCGCG)2, 1BNA,^95^ the 12-mer with DAPI bound in the minor groove, 5T4W,^96^ and the 12-mer with an acridine-based intercalator, 1G3X,^97^ were obtained from the Protein Data Bank (PDB). The structure for 11Cl was obtained from the X-ray crystal structure with the counterion and solvent molecules removed to model 11^+^.

### Statistical analysis

Each cell viability result represents the mean ± standard deviation (SD) for *n* = 4 replicates in each of 3 independent assays, resulting in 12 independent values. Each apoptosis value determination represents the mean ± standard deviation of 2 independent assays. The IC_50_ values were obtained using GraphPad Prism software and ANOVA (analysis of variance) was performed to determine the statistical difference between the IC_50_ values of different cell lines.

## Author contributions

Sarah A. P. Pereira and Jan Romano-DeGea: Conceptualization, methodology, software, validation, formal analysis, investigation, data curation, writing – original draft, visualization. Ana Isabel Barbosa: Methodology, formal analysis, investigation, writing – original draft. Sofia A. Costa Lima: Methodology, writing – review & editing. Paul J. Dyson: Conceptualization, resources, writing – review & editing, supervision, funding acquisition. M. Lúcia M. F. S. Saraiva: Conceptualization, resources, writing – review & editing, supervision, project administration, funding acquisition.

## Conflicts of interest

There are no conflicts to declare.

## Supplementary Material

DT-052-D3DT02037A-s001

DT-052-D3DT02037A-s002
