# Supplementary material for: Fine-tuning the cytotoxicity of ruthenium(ii) arene compounds to enhance selectivity against breast cancers
Source: Dalton Trans. 2023 Jul 28;52(33):11679–90. doi: 10.1039/d3dt02037a (PMC10442743; doi:10.1039/d3dt02037a)
Supplement: DT-052-D3DT02037A-s001 [file DT-052-D3DT02037A-s001.pdf]

Electronic Supplementary Material (ESI) for Dalton Transactions.
This journal is © The Royal Society of Chemistry 2023

Supplementary Information

for

Fine-tuning the cytotoxicity of ruthenium(II) arene compounds to enhance selectivity against breast cancers

Sarah A.P. Pereira[1,2 †], Jan Romano-deGea[2 †], Ana Isabel Barbosa[1], Sofia A. Costa Lima[1], Paul J. Dyson[2*], M. Lúcia M. F. S. Saraiva[1*]

[1] LAQV, REQUIMTE, Departamento de Ciências Químicas, Faculdade de Farmácia, Universidade do Porto, Rua Jorge Viterbo Ferreira, nº 228, 4050-313 Porto, Portugal.

[2] Institut des Sciences et Ingénierie Chimiques, École Polytechnique Fédérale de Lausanne (EPFL), 1015 Lausanne, Switzerland

[†] These authors contributed equally to this work.

[*] Corresponding authors

E-mail address: paul.dyson@epfl.ch; lsaraiva@ff.up.pt

Tel.: +351 220428674; Fax: +351 226093483

Index

1. Synthesis and characterization of the screened compounds……………………......3

2. Solubility, partition coefficient and conductivity of 11CA……………………......12

3. Stability in solution……………………………………………………………14

4. Cytotoxicity studies……………………………………………………………21

5. Apoptosis effect of compounds tested in breast cancer cell lines…………………27

6. Reactivity with model biomolecules……………………………………………….28

7. Circular dichroism……………………………………………………………….38

8. Tandem mass spectrometry of 11Cl and DNA oligomers…………………………39

9. Molecular docking………………………………………………………………..43

10. NMR spectra of the synthesized compounds……………………………………46

1. Synthesis and characterization of the screened compounds

All organometallic manipulations were carried out under a nitrogen atmosphere using standard Schlenk techniques. CH_2Cl_2 was dried catalytically under nitrogen using a solvent purification system, manufactured by Innovative Technology Inc. All other solvents were dried over molecular sieves (3 Å) and saturated with nitrogen prior to use. [Ru(η^6-toluene)Cl_2]$_2$ [1], [Ru(η^6-toluene)(PPh_3)Cl_2] [2] and complexes **1** [3], **2** [3], **3** [4], **4** [5], **5** [6], **6** [6], **7** [7], **8** [8], and **9** [2] were prepared according to reported procedures and the characterization data were in agreement with those reported. Compounds **11**BF_4 [9] and **11**PF_6 [2] have been previously reported, but in this work, they have been prepared following a different synthetic route. All chemicals were either of reagent or analytical grade and used as purchased from commercial sources without additional purification. $RuCl_3{\cdot}3H_2O$ was obtained from Precious Metals Online. NMR spectra were acquired on a Bruker Avance 400 MHz spectrometer at room temperature unless otherwise stated. Chemical shifts are reported in ppm relative to $SiMe_4$ ($\delta = 0$) and coupling constants (J) are reported in Hz. The following abbreviations were used to designate multiplicities: s = singlet, d = doublet, t = triplet, m = multiplet, dd = doublet of doublets. High-resolution mass spectra (HRMS) were acquired by the MS service at EPFL, using either a Thermo Orbitrap Elite instrument with an LTQ-Orbitrap analyser or a Waters XEVO G2-S QTOF instrument with a QTOF analyser.

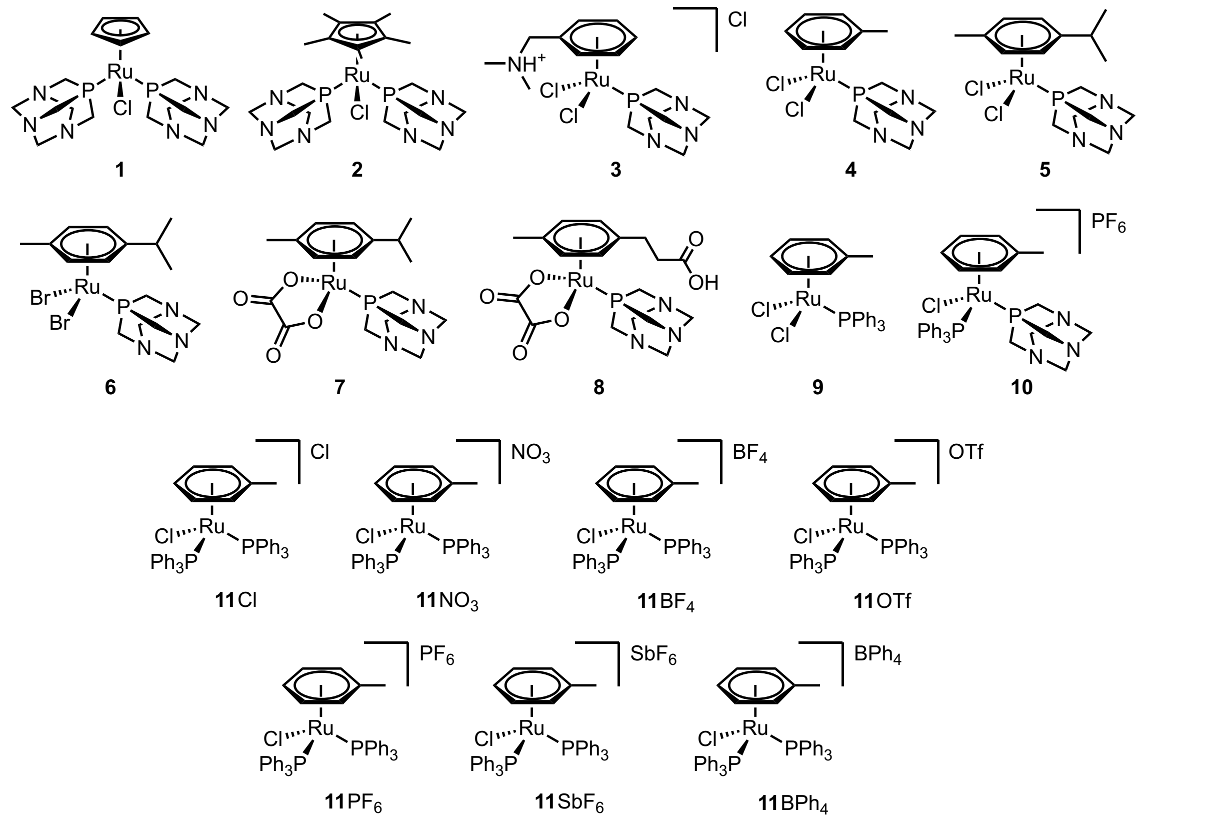

Figure S1. *Structures of the studied ruthenium arene compounds.*

[Ru(η^6-toluene)(PPh_3)(PTA)Cl]PF_6 **(10)**

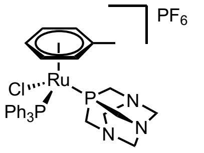

A suspension of [Ru(η^6-toluene)(PPh_3)Cl_2] (200 mg, 0.38 mmol, 1.0 eq.), 1,3,5-triaza-7-phosphaadamantane (90 mg, 0.57 mmol, 1.5 eq.) and ammonium hexafluorophosphate (99 mg, 0.61 mmol, 1.6 eq.) in a 1:1 mixture of MeOH:CH_2Cl_2 (20 mL) was stirred at 45°C for 1 h. The solvent was removed under reduced pressure, and the residue was taken in CH_2Cl_2 (30 mL). The organic phase was washed with H_2O (2 × 15 mL), dried over anhydrous Na_2SO_4 and the solvent removed under reduced pressure. The solid was dissolved in CH_2Cl_2 (5 mL), and the product was precipitated by the addition of pentane, filtered, washed with cold diethyl ether (2 × 5 mL), and dried under vacuum. Light yellow powder (106 mg, 35%). Light orange crystals suitable for X-ray diffraction were obtained by slow diffusion at 4°C of pentane into a solution of **10** in CH_2Cl_2.

^{1}H NMR (400 MHz, DMSO-*d*$_6$) δ: 7.66 – 7.53 (m, 9H), 7.54 – 7.44 (m, 6H), 6.60-6.55 (m, 1H) , 6.29 (d, ^3J = 5.9 Hz, 1H), 6.21 (d, ^3J = 6.3 Hz, 1H), 5.56-5.51 (m, 1H) , 4.35-4.27 (m, 4H), 4.25-4.21 (m, 2H), 4.21-4.19 (m, 1H) , 4.09 (ddd, $^{2,2,4}J$ = 14.8, 3.6, 1.5 Hz, 3H), 3.74 (ddd, $^{2,2,4}J$ = 14.8, 3.8, 1.6 Hz, 3H), 2.12 (d, ^4J = 1.1 Hz, 3H) ppm.

^{31}P{^{1}H} NMR (162 MHz, DMSO-*d$_6$*) δ: 37.9 (d, ^{2}J = 52.8 Hz), -34.8 (d, ^{2}J = 52.8 Hz), -139.4 (hept, $^{1}J_{FP}$ = 711.5 Hz) ppm.
^{19}F{^{1}H} NMR (376 MHz, DMSO-*d$_6$*) δ: -72.2 (d, $^{1}J_{PF}$ = 711.5 Hz) ppm.
^{13}C{^{1}H} NMR (101 MHz, DMSO-*d$_6$*) δ: 134.1 (d, ^{2}J = 9.8 Hz), 133.3 (d, ^{1}J = 48.2 Hz), 131.3, 128.8 (d, ^{3}J = 9.8 Hz), 117.5 (d, ^{2}J = 4.4 Hz), 97.2 (d, J = 8.1 Hz), 97.1 (d, ^{2}J = 3.6 Hz), 91.8 (d, ^{2}J = 8.6 Hz), 91.0, 83.1, 71.5 (d, ^{3}J = 7.4 Hz), 53.2 (d, ^{1}J = 15.3 Hz), 18.3 ppm.
^{31}P{^{1}H} NMR (162 MHz, Chloroform-*d*) δ: 31.6 (d, ^{2}J = 54.0 Hz), -40.7 (d, ^{2}J = 54.0 Hz) ppm.
HRMS (nanochip-ESI/LTQ-Orbitrap) m/z: $[M - PF_6^-]^+$ Calcd for $C_{31}H_{35}ClN_3P_2Ru^+$ 648.1033; Found 648.1014.

[Ru(η^6-toluene)(PPh_3)$_2$Cl]Cl **(11**Cl**)**

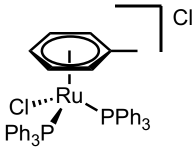

75 mg of **11**PF_6 (0.08 mmol) were dissolved in MeOH (50 mL). Amberlite® IRA-900 chloride form (5 g) was added, and the mixture was stirred for 12 h at room temperature. The solids were filtered off and another portion of Amberlite® IRA-900 chloride form (5g) was added to the filtrate. The suspension was stirred for 12 h more at room temperature. Then, the solids were removed by filtration and the filtrate concentrated under reduced pressure. The residue was dissolved in EtOH (5 mL), and the product was precipitated by the addition of Et_2O/hexane (2:1), filtered, and dried under vacuum. Counterion exchange was monitored and confirmed by the absence of PF_6 signals in the ^{31}P and ^{19}F NMR spectra and by the appearance of a signal in the ^{35}Cl NMR spectrum (bound chloride was not observed due to the quadrupolar moment of the nuclei). Dark orange crystalline powder (133 mg, 71 %). Light orange crystals suitable for X-ray diffraction were obtained by slow diffusion at 4°C of pentane into a solution of **11**Cl in CH_2Cl_2.
^{1}H NMR (400 MHz, Chloroform-*d*) δ: 7.45 – 7.33 (m, 18H), 7.31 – 7.22 (m, 12H), 5.93 – 5.88 (m, 2H), 5.86 – 5.81 (m, 1H), 4.65 (d, ^{3}J = 5.5 Hz, 2H), 2.07 (s, 3H) ppm.
^{31}P{^{1}H} NMR (162 MHz, Chloroform-*d*) δ: 22.8 ppm.
^{13}C{^{1}H} NMR (101 MHz, Chloroform-*d*) δ: 134.19 (t, ^{2}J = 4.6 Hz), 134.15 (d, ^{1}J = 48.0 Hz), 130.9, 128.6 (t, ^{3}J = 5.1 Hz), 122.9 (t, ^{2}J = 2.8 Hz), 99.3, 94.5 (t, ^{2}J = 4.9 Hz), 84.9, 19.5 ppm.

^{1}H NMR (400 MHz, Methanol-d_4) δ: 7.52 – 7.38 (m, 18H), 7.34 – 7.25 (m, 12H), 5.93 – 5.85 (m, 2H), 5.02 (d, ^3J = 6.1 Hz, 2H), 4.66 – 4.59 (m, 1H), 2.15 (s, 3H) ppm.

^{31}P{^{1}H} NMR (162 MHz, Methanol-d_4) δ: 22.1 ppm.

^{35}Cl NMR (39 MHz, Methanol-d_4) δ: -31 ppm.

HRMS (nanochip-ESI/LTQ-Orbitrap) m/z: [M – Cl^-]$^+$ Calcd for $C_{43}H_{38}ClP_2Ru^+$ 753.1175; Found 753.1155.

General Procedure for the synthesis of bis-triphenylphosphine ruthenium(II)-toluene cationic compounds (11CA)

A suspension of [Ru(η^6-toluene)(PPh_3)Cl_2] (100 mg, 0.19 mmol, 1.0 eq.), triphenylphosphine (55 mg, 0.21 mmol, 1.1 eq.) and the silver salt of the desired counterion (0.23 mmol, 1.2 eq.) in a 1:1 mixture of MeOH:CH_2Cl_2 (15 mL) was stirred at 45°C for 6 h while protected from light. The mixture was filtered through celite to remove silver salts. The solvent was removed under reduced pressure, and the residue dissolved in CH_2Cl_2 (5 mL). Finally, the product was precipitated by the addition of pentane, filtered, washed with cold diethyl ether (2 × 5 mL), and dried under vacuum.

[Ru(η^6-toluene)(PPh_3)$_2$Cl]BF_4 **(11**BF_4)

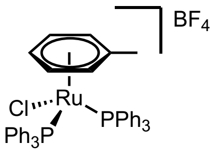

Light yellow powder (137 mg, 86%)

^{1}H NMR (400 MHz, Chloroform-*d*) δ: 7.44 – 7.34 (m, 18H), 7.31 – 7.22 (m, 12H), 5.84 – 5.75 (m, 2H), 5.41 – 5.33 (m, 1H), 4.63 (d, ^3J = 6.0 Hz, 2H), 2.07 (s, 3H) ppm.

^{31}P{^{1}H} NMR (162 MHz, Chloroform-*d*) δ: 22.4 ppm.

^{19}F{^{1}H} NMR (376 MHz, Chloroform-*d*) δ: -152.61, -152.67 ppm.

^{11}B{^{1}H} NMR (128 MHz, Chloroform-*d*) δ: -0.9 ppm.

^{13}C{^{1}H} NMR (101 MHz, Chloroform-*d*) δ: 133.99 (d, ^1J = 48.3 Hz), 133.98 (t, ^2J = 4.6 Hz), 130.9, 128.5 (t, ^3J = 5.0 Hz), 122.7 (t, ^2J = 1.5 Hz), 98.7, 94.4 (t, ^2J = 4.9 Hz), 83.7, 19.3 ppm.

HRMS (nanochip-ESI/LTQ-Orbitrap) m/z: [M – BF_4^-]$^+$ Calcd for $C_{43}H_{38}ClP_2Ru^+$ 753.1175; Found 753.1154.

[Ru(η^6-toluene)(PPh_3)$_2$Cl]OTf (**11**OTf)

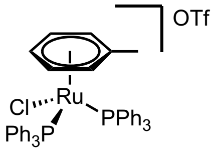

Orange crystalline powder (92 mg, 54 %). Intense yellow crystals suitable for X-ray diffraction were obtained by slow diffusion at 4°C of pentane into a solution of **11**OTf in CH_2Cl_2.

^{1}H NMR (400 MHz, Chloroform-*d*) δ: 7.44 – 7.34 (m, 18H), 7.30 – 7.21 (m, 12H), 5.84 – 5.76 (m, 2H), 5.32 – 5.25 (m, 1H), 4.67 (d, ^3J = 6.0 Hz, 2H), 2.08 (s, 3H) ppm.

^{31}P{^{1}H} NMR (162 MHz, Chloroform-*d*) δ: 22.4 ppm.

^{19}F{^{1}H} NMR (376 MHz, Chloroform-*d*) δ: -78.0 ppm.

^{13}C{^{1}H} NMR (201 MHz, Chloroform-*d*) δ: 134.1 (d, ^1J = 46.9 Hz), 134.1 (t, ^2J = 4.6 Hz), 131.0, 128.6 (t, ^3J = 5.0 Hz), 122.8 (t, ^2J = 2.8 Hz), 121.0 (q, $^1J_{FC}$ = 321.5 Hz), 98.7, 94.6 (t, ^2J = 5.0 Hz), 83.9, 19.4 ppm.

HRMS (nanochip-ESI/LTQ-Orbitrap) m/z: [M – OTf$^-$]$^+$ Calcd for $C_{43}H_{38}ClP_2Ru^+$ 753.1175; Found 753.1155.

[Ru(η^6-toluene)(PPh_3)$_2$Cl]PF_6 (**11**PF_6)

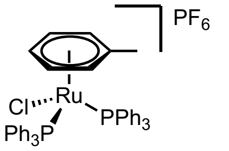

Dark yellow powder (151 mg, 89 %). Orange crystals suitable for X-ray diffraction were obtained by slow diffusion at 4°C of pentane into a solution of **11**PF_6 in CH_2Cl_2.

^{1}H NMR (400 MHz, Chloroform-*d*) δ: 7.44 – 7.35 (m, 18H), 7.30 – 7.21 (m, 12H), 5.80 – 5.71 (m, 2H), 5.10 – 5.03 (m, 1H), 4.67 (d, ^3J = 6.0 Hz, 2H), 2.08 (s, 3H) ppm.

^{31}P{^{1}H} NMR (162 MHz, Chloroform-*d*) δ: 22.2, -144.4 (hept, $^1J_{FP}$ = 713.0 Hz) ppm.

^{19}F{^{1}H} NMR (376 MHz, Chloroform-*d*) δ: -72.8 (d, $^1J_{PF}$ = 713.0 Hz) ppm.

^{13}C{^{1}H} NMR (101 MHz, Chloroform-*d*) δ: 134.1 (d, ^1J = 44.1 Hz), 134.0 (t, ^2J = 4.6 Hz), 131.1, 128.6 (t, ^3J = 5.1 Hz), 122.8, 98.5, 94.7, 83.8, 19.4 ppm.

HRMS (nanochip-ESI/LTQ-Orbitrap) m/z: [M – PF_6^-]$^+$ Calcd for $C_{43}H_{38}ClP_2Ru^+$ 753.1175; Found 753.1155.

[Ru(η^6-toluene)(PPh_3)$_2$Cl]SbF_6 (**11**SbF_6)

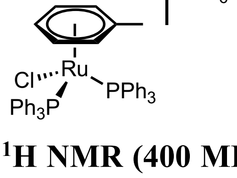

Light red crystalline powder (133 mg, 71 %). Intense yellow crystals suitable for X-ray diffraction were obtained by slow diffusion at 4°C of pentane into a solution of **11**SbF_6 in CH_2Cl_2.

^{1}H NMR (400 MHz, Chloroform-*d*) δ: 7.44 – 7.35 (m, 18H), 7.30 – 7.21 (m, 12H), 5.78 – 5.70 (m, 2H), 4.89 – 4.84 (m, 1H), 4.71 (d, ^3J = 6.0 Hz, 2H), 2.10 (s, 3H) ppm.

^{31}P{^{1}H} NMR (162 MHz, Chloroform-*d*) δ: 22.1 ppm.

^{19}F{^{1}H} NMR (376 MHz, Chloroform-*d*) δ: -106.27 – -139.07 (m) ppm.

^{13}C{^{1}H} NMR (101 MHz, Chloroform-*d*) δ: 134.03 (d, ^{1}J = 48.3 Hz), 134.01 (t, ^{2}J = 4.6 Hz), 131.1, 128.6 (t, ^{3}J = 5.1 Hz), 122.7 (t, ^{2}J = 2.7 Hz), 98.2, 94.8 (t, ^{2}J = 5.0 Hz), 83.5, 19.3 ppm.

HRMS (nanochip-ESI/LTQ-Orbitrap) m/z: [M – SbF_6^-]$^+$ Calcd for $C_{43}H_{38}ClP_2Ru^+$ 753.1175; Found 753.1159.

[Ru(η^6-toluene)(PPh_3)$_2$Cl]BPh_4 **(11BPh_4)**

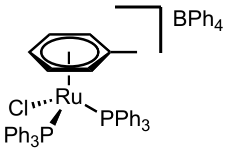

Light orange crystalline needles (158 mg, 78 %). Light Orange crystals suitable for X-ray diffraction were obtained by slow diffusion at 4°C of pentane into a solution of **11**BPh_4 in CH_2Cl_2.

^{1}H NMR (400 MHz, Chloroform-*d*) δ: 7.42-7.35 (m, 8H) , 7.34 – 7.26 (m, 12H), 7.24 – 7.13 (m, 18H), 6.91-6.85 (m, 8H) , 6.82-6.76 (m, 4H), 5.11 – 4.99 (m, 2H), 4.43 (d, ^{3}J = 6.0 Hz, 2H), 3.73 – 3.63 (m, 1H), 1.98 (s, 3H) ppm.

^{31}P{^{1}H} NMR (162 MHz, Chloroform-*d*) δ: 22.4 ppm.

^{11}B{^{1}H} NMR (128 MHz, Chloroform-*d*) δ: -6.8 ppm.

^{13}C{^{1}H} NMR (201 MHz, Chloroform-*d*) δ: 134.9, 134.2 (t, ^{2}J = 4.6 Hz), 134.2 (d, ^{1}J = 50.8 Hz), 131.0, 128.7 (t, ^{3}J = 5.1 Hz), 128.5, 122.9 (d, ^{2}J = 1.3 Hz), 119.6, 115.9, 99.3, 94.5, 85.5, 19.5 ppm.

HRMS (nanochip-ESI/LTQ-Orbitrap) m/z: [M – BPh_4^-]$^+$ Calcd for $C_{43}H_{38}ClP_2Ru^+$ 753.1175; Found 753.1153.

[Ru(η^6-toluene)(PPh_3)$_2$Cl]NO_3 **(11NO_3)**

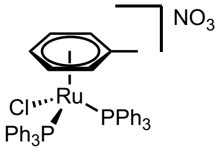

A suspension of [Ru(η^6-toluene)(PPh_3)Cl_2] (100 mg, 0.19 mmol, 1.0 eq.), triphenylphosphine (55 mg, 0.21 mmol, 1.1 eq.) and $NaNO_3$ (19 mg, 0.23 mmol, 1.2 eq.) in CH_2Cl_2 (15 mL) was stirred at room temperature for 24 h. The mixture was filtered through celite to remove the solids. The filtrate was concentrated under reduced pressure (5 mL), and the product was precipitated by the addition of pentane, filtered, washed with cold diethyl ether (2 × 5 mL), and, finally, dried under vacuum. Light yellow cream powder (66 mg, 43%).

^{1}H NMR (400 MHz, Chloroform-*d*) δ: 7.33 – 7.27 (m, 18H), 7.20 – 7.12 (m, 12H), 5.80 – 5.76 (m, 2H), 5.61 – 5.54 (m, 1H), 4.54 (d, ^{3}J = 5.8 Hz, 2H), 1.97 (s, 3H) ppm.

$^{31}P\{^1H\}$ NMR (162 MHz, Chloroform-*d*) δ: 22.6 ppm.

$^{13}C\{^1H\}$ NMR (101 MHz, Chloroform-*d*) δ: 134.2 (t, $^2J = 4.6$ Hz), 134.1 (d, $^1J = 46.4$ Hz), 131.0, 128.6 (t, $^3J = 5.1$ Hz), 122.9, 99.0, 94.5 (t, $^2J = 4.0$ Hz), 89.1, 19.5 ppm.

HRMS (nanochip-ESI/LTQ-Orbitrap) m/z: $[M - NO_3^-]^+$ Calcd for $C_{43}H_{38}ClP_2Ru^+$ 753.1175; Found 753.1156.

Single clear light orange or intense yellow crystals of **10**, **11**Cl, **11**OTf, **11**PF_6, **11**SbF_6 and **11**BPh_4 were obtained and measured. Suitable crystals with dimensions 0.09 × 0.08 × 0.05 mm^3 (**10**), 0.14 × 0.12 × 0.08 mm^3 (**11**Cl), 0.12 × 0.09 × 0.06 mm^3 (**11**OTf), 0.51 × 0.09 × 0.06 mm^3 (**11**PF_6), 0.19 × 0.12 × 0.06 mm^3 (**11**SbF_6) and 0.10 × 0.03 × 0.03 mm^3 (**11**BPh_4) were selected and mounted on a XtaLAB Synergy R, DW system, HyPix-Arc 150 diffractometer or on a SuperNova, Dual, Cu at home/near, AtlasS2 diffractometer. The crystals were kept at a steady T = 140.00(10) K during data collection. The structures were solved with the **ShelXT** (Sheldrick, 2015) solution program using dual methods and by using **Olex2** 1.5 (Dolomanov et al., 2009) as the graphical interface. The models were refined with **ShelXL** 2018/3 (Sheldrick, 2015) using full matrix least squares minimization on $\boldsymbol{F^2}$.

Table S1. *Selected bond lengths (Å) and angles (°) for **11Cl**, **11BF**$_4$ ^{a}, **11OTf**, **11PF**$_6$, **11SbF**$_6$ and **11BPh**$_4$. ^{a} Extracted from reference [9].*

	**11**Cl	**11**BF_4 ^{a}	**11**OTf	**11**PF_6	**11**SbF_6	**11**BPh_4
**Ru1-Cl1**	2.391(2)	2.389(3)	2.4069(4)	2.396(1)	2.4020(9)	2.3913(6)
**Ru1-P1**	2.362(2)	2.399(3)	2.3759(4)	2.3643(14)	2.379(1)	2.3632(6)
**Ru1-P2**	2.375(1)	2.384(3)	2.3730(4)	2.3668(13)	2.373(1)	2.3803(6)
**Ru1-C1**	2.327(6)	2.257(4)	2.3326(18)	2.321(5)	2.332(4)	2.321(2)
**Ru1-C2**	2.278(5)	2.247(4)	2.2932(19)	2.267(6)	2.260(4)	2.266(2)
**Ru1-C3**	2.242(6)	2.219(4)	2.2169(18)	2.256(5)	2.225(4)	2.260(2)
**Ru1-C4**	2.222(7)	2.214(5)	2.2191(17)	2.215(5)	2.219(3)	2.243(3)
**Ru1-C5**	2.215(7)	2.305(5)	2.2311(17)	2.223(5)	2.212(4)	2.227(3)
**Ru1-C6**	2.282(7)	2.347(4)	2.2709(17)	2.307(5)	2.285(4)	2.302(2)
**Ru1-C_{avg}**	2.26(4)	2.26(5)	2.26(5)	2.26(4)	2.26(5)	2.27(4)
**Cl1-Ru1-P1**	89.76(6)	85.3(l)	91.682(14)	89.78(4)	91.94(3)	87.68(2)
**Cl1-Ru1-P2**	85.60(5)	89.3(l)	86.740(14)	89.06(5)	86.10(3)	88.86(2)
**P1-Ru1-P2**	98.35(5)	100.3(1)	97.894(14)	97.05(5)	97.44(3)	98.34(2)

Table S2. *Crystallographic data for **10**, **11**Cl, **11**OTf, **11**PF_6, **11**SbF_6, and **11**BPh_4.*

**Compound**	**10**	**11**Cl	**11**OTf	**11**PF_6	**11**SbF_6	**11**BPh_4
Formula	$C_{31}H_{35}ClF_6N_3P_3Ru$	$C_{43.5}H_{43}Cl_3O_2P_2Ru$	$C_{44}H_{38}ClF_3O_3P_2RuS$	$C_{44}H_{40}Cl_3F_6P_3Ru$	$C_{43}H_{38}ClF_6P_2RuSb$	$C_{67}H_{58}BClP_2Ru$
$D_{calc.}$/ g cm^{-3}	1.653	1.507	1.558	1.508	1.721	1.363
m/mm^{-1}	6.739	6.335	5.705	6.174	1.319	3.798
Formula Weight	793.05	867.14	902.26	983.09	988.94	1072.40
Colour	clear light orange	clear light orange	clear intense yellow	clear intense yellow	clear intense orange	clear light orange
Shape	prism-shaped	irregular-shaped	prism-shaped	needle-shaped	prism-shaped	prism-shaped
Size/mm^3	0.09×0.08×0.05	0.14×0.12×0.08	0.12×0.09×0.06	0.51×0.09×0.06	0.19×0.12×0.06	0.10×0.03×0.03
T/K	140.00(10)	140.00(10)	139.99(10)	140.00(10)	140.00(10)	140.00(10)
Crystal System	orthorhombic	triclinic	monoclinic	orthorhombic	monoclinic	monoclinic
Flack Parameter	0.143(7)			-0.014(4)		-0.0220(16)
Space Group	$P2_12_12_1$	*P*-1	$P2_1/c$	$P2_12_12_1$	$P2_1/c$	$P2_1$
a/Å	9.30092(8)	10.8812(5)	12.47399(8)	15.10287(19)	12.3243(4)	10.47706(6)
b/Å	10.67827(8)	11.0631(5)	10.42115(6)	16.6186(2)	10.3143(3)	16.55786(10)
c/Å	32.0935(2)	16.8995(8)	30.03284(18)	17.2511(3)	30.4650(9)	15.24231(9)
a/°	90	74.198(4)	90	90	90	90
b/°	90	78.406(4)	99.8863(6)	90	99.752(3)	98.9266(5)
g/°	90	82.343(4)	90	90	90	90
V/Å^3	3187.46(4)	1910.88(15)	3846.10(4)	4329.83(10)	3816.6(2)	2612.17(3)
Z	4	2	4	4	4	2
Z'	1	1	1	1	1	1
Wavelength/Å	1.54184	1.54184	1.54184	1.54184	0.71073	1.54184
Radiation type	Cu K_a	Cu K_a	Cu K_a	Cu K_a	Mo K_a	Cu K_a

Q_{min}/°	2.754	2.759	2.987	3.693	2.749	2.935
Q_{max}/°	76.220	75.557	76.592	72.474	29.599	76.069
Measured Refl's.	33963	12093	40600	19149	28482	59800
Indep't Refl's	6575	12093	7930	8383	9225	10685
Refl's I≥2*s*(I)	6374	10369	7431	7948	6469	10430
R_{int}	0.0282	.	0.0176	0.0279	0.0827	0.0268
Parameters	463	492	571	543	553	650
Restraints	240	29	250	61	288	1
Largest Peak/e Å^{-3}	0.638	1.950	0.669	1.071	0.945	0.239
Deepest Hole/e Å^{-3}	-0.346	-1.801	-0.381	-0.510	-1.274	-0.398
GooF	1.038	1.054	1.028	1.049	0.989	1.044
wR_2 (all data)	0.0517	0.2149	0.0583	0.1025	0.0947	0.0428
wR_2	0.0513	0.2007	0.0574	0.0998	0.0821	0.0426
R_1 (all data)	0.0226	0.0825	0.0245	0.0404	0.0835	0.0190
R_1	0.0215	0.0725	0.0226	0.0376	0.0503	0.0181
CCDC number	**2201998**	**2210246**	**2193598**	**2210241**	**2210245**	**2202259**

2. Solubility, partition coefficient and conductivity of **11**CA

0.5 mg of compounds **10** and **11**CA were dispersed in 0.5 mL of MilliQ water, and, after sonication, the mixtures were stirred at 25ºC for 1 h. The saturated solutions were filtered to remove any insoluble solids, dissolved in 0.5 mL of MilliQ water, and analyzed using ICP-MS. Samples were submitted to acidic digestion with 2 mL of concentrated HNO3 (69%, ROTIPURAN Supra, Roth) in PP digestion vials using a heating block system (DigiPREP Jr. 15ml, 40 Pos, SCP Science). The digestion program was the following: 15 min to heat to 100°C and keep at 100°C for 60 min. After the digestion, the sample volumes were precisely adjusted to 5 mL with MilliQ water. Samples were further diluted 30 times with 2% HNO3 solution and their Ru content was analyzed by ICP-MS using standard mode on NexIon 350 D ICP-MS instrument (PerkinElmer). Yttrium was added as an internal standard at a concentration of 2 ppb to all the solution and Ru quantitation was performed using an external calibration curve with standards in the 0.05-50 ppb range. All measurements were performed in triplicates.

The *n*-octanol-water partition ratio is the most common way of identifying the lipophilicity of a compound. This assay was performed following the Shake-flask method [10]. To a 0.1 mM of **11**CA in a water-saturated n-octanol solution was added n-octanol-saturated MilliQ water in different organic: aqueous ratios (1:1; 1:2 and 2:1). The mixture was shaken for 2 minutes, and after that, the solution was centrifuged to separate the organic and aqueous phase. Aliquots of each phase were taken and analyzed separately by ICP-MS. Samples were submitted to acidic digestion with 2 mL of concentrated HNO_3 (69%, ROTIPURAN Supra, Roth) in PP digestion vials using a heating block system (DigiPREP Jr. 15ml, 40 Pos, SCP Science). The digestion program was the following: 15 min to heat up to 100°C and keep at 100°C for 60 min. After the digestion, the sample volumes were precisely adjusted to 5 mL with MilliQ water. Samples were further diluted 30 times with 2% HNO3 solution and their Ru content was analyzed by ICP-MS using standard mode on NexIon 350 D ICP-MS instrument (PerkinElmer). Yttrium was added as an internal standard at a concentration of 2 ppb to all the solution and Ru quantitation was performed using an external calibration curve with standards in the 0.05-50 ppb range. The partition coefficient was calculated as $P_{ow} = [Ru]_{org_f} / \left([Ru]_{org_i} - [Ru]_{org_f} \right)$, where $[Ru]_{org_i}$ and $[Ru]_{org_f}$ are the concentrations of ruthenium in the organic phase before and after mixing with the aqueous phase, respectively. All measurements were performed in triplicates.

The molar conductivity (Λ_M, reported as $S{\cdot}cm^2{\cdot}mol^{-1}$) of solutions of **11**CA in acetonitrile (1 mM) were measured in a Radiometer Copenhagen CDM92 equipped with a conductivity cell CDC641T glass platinum electrode at room temperature (25 °C). The conductivity of the pure solvent was used as standard controls.

Table S3. *Solubility in water at 25 °C, n-octanol/water partition coefficient and molar conductivity of the **11**CA.* * *below limit of detection = 5 ppt.*

	Solubility (µg/mL)	Solubility (µM)	$LogP_{ow}$	Conductivity ($S{\cdot}cm^2$/mol)
1	40 x10^3 [4]	77 x10^3	−1.85 [11]	
2	25 x10^3 [4]	43 x10^3		
3	> 10 x10^3 [12]	> 20 x10^3		
4			−1.5 [13]	
5	~10 x10^3 [14]	~22 x10^3	> −1.8 [13]	
6				
7	> 48 x10^3	> 100 x10^3 [7]		
8		> 25 x10^3 [15]	−1.57 [8]	
9	34 ± 4	65 ± 8		
10	475 ± 102	547 ± 117		
11Cl	416 ± 70	528 ± 88	1.48 ± 0.29	128
11NO₃	218 ± 34	268 ± 42	1.57 ± 0.29	141
11BF₄	4 ± 1	5 ± 2	1.60 ± 0.20	124
11OTf	1.0 ± 0.1	1.1 ± 0.1	1.75 ± 0.18	173
11PF₆	0.6 ± 0.2	0.6 ± 0.2	1.91 ± 0.23	150
11SbF₆	below LOD *	below LOD *	2.01 ± 0.07	186
11BPh₄	below LOD *	below LOD *	> 3.5	71

Table S4. *Molar Gibbs energies of hydration of selected counteranions calculated using the method described in reference* [16]*. The radius, r; width of hydration shell, Δr; number of water molecules in this shell, n; the neutral contribution,* $\Delta G_{neutral}$*; and the electrostatic contribution,* ΔG_{el1+2}

$$[\mathbf{11}]CA \xrightleftharpoons{\Delta G_{hyd}} [\mathbf{11}]^+ + CA^-$$

	**r** (nm)	**n**	**Δr** (nm)	$\Delta G_{neutral}$ (KJ/mol)	ΔG_{el1+2} (KJ/mol)	$\Delta G_{hyd}{}^*{}_{calc}$ (KJ/mol)
**Cl^-**	0.168	2.1	0.050	+60	−331	**−291**
**NO_3^-**	0.200	1.8	0.034	+72	−293	**−245**
**BF_4^-**	0.211	1.7	0.029	+76	−281	**−231**
**OTf^-**	0.230	1.6	0.024	+84	−263	**−206**
**PF_6^-**	0.242	1.5	0.021	+90	−252	**−191**

**SbF_6^-**	0.252	1.4	0.018	+95	−243	**−178**
**BPh_4^-**	0.421	0.9	0.004	+217	−150	**+67**

3. Stability in solution

Stability studies were performed on **11**CA over 72 h at 37 °C. The stability of the complexes **11**CA was studied via ESI-MS. The complexes were dissolved in DMSO and the solution was diluted with MilliQ water to reach 0.1% DMSO in water (final concentration, 10 µM). Aliquots were taken and injected into the spectrometer without any further sample dilution or preparation. The stability of **11**Cl was also monitored over 72 h via ^{1}H and ^{31}P NMR spectroscopy. The complex was fully dissolved in d_6-DMSO, diluted with D_2O to reach 10% d_6-DMSO in D_2O (1 mM final concentration) and, finally, the pD was adjusted to 7.2 ± 0.1 (neutral conditions). Furthermore, the stability of **11**Cl and **11**BPh_4 was also assessed in 100% d_6-DMSO (1 mM final concentration) as a highly coordinating solvent.

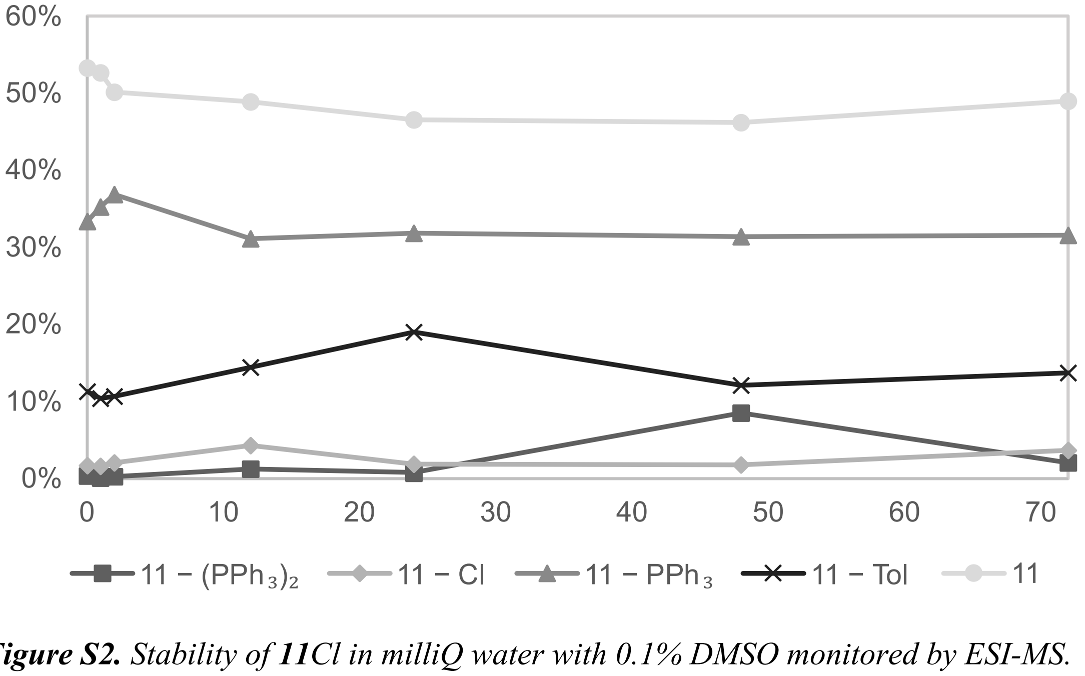

Figure S2. *Stability of **11**Cl in milliQ water with 0.1% DMSO monitored by ESI-MS.*

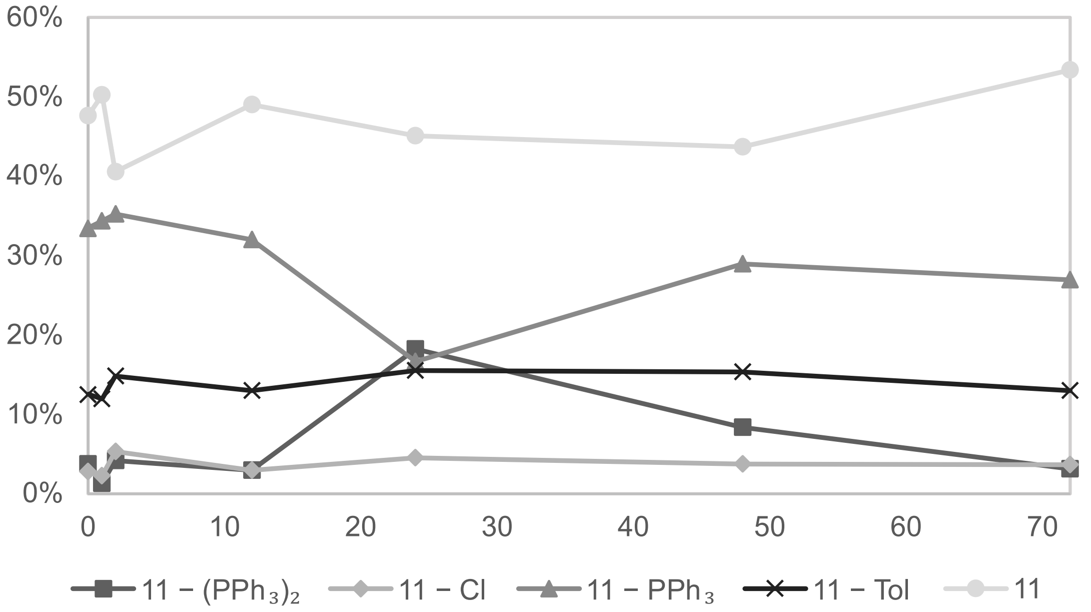

Figure S3. *Stability of **11**NO_3 in milliQ water with 0.1% DMSO monitored by ESI-MS.*

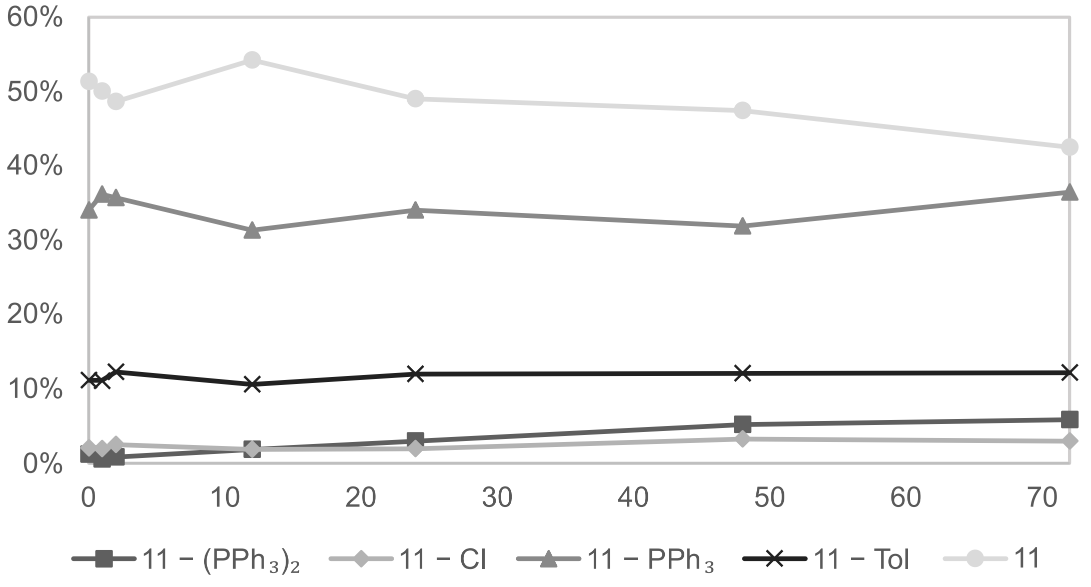

Figure S4. *Stability of **11**BF_4 in milliQ water with 0.1% DMSO monitored by ESI-MS.*

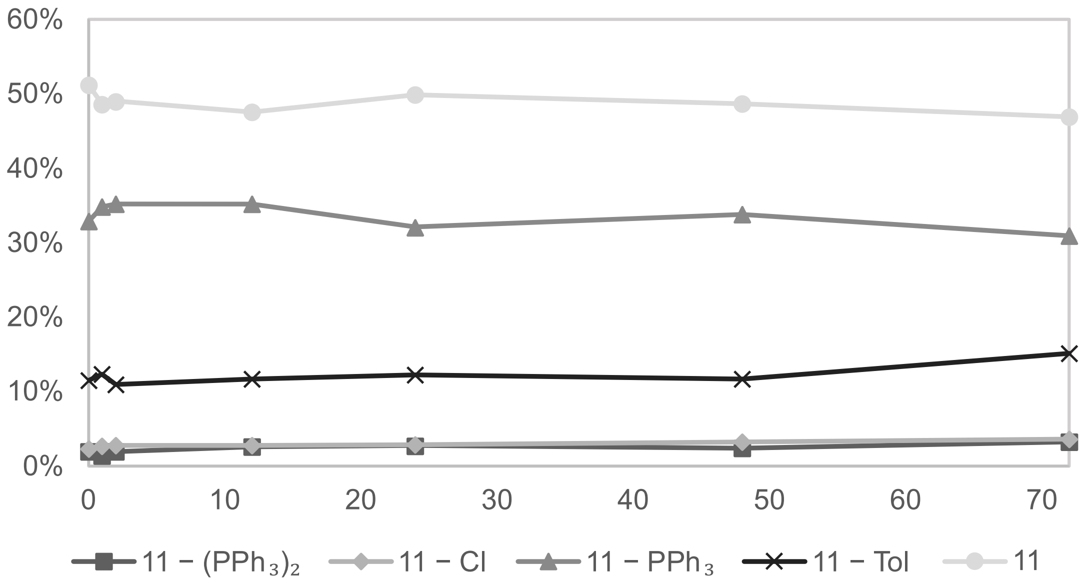

Figure S5. *Stability of **11**OTf in milliQ water with 0.1% DMSO monitored by ESI-MS.*

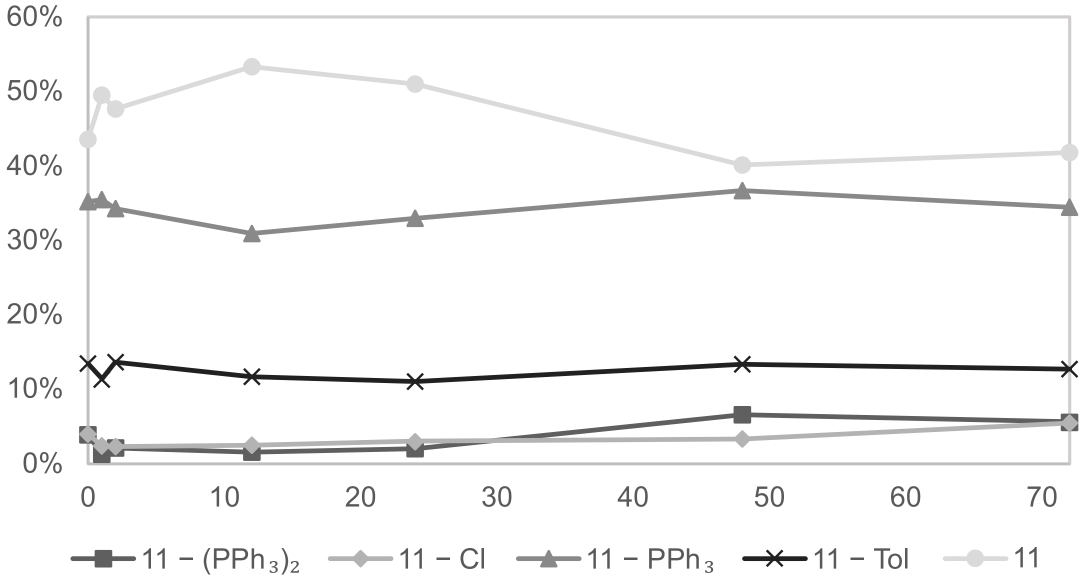

Figure S6. *Stability of **11**PF_6 in milliQ water with 0.1% DMSO monitored by ESI-MS.*

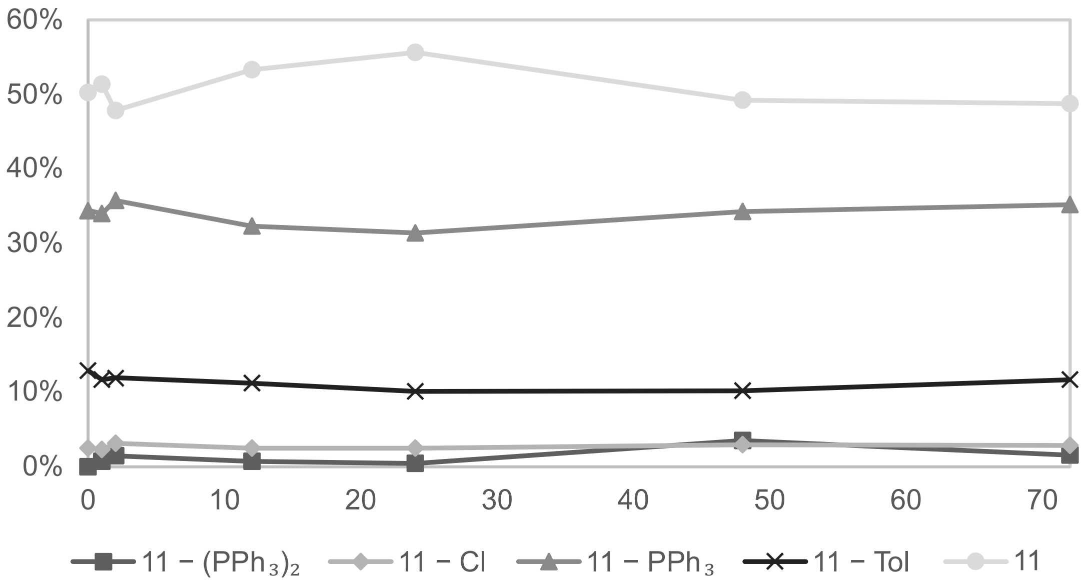

Figure S7. *Stability of **11**SbF$_6$ in milliQ water with 0.1% DMSO monitored by ESI-MS.*

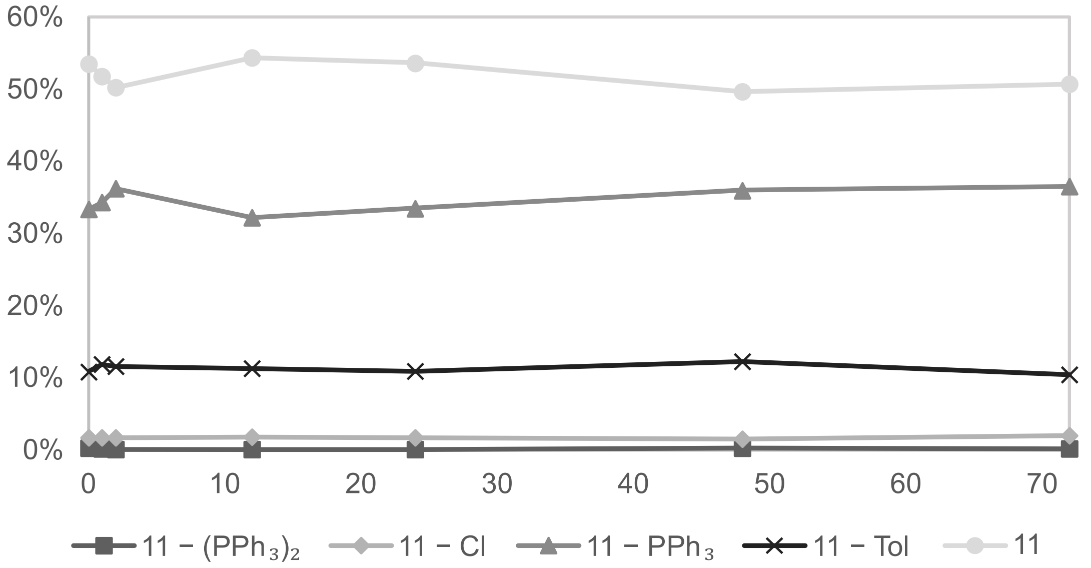

Figure S8. *Stability of **11**BPh$_4$ in milliQ water with 0.1% DMSO monitored by ESI-MS.*

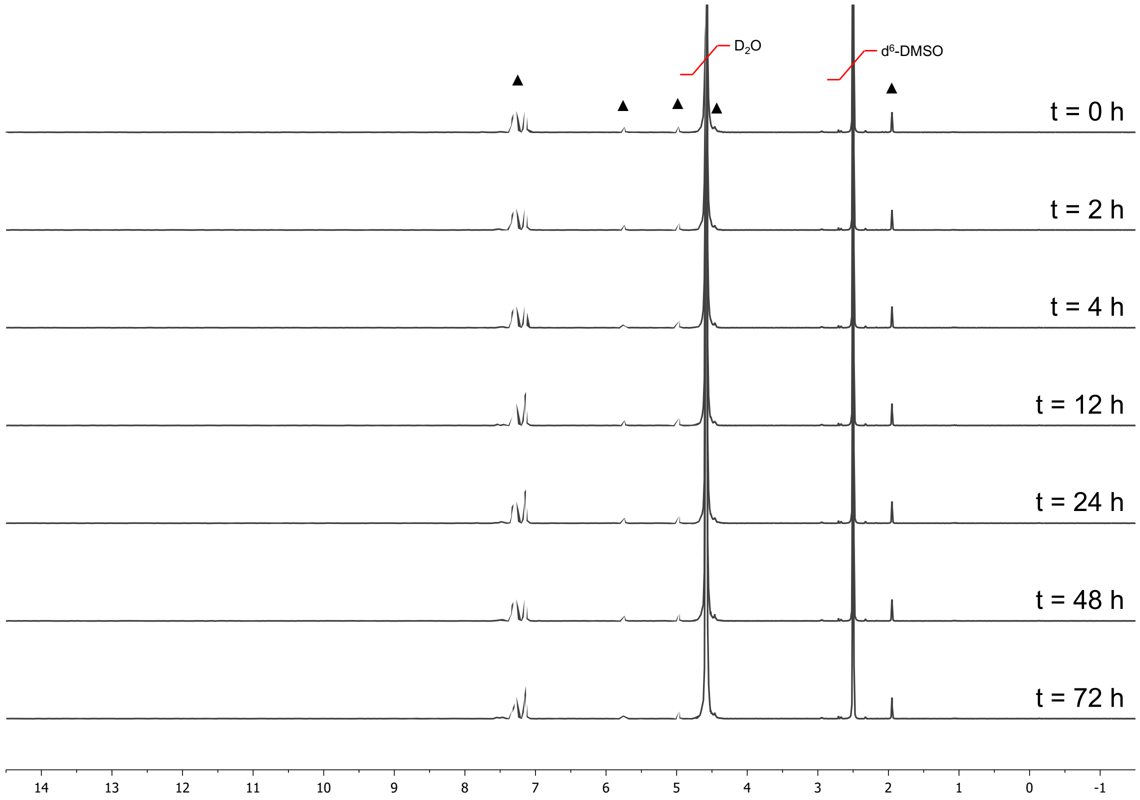

Figure S9. *Stability of **11**Cl (▲) in a D_2O:d_6-DMSO mixture (90:10) at pD = 7.2 ± 0.2 monitored by ^1H NMR.*

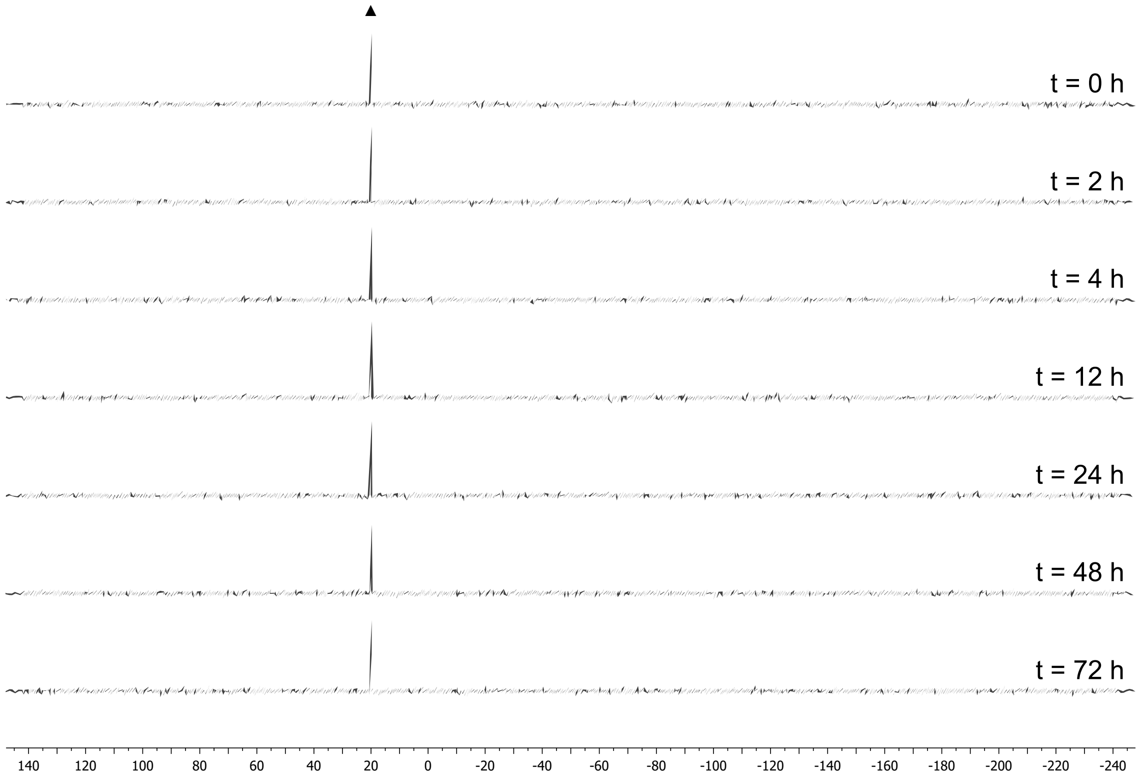

Figure S10. *Stability of **11**Cl (▲) in a D_2O:d_6-DMSO mixture (90:10) at pD = 7.2 ± 0.2 monitored by ^{31}P NMR.*

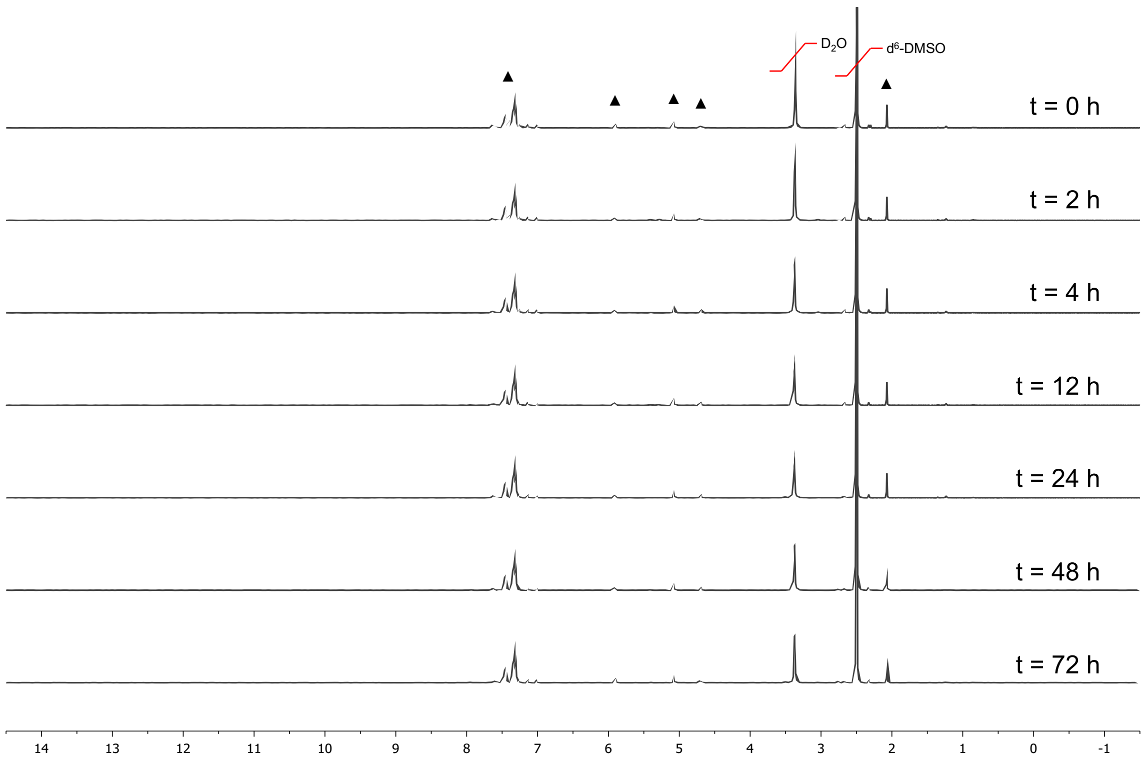

Figure S11. *Stability of **11**Cl (▲) in d_6-DMSO monitored by ^{1}H NMR.*

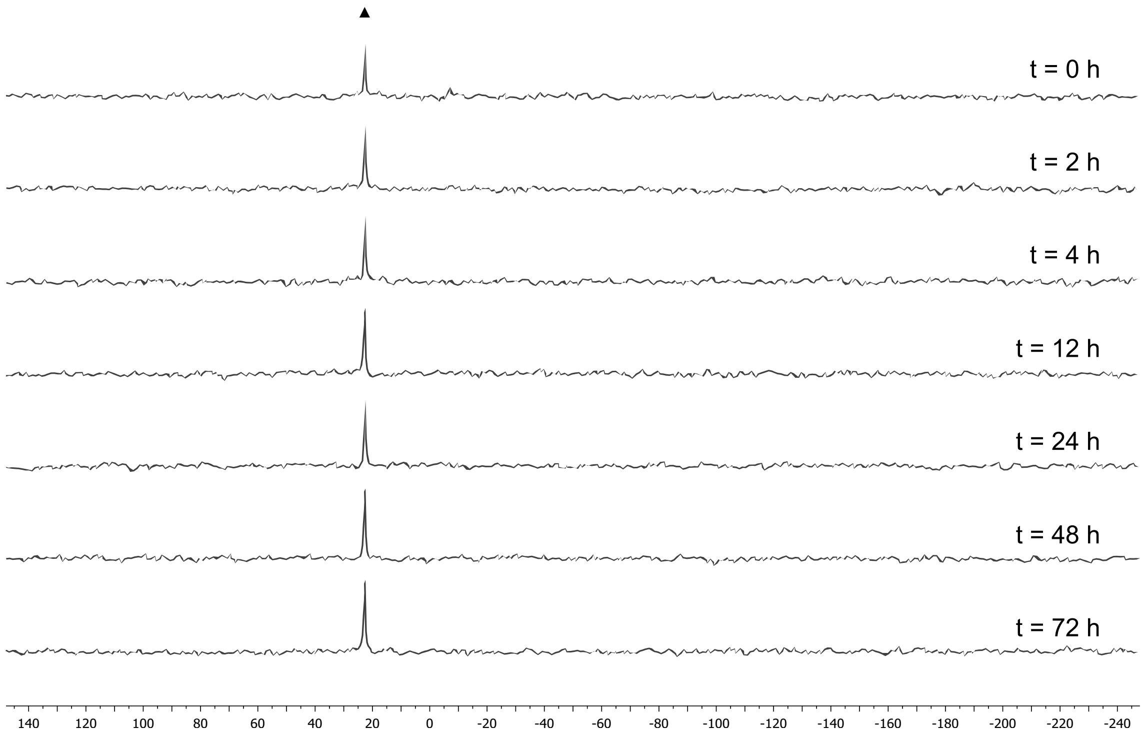

Figure S12. *Stability of **11**Cl (▲) in d_6-DMSO monitored by ^{31}P NMR.*

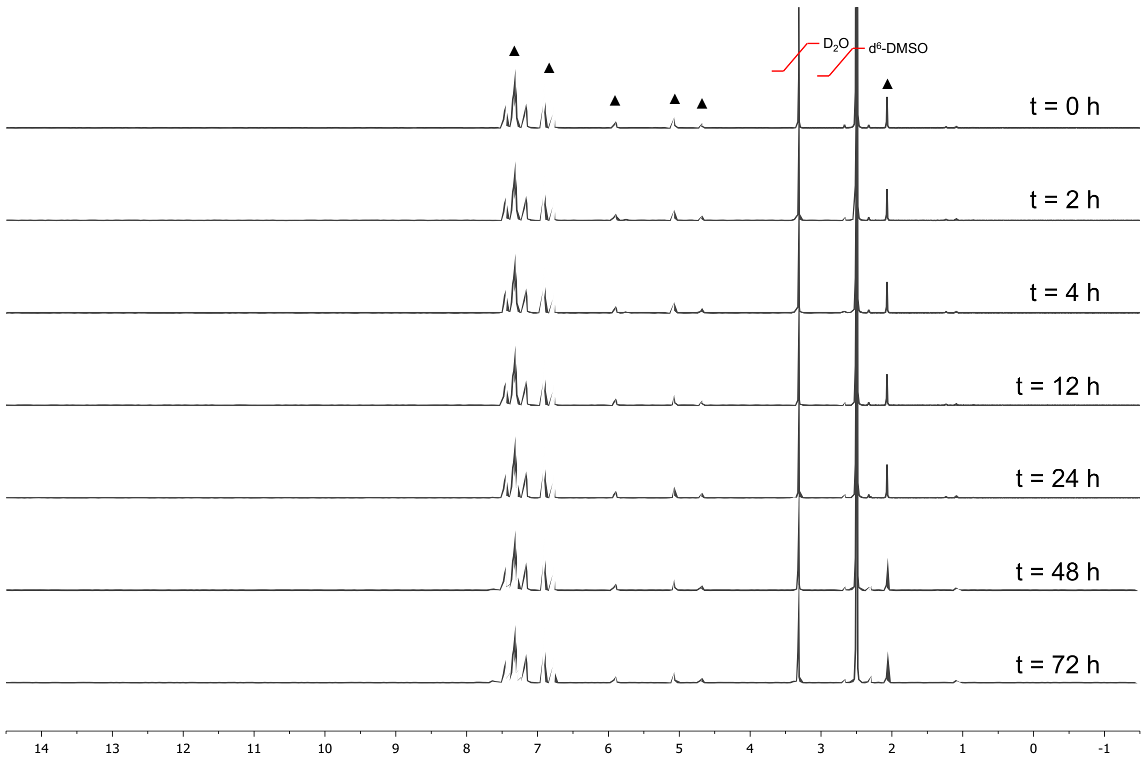

Figure S13. *Stability of **11**BPh$_4$ (▲) in d$_6$-DMSO monitored by ^{1}H NMR.*

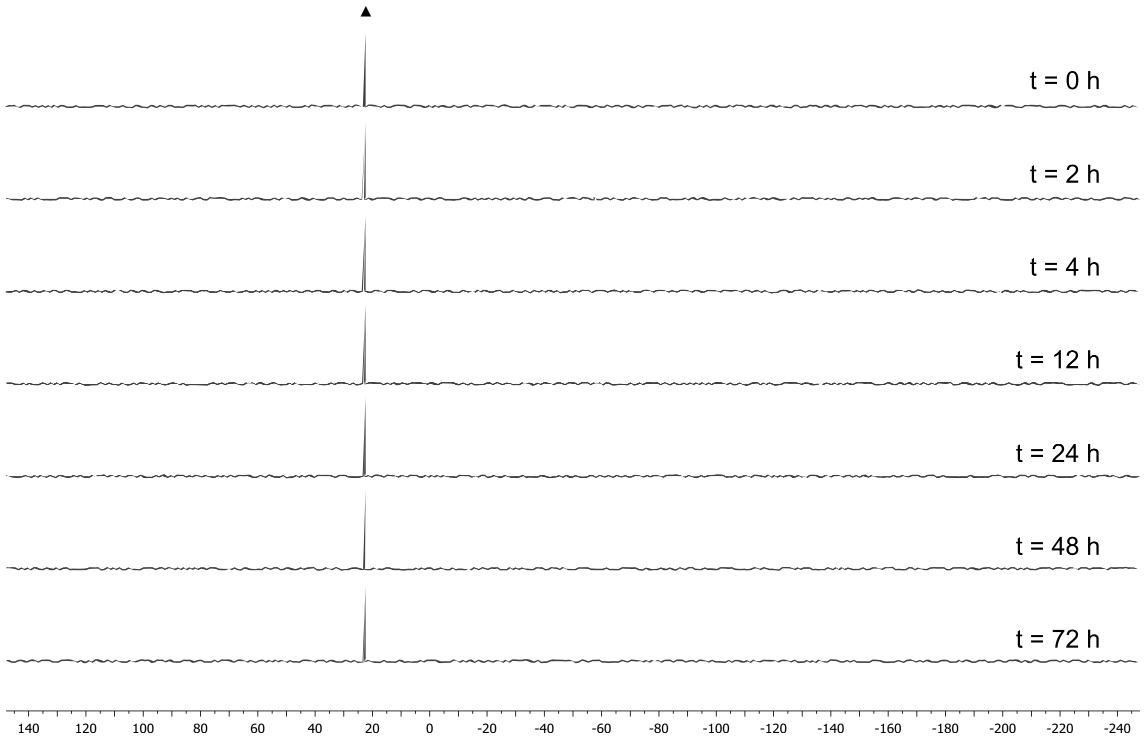

Figure S14. *Stability of **11**BPh$_4$ (▲) in d$_6$-DMSO monitored by ^{31}P NMR.*

4. Cytotoxicity studies

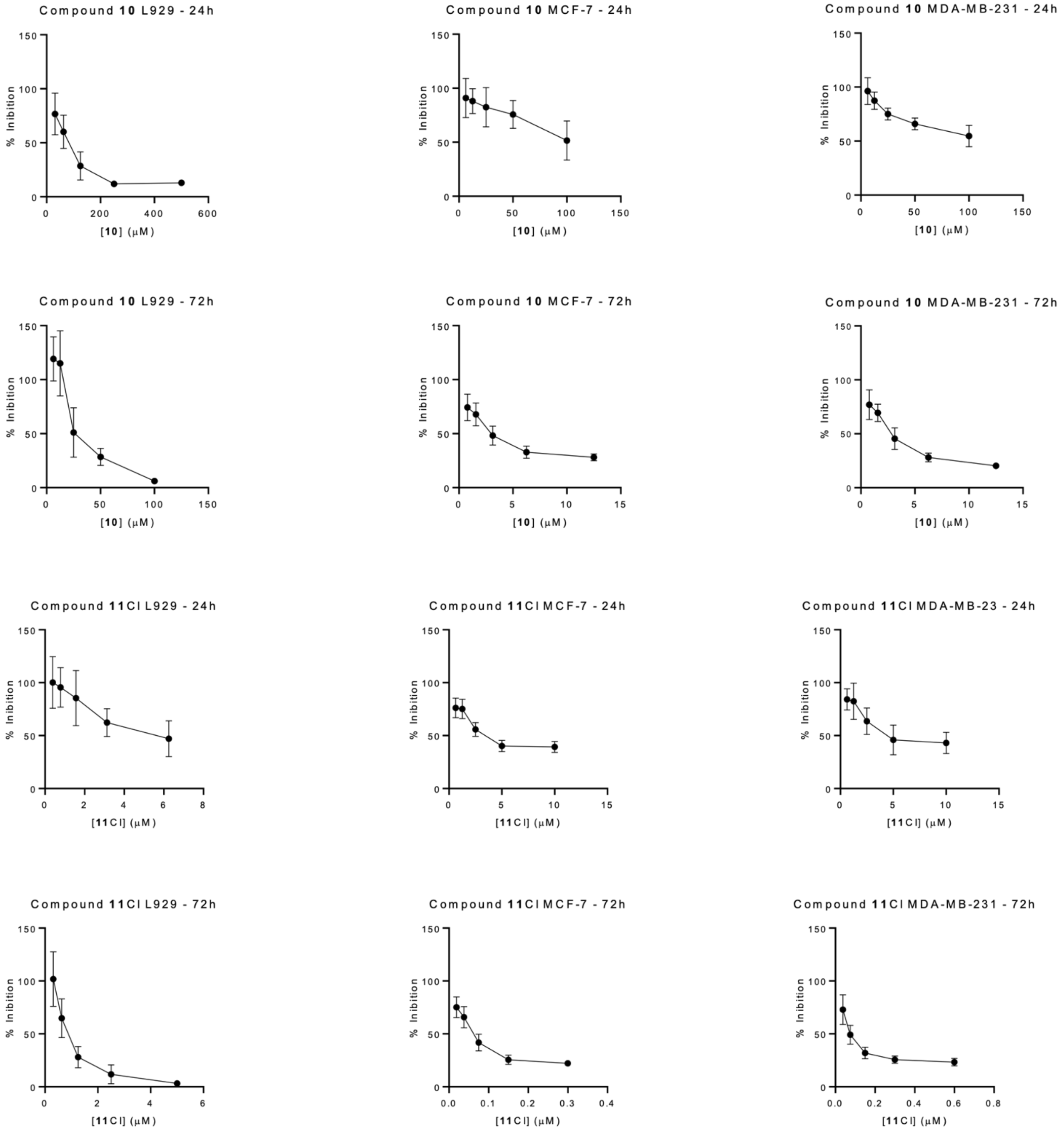

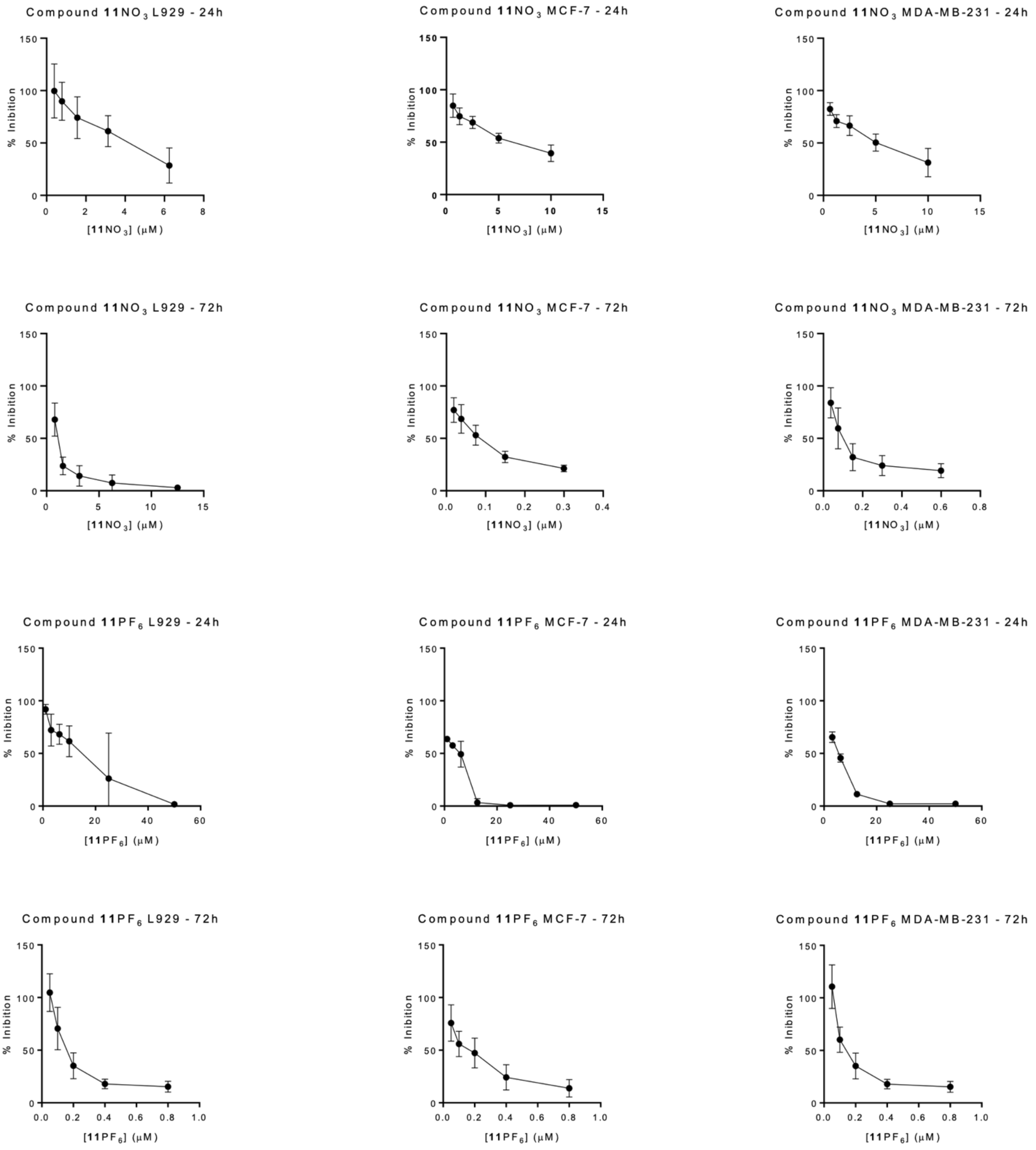

Compound 11NO_3 L929 - 24h
Compound 11NO_3 MCF-7 - 24h
Compound 11NO_3 MDA-MB-231 - 24h
Compound 11NO_3 L929 - 72h
Compound 11NO_3 MCF-7 - 72h
Compound 11NO_3 MDA-MB-231 - 72h
Compound 11PF_6 L929 - 24h
Compound 11PF_6 MCF-7 - 24h
Compound 11PF_6 MDA-MB-231 - 24h
Compound 11PF_6 L929 - 72h
Compound 11PF_6 MCF-7 - 72h
Compound 11PF_6 MDA-MB-231 - 72h
% Inibition
[11NO_3] (μM)
[11PF_6] (μM)

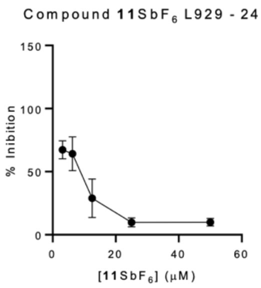
Compound 11SbF6 L929 - 24h
% Inibition
[11SbF6] (μM)

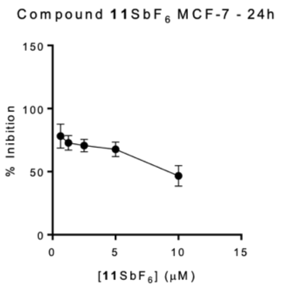
Compound 11SbF6 MCF-7 - 24h
% Inibition
[11SbF6] (μM)

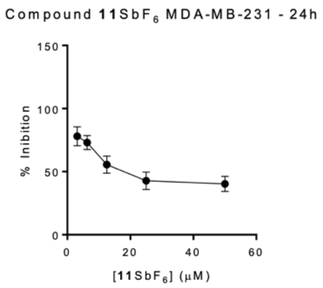
Compound 11SbF6 MDA-MB-231 - 24h
% Inibition
[11SbF6] (μM)

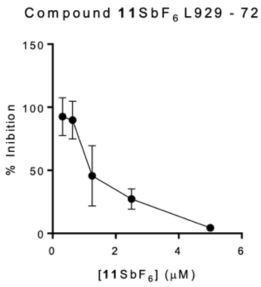
Compound 11SbF6 L929 - 72h
% Inibition
[11SbF6] (μM)

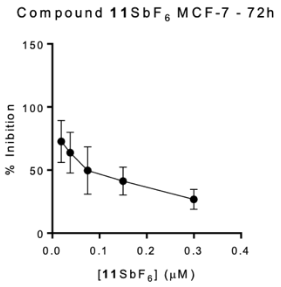
Compound 11SbF6 MCF-7 - 72h
% Inibition
[11SbF6] (μM)

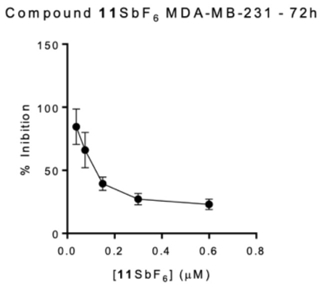
Compound 11SbF6 MDA-MB-231 - 72h
% Inibition
[11SbF6] (μM)

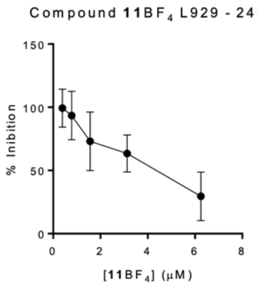
Compound 11BF4 L929 - 24h
% Inibition
[11BF4] (μM)

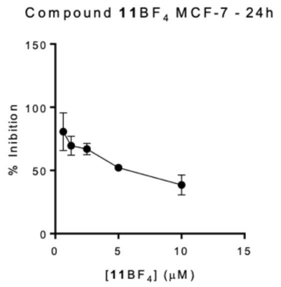
Compound 11BF4 MCF-7 - 24h
% Inibition
[11BF4] (μM)

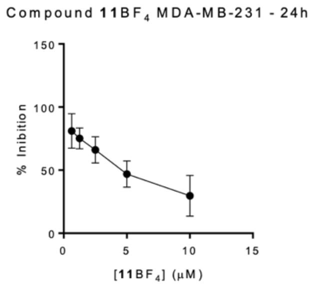
Compound 11BF4 MDA-MB-231 - 24h
% Inibition
[11BF4] (μM)

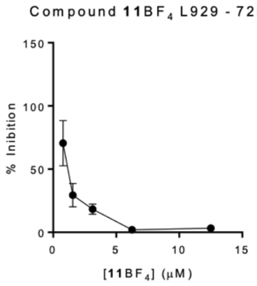
Compound 11BF4 L929 - 72h
% Inibition
[11BF4] (μM)

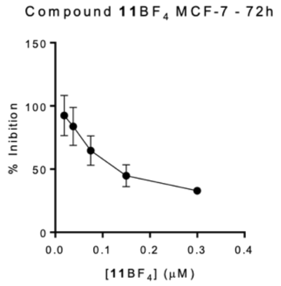
Compound 11BF4 MCF-7 - 72h
% Inibition
[11BF4] (μM)

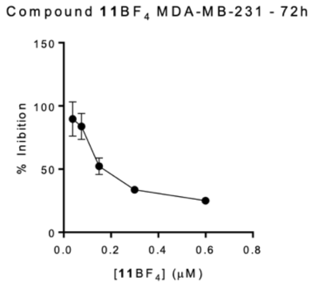
Compound 11BF4 MDA-MB-231 - 72h
% Inibition
[11BF4] (μM)

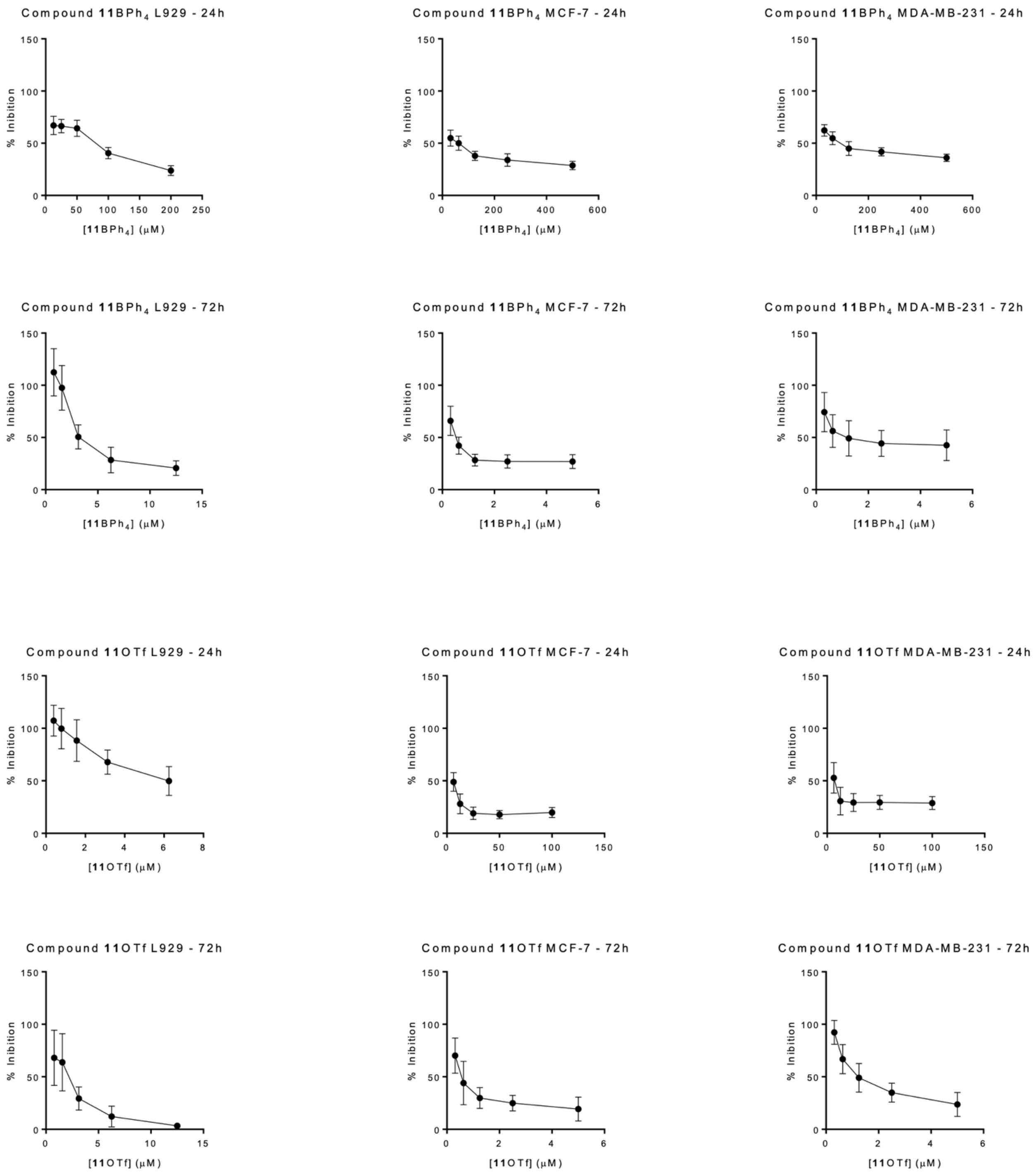

Figure S15. *Dose-response curves of **10** and **11**CA over an exposure of 24 h and 72 h.*

Table S5 *– In-vitro antiproliferative activities of compounds **1** − **11**CA against breast cancer (MCF-7 and MDA-MB-231), and non-tumoral adherent mouse fibroblast connective tissue (L929) cell lines after 24 h of exposure. See Table S2 for the full statistical analysis. *The SI (selectivity index) was calculated as IC_{50} non-cancerous L929 divided by the average of the IC_{50} of the breast cancers cell lines.*

Compound	IC_{50} (μM) (average ± standard deviation) 24 h			SI*
	MCF-7	MDA-MB-231	L929	
**1**	275 ± 12	236 ± 7	511 ± 31	2.0
**2**	403 ± 9	286 ± 7	537 ± 29	1.6
**3**	463 ± 19	357 ± 7	749 ± 45	1.8
**4**	474 ± 19	384 ± 16	723 ± 29	1.7
**5**	526 ± 19	497 ± 12	612 ± 30	1.2
**6**	376 ± 15	388 ± 12	670 ± 60	1.8
**7**	407 ± 17	513 ± 16	570 ± 18	1.2
**8**	213 ± 8	284 ± 7	572 ± 33	2.3
**9**	304 ± 19	164 ± 2	544 ± 12	2.3
**10**	128 ± 30	113 ± 14	74 ± 5	0.6
**11**Cl	4.0 ± 0.3	5.5 ± 0.6	5.3 ± 0.7	1.1
**11**NO_3	5.9 ± 0.5	4.6 ± 0.3	3.7 ± 0.4	0.7
**11**BF_4	5.4 ± 0.6	4.3 ± 0.4	3.8 ± 0.4	0.8
**11**OTf	4 ± 1	4 ± 2	5.8 ± 0.7	1.5
**11**PF_6	3.0 ± 0.3	5.0 ± 0.1	10 ± 1	2.5
**11**SbF_6	11 ± 2	21 ± 2	7.0 ± 0.4	0.4
**11**BPh_4	51 ± 5	100 ± 8	59 ± 5	0.8

Table S6 *– Statistical significance of the IC_{50} values between L929, MCF-7, and MDA-MB-231 cell lines at 24 h and 72 h.*

Compounds	Statistical significance at 24 h			Statistical significance 72 h		
	L929 / MCF-7	L929 / MDA-MB-231	MCF-7 / MDA-MB-231	L929 / MCF-7	L929 / MDA-MB-231	MCF-7 / MDA-MB-231
**1**	****	****	****	****	***	****
**2**	****	****	****	****	****	∅
**3**	****	****	****	****	****	****
**4**	****	****	****	****	****	∅
**5**	****	**	****	****	****	****
**6**	****	∅	****	****	****	****
**7**	****	****	****	****	****	****
**8**	****	****	****	****	****	∅
**9**	****	****	****	****	****	****

**10**	****	∅	****	****	∅	****
**11**Cl	∅	∅	∅	****	∅	****
**11**NO_3	∅	∅	∅	∅	∅	∅
**11**BF_4	∅	∅	∅	****	∅	****
**11**OTf	∅	∅	∅	****	****	****
**11**PF_6	****	∅	****	∅	∅	∅
**11**SbF_6	***	****	****	****	∅	****
**11**BPh_4	****	****	****	****	****	****

∅, no statistical difference; *, P<0.05; **, P<0.01; ***, P<0.001; ****, P<0.0001

Table S7. *IC_{50} values of the sodium counteranion salts tested in the selected cell lines over an exposure of 24 h and 72 h.*

**Compounds**	**IC_{50} in L929 cell line Mean ± SD***		**IC_{50} in MCF-7 cell line Mean ± SD***		**IC_{50} in MDA-MB-231 cell line Mean ± SD***	
	**24 h**	**72h**	**24 h**	**72h**	**24 h**	**72h**
**NaCl**	782 ± 55	274 ± 31	> 2000	280 ± 33	> 2000	398 ± 38
**$NaNO_3$**	> 1000	331 ± 57	> 2000	262 ± 38	> 2000	334 ± 23
**$NaBF_4$**	923 ± 77	386 ± 49	> 2000	343 ± 39	> 2000	347 ± 19
**NaOTf**	> 1000	159 ± 20	> 2000	279 ± 30	> 2000	354 ± 27
**$NaPF_6$**	759 ± 77	297 ± 24	> 2000	311 ± 27	> 1000	329 ± 17
**$NaSbF_6$**	693 ± 53	180 ± 22	> 1000	234 ± 24	689 ± 61	236 ± 13
**$NaBPh_4$**	186 ± 11	55 ± 4	217 ± 44	102 ± 14	441 ± 61	149 ± 13

5. Apoptosis effect of compounds tested in breast cancer cell lines

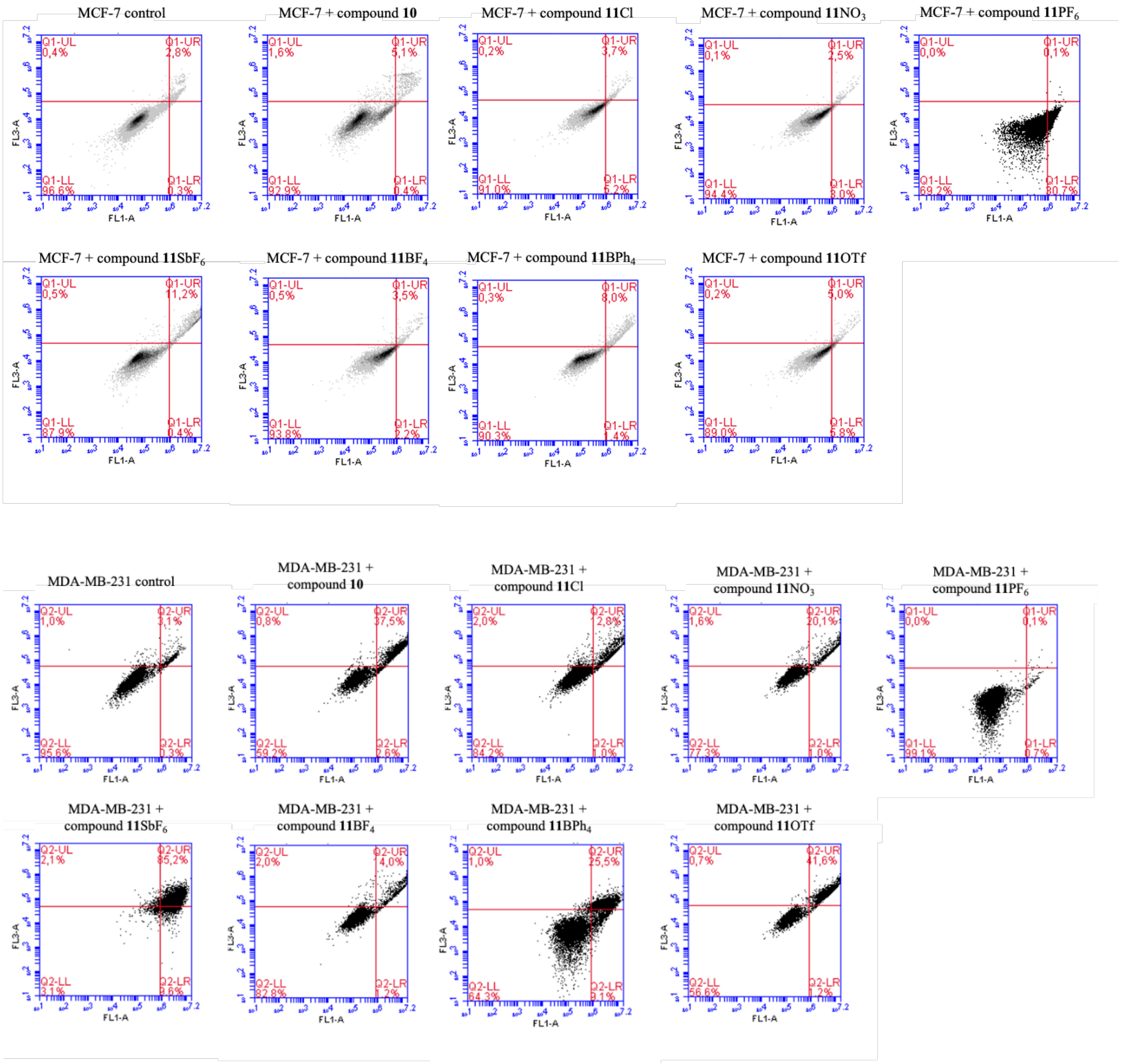

Figure S16. *Apoptosis profiles of* ***11****CA in MCF-7 and MDA-MB-231 cell lines after 24 h. Dot-plot graphs illustrated the viable cells (the lower left quadrant), early-phase apoptotic cells (the lower right quadrant), late-phase apoptotic or dead cells (the upper right quadrant), and the necrotic cells (the upper left quadrant).*

6. Reactivity with model biomolecules

The interaction between **11**Cl and different model biomolecules (*aminoacids*: glutamic acid, histidine and cysteine; *nucleobase*, dGMP) was studied by ^{1}H and ^{31}P NMR spectroscopy over 72 h in a D_2O:d_6-DMSO mixture (90:10) at 37 °C. The complex and the different nucleophiles in a 1:3 ratio were fully dissolved in d^6-DMSO, diluted with D_2O to reach 10% DMSO in D_2O (final concentration, 1 mM) and, finally, the pD was adjusted to 7.2 ± 0.1. The interaction with Cys was studied 20% d_4-methanol in D_2O, to avoid the oxidation of the thiol caused by d_6-DMSO [17] Furthermore, the interaction was monitored by ESI-MS after 72 h. **11**Cl and the model biomolecules (1:3) were dissolved in DMSO and the solution was diluted with MilliQ water to reach 0.1% DMSO in water (final concentration, 10 µM). Aliquots were taken and injected into the spectrometer without any further sample dilution or preparation.

The catalytic activity of **11**Cl in transfer hydrogenation (NAD^+/NADH) and glutathione oxidation reactions (GSH/GSSG) at 37 °C was monitored by ^{1}H and ^{31}P NMR spectroscopy over 8 h. Complex **11**Cl was dissolved in d_4-methanol/D_2O (5:1 *v*/*v*, 1.4 mM). In a typical experiment, 3 mL of a solution containing complex **11**Cl (0.44 mM) and either NAD^+ (0.88 mM, 2 eq.) and sodium formate (11.02 mM, 25 eq.), for transfer hydrogenation; or reduced glutathione (1.32 mM, 3 eq.), for oxidation, were added to a 10-mm NMR tube. The pD was adjusted to 7.2 ± 0.1. ^{1}H NMR spectra and ^{31}P NMR spectra were recorded at 310 K every 300 s for 8 h.

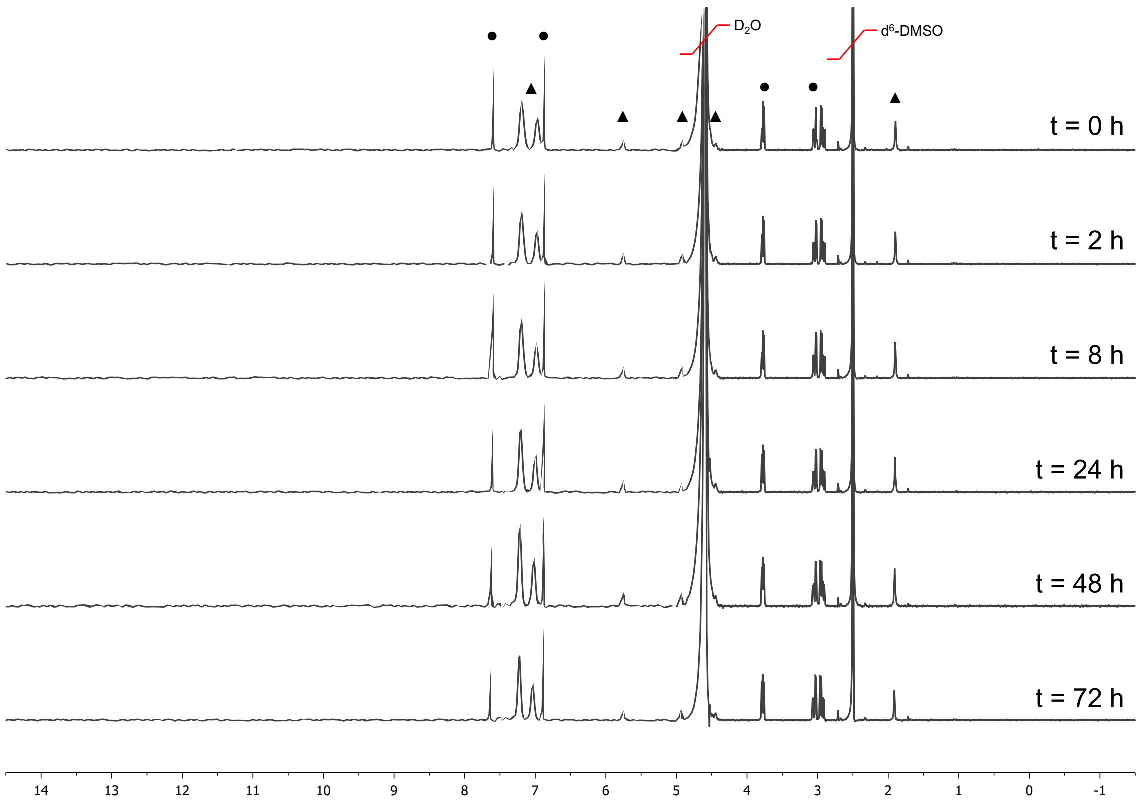

Figure S17. *Interaction of **11**Cl (▲) with His (●) in a D_2O:d_6-DMSO mixture (90:10) monitored by ^1H NMR.*

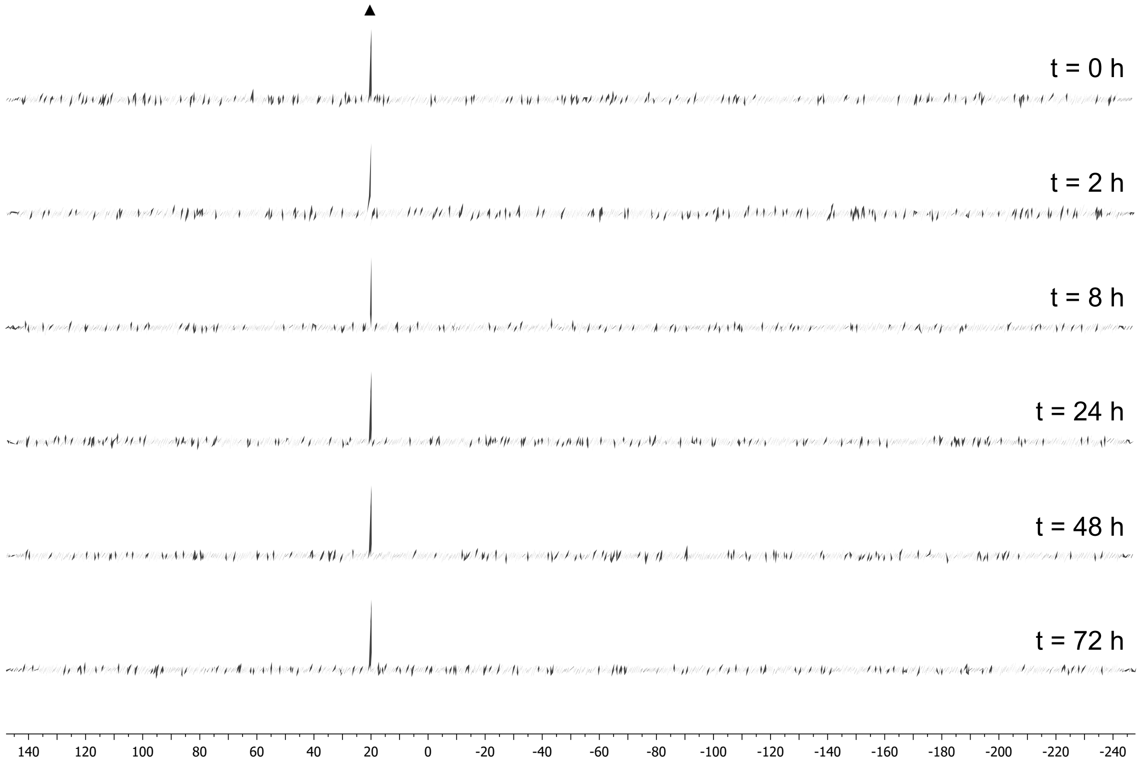

Figure S18. *Interaction of **11**Cl (▲) with His (●) in a D_2O:d_6-DMSO mixture (90:10) monitored by ^{31}P NMR.*

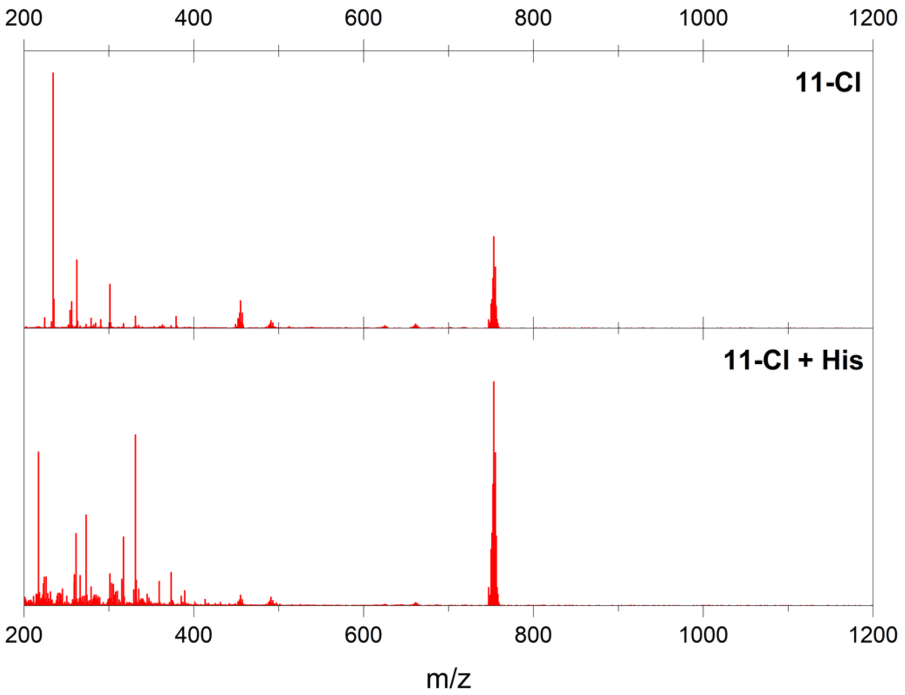

Figure S19. *ESI-MS spectra of **11**Cl and of its interaction with His in milliQ water with 0.1% DMSO after 72 h.*

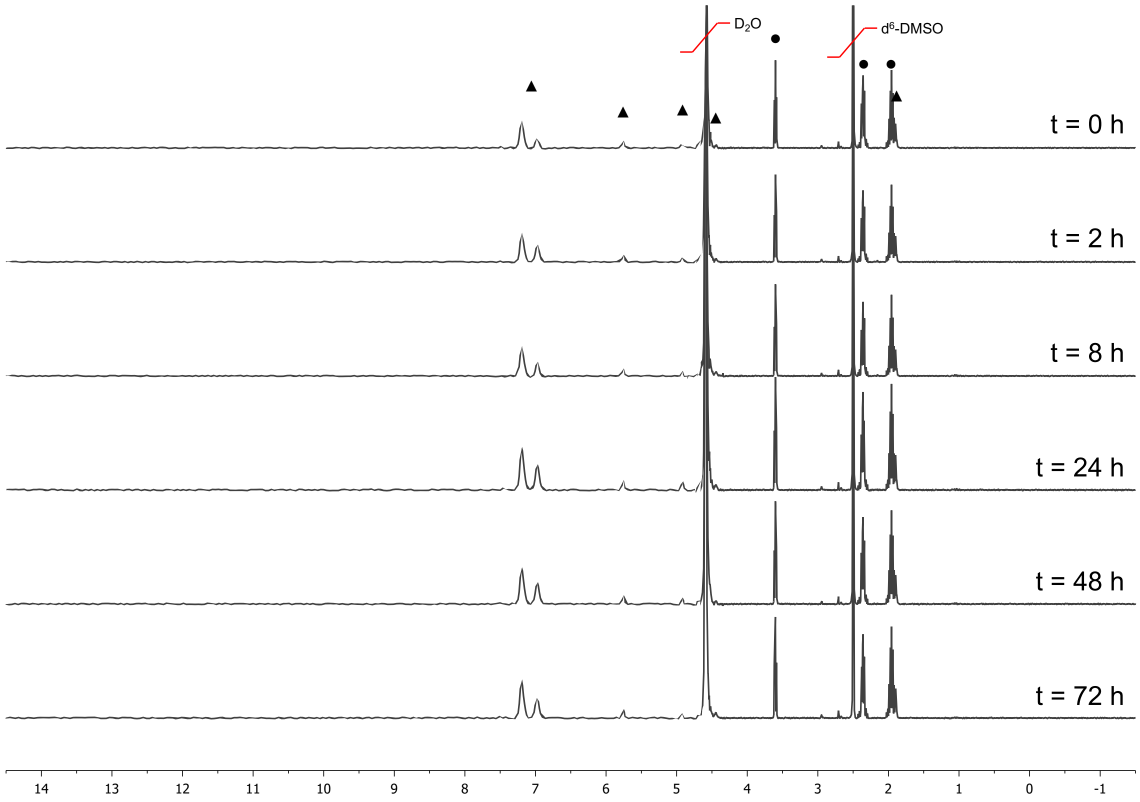

Figure S20. *Interaction of **11**Cl (▲) with Glu (●) in a D_2O:d_6-DMSO mixture (90:10) monitored by ^1H NMR.*

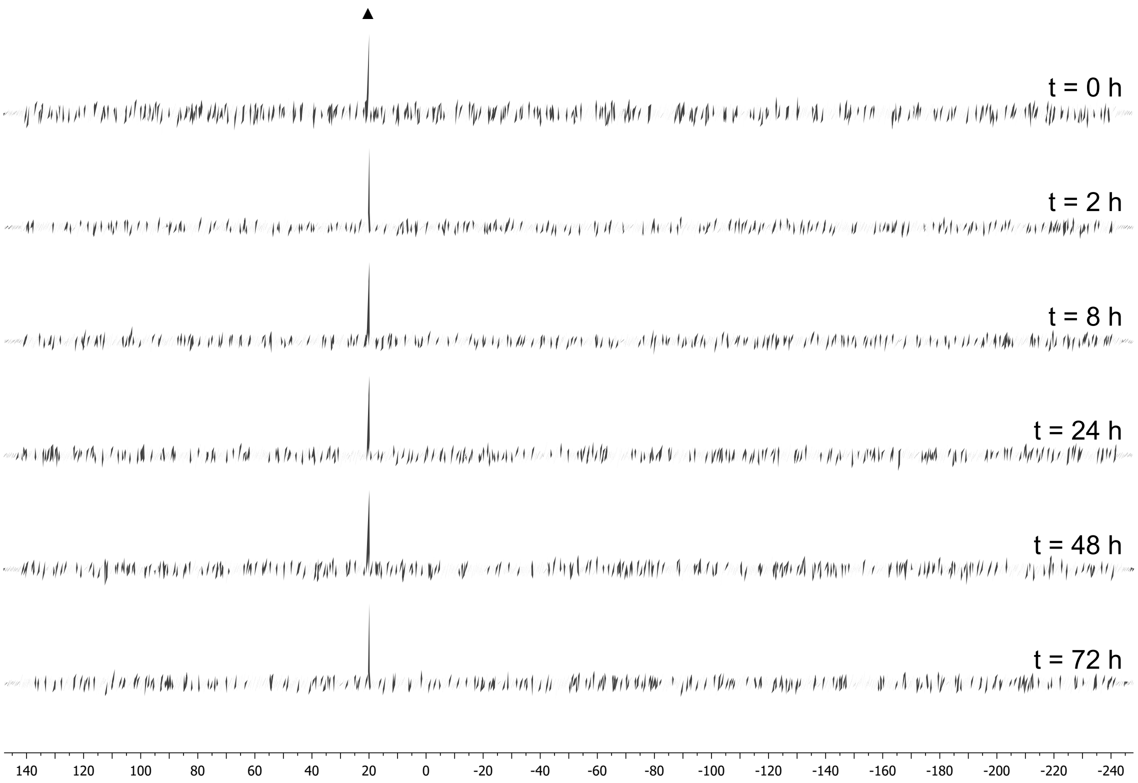

Figure S21. *Interaction of **11**Cl (▲) with Glu (●) in a D_2O:d_6-DMSO mixture (90:10) monitored by ^{31}P NMR.*

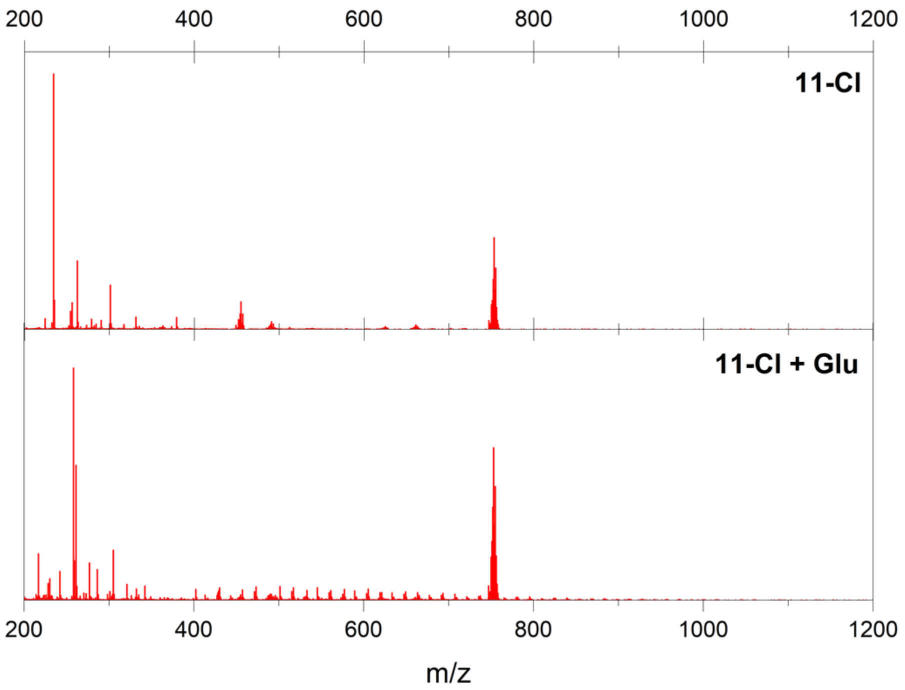

Figure S22. *ESI-MS spectra of **11**Cl and of its interaction with Glu in milliQ water with 0.1% DMSO after 72 h.*

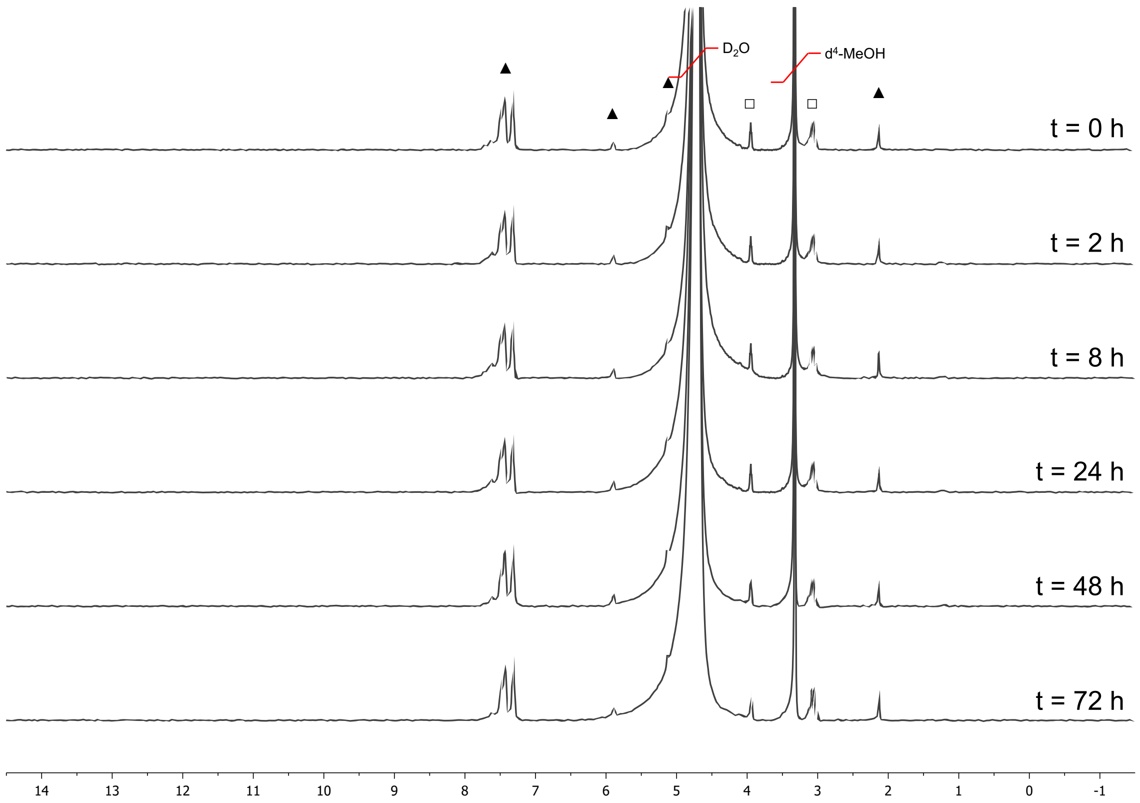

Figure S23. *Interaction of **11**Cl (▲) with Cys (●) in a D_2O:d_4-methanol mixture (80:20) monitored by ^1H NMR.*

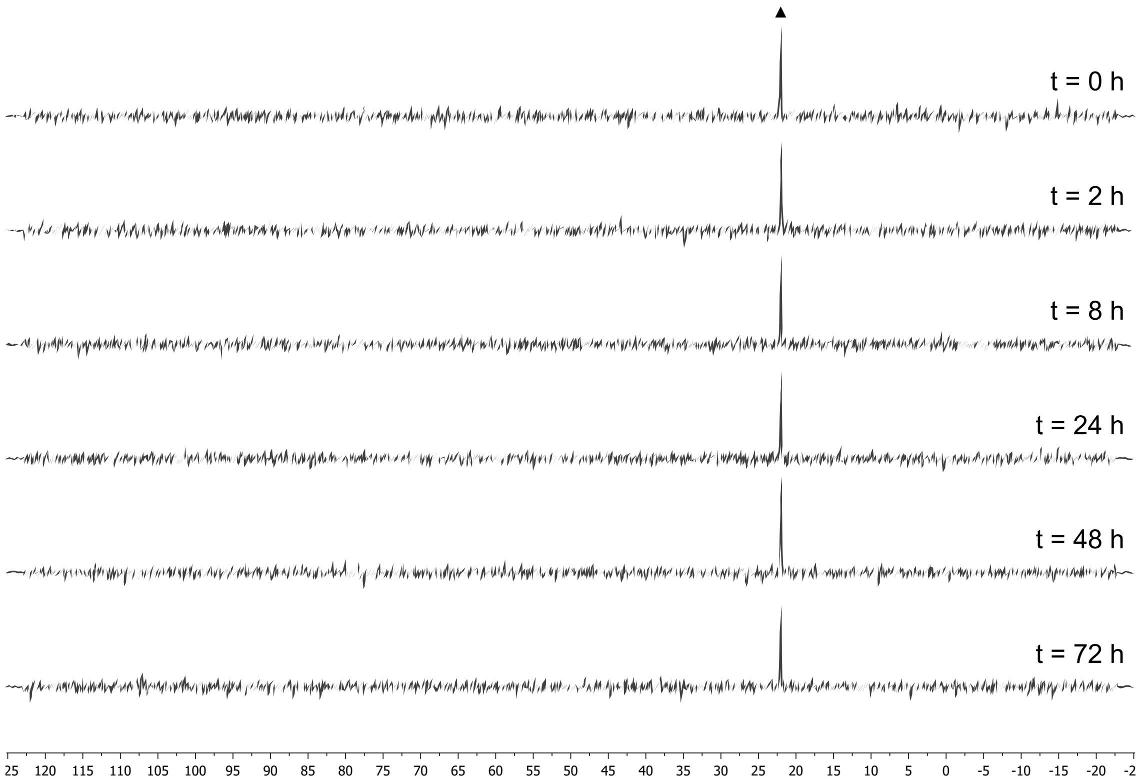

Figure S24. *Interaction of **11**Cl (▲) with Cys (●) in a D_2O:d_4-methanol mixture (80:20) monitored by ^{31}P NMR.*

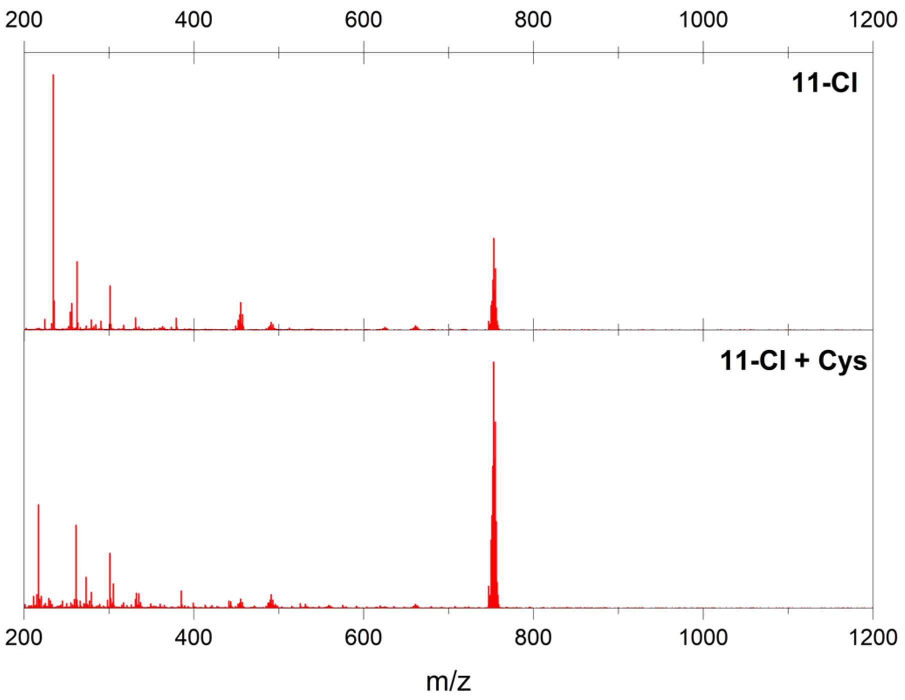

Figure S25. *ESI-MS spectra of **11**Cl and of its interaction with Cys in milliQ water with 0.1% DMSO after 72 h.*

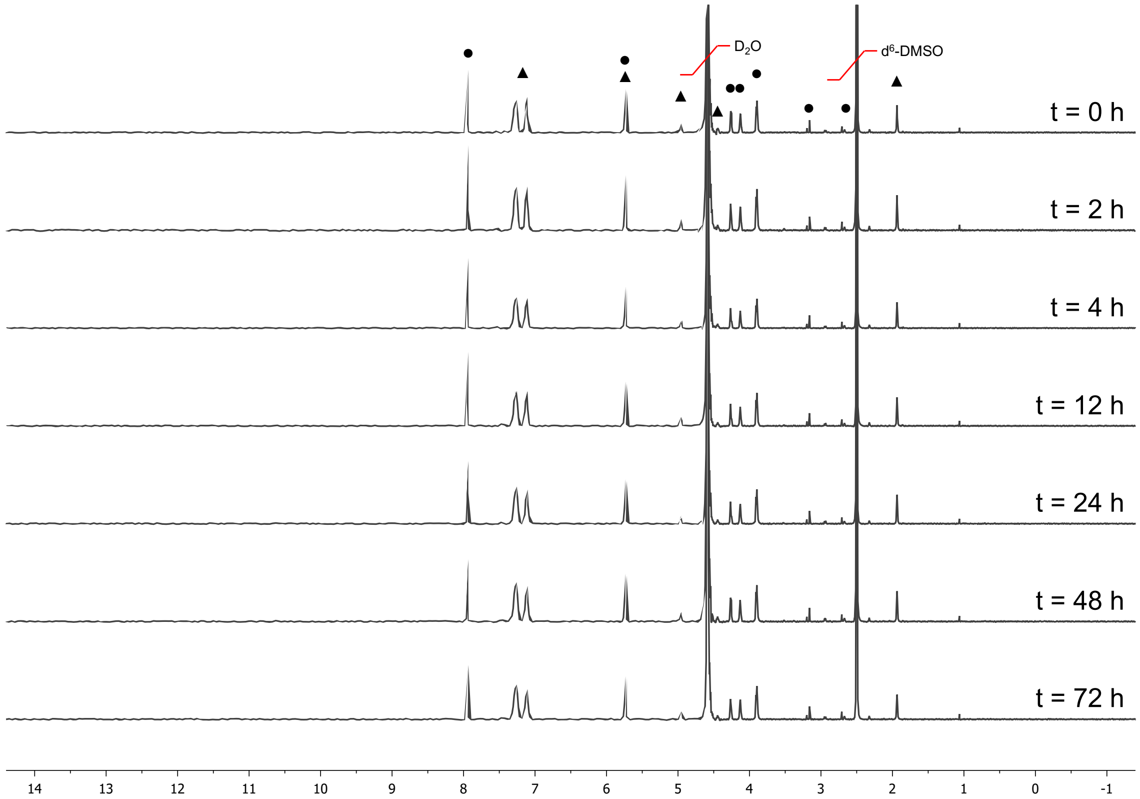

Figure S26. *Interaction of **11**Cl (▲) with dGMP (●) in a D_2O:d_6-DMSO mixture (90:10) monitored by ^1H NMR.*

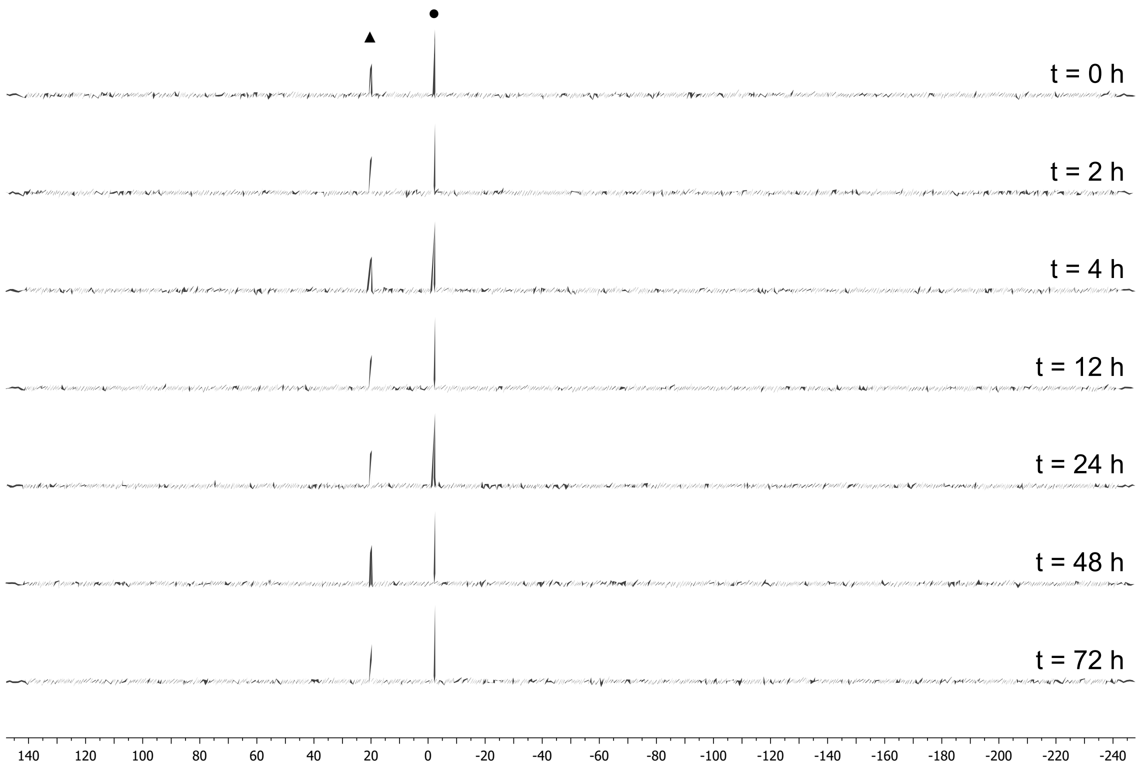

Figure 27. *Interaction of **11**Cl (▲) with dGMP (●) in a D_2O:d_6-DMSO mixture (90:10) monitored by ^{31}P NMR.*

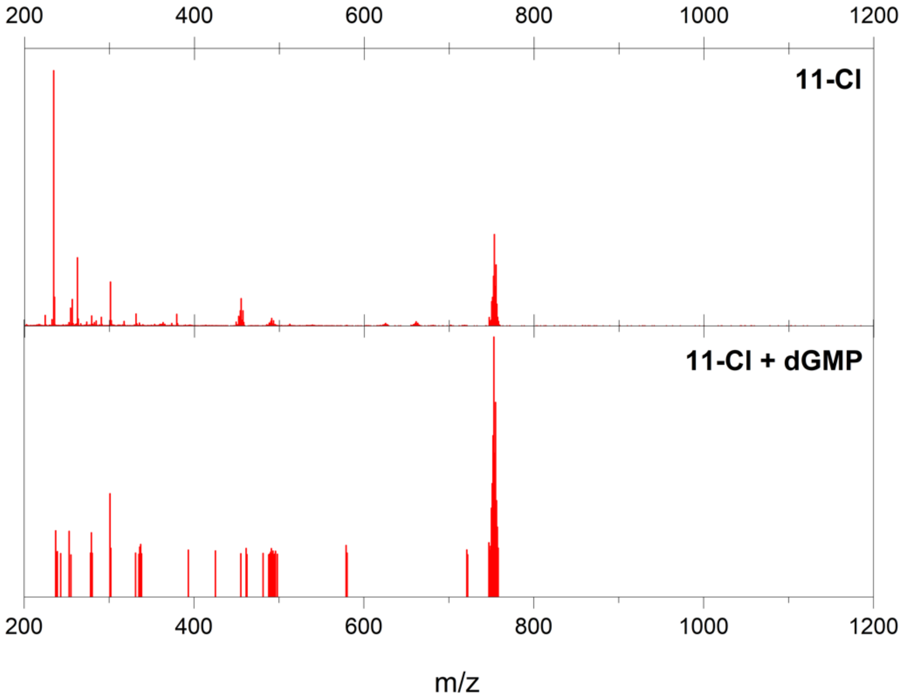

Figure S28. *ESI-MS spectra of **11**Cl and of its interaction with dGMP (1:3) in milliQ water with 0.1% DMSO after 72 h.*

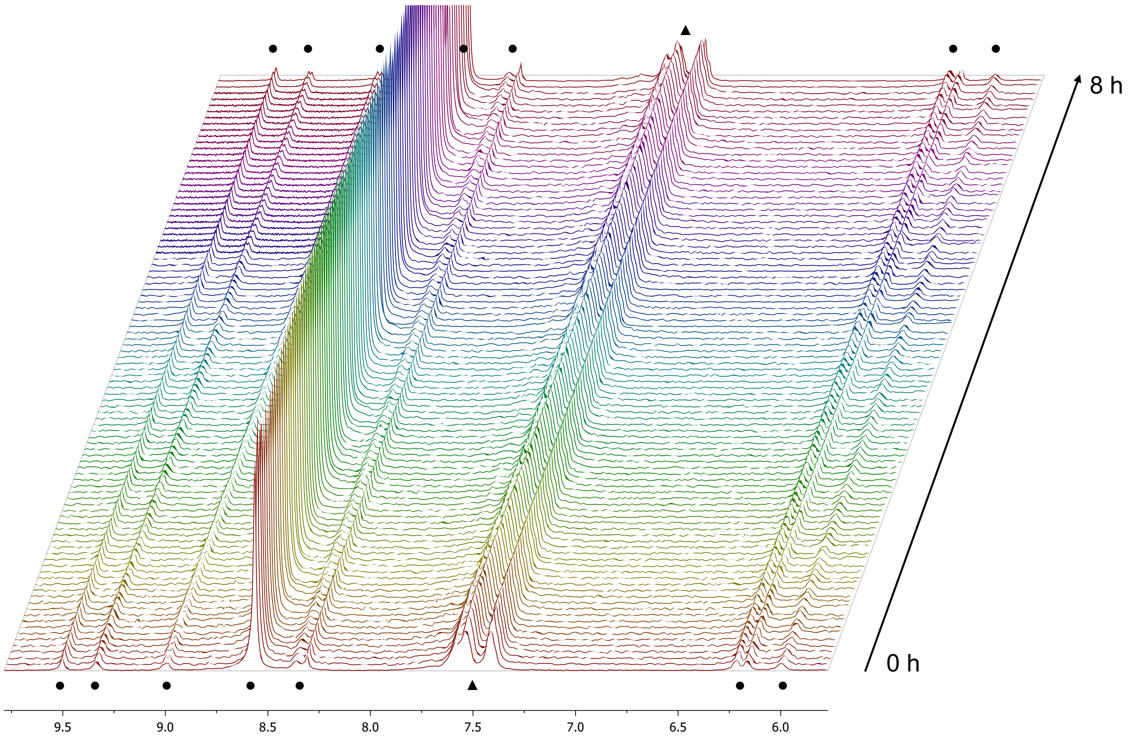

Figure S29. *^{1}H NMR monitoring of the NAD$^+$/NADH (●) transfer hydrogenation by* ***11****Cl (▲).*

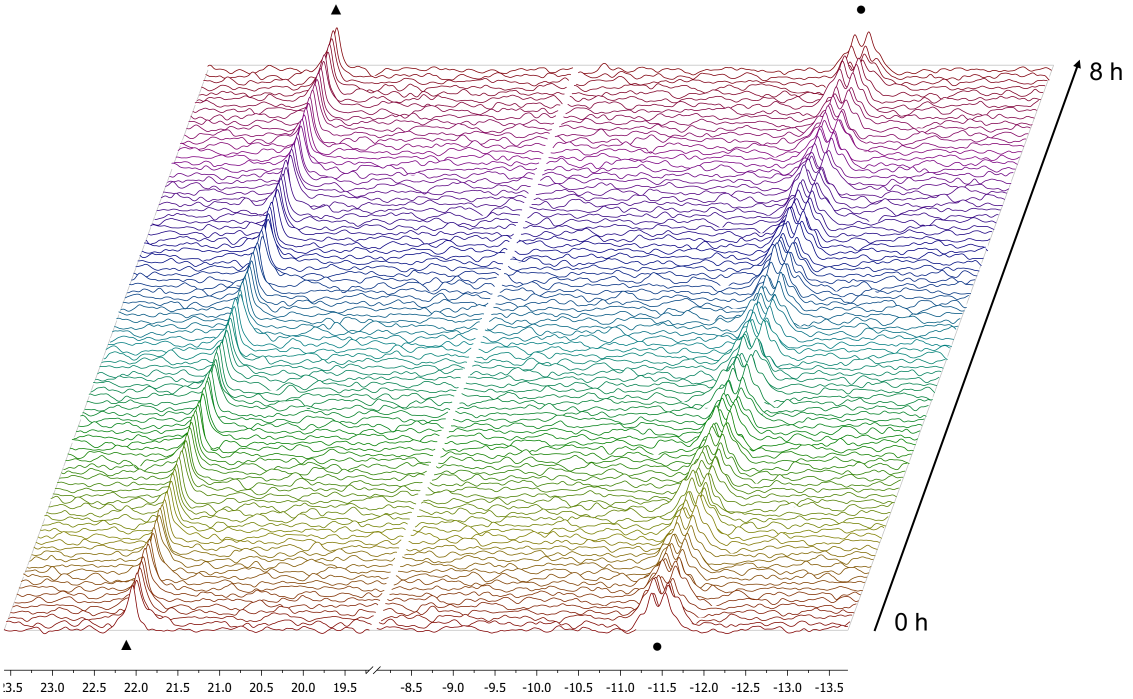

Figure S30. *^{31}P NMR monitoring of the NAD$^+$/NADH (●) transfer hydrogenation by* ***11****Cl (▲).*

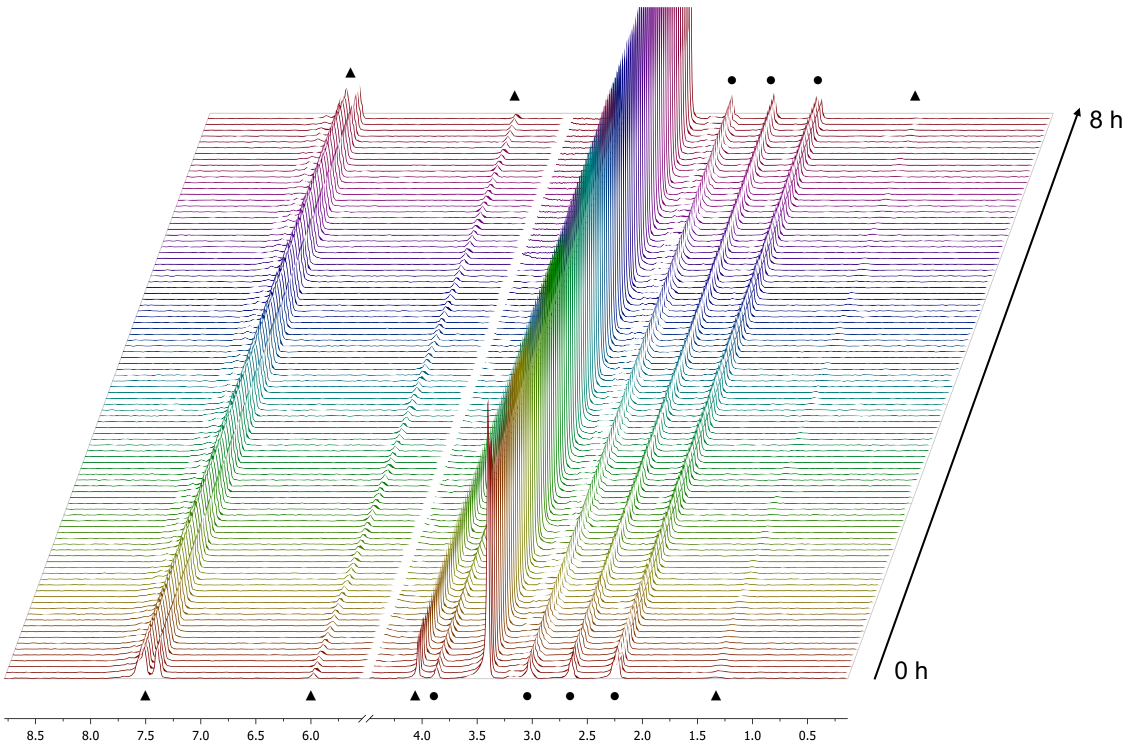

Figure S31. *^{1}H NMR monitoring of the GSH (●) oxidation by **11**Cl (▲).*

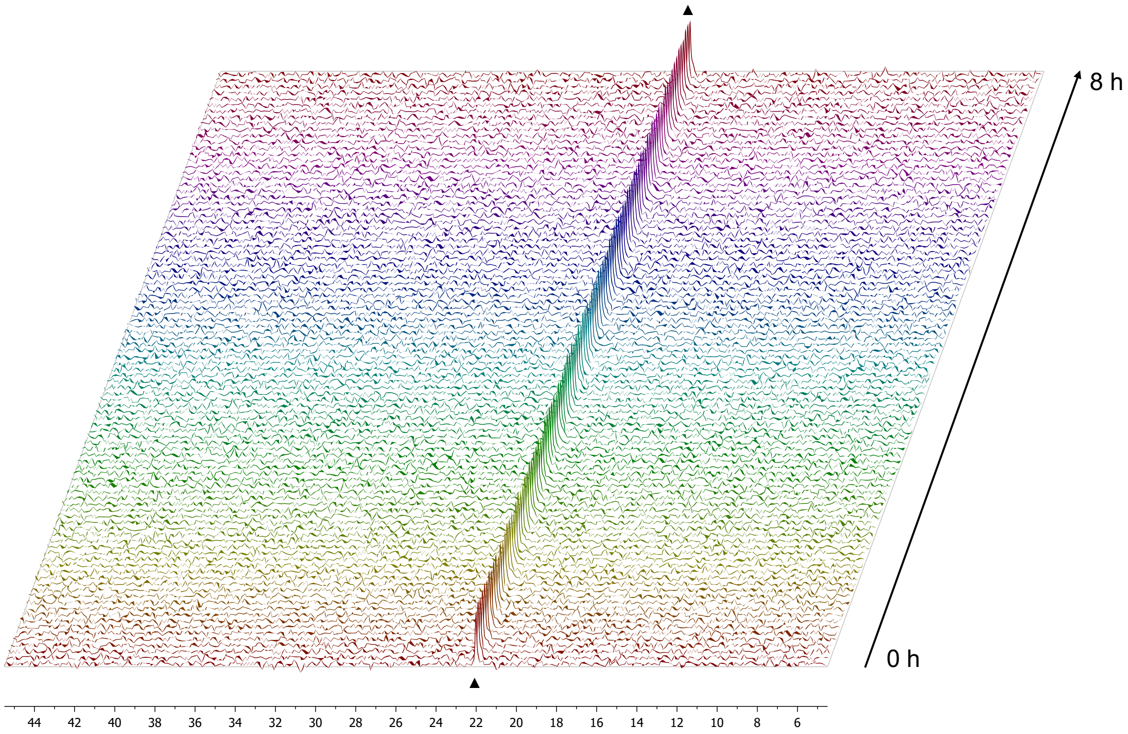

Figure S32. *^{31}P NMR monitoring of the GSH (●) oxidation by **11**Cl (▲).*

Table S8. *Protein quenching constant (K_{sv}), bimolecular quenching rate (k_q), binding constant (K_b), and number of binding sites (n) for the interaction of **11**Cl with BSA.*

**Protein**	K_{SV} (10^4 M^{-1})	k_q (10^{12} M^{-1})	K_b (10^5 M^{-1})	***n***
BSA	1.13 ± 0.08	1.88 ± 0.14	5.6 ± 0.3	1.4

Table S9. *Protein quenching constant (K_{sv}), bimolecular quenching rate (k_q), c_{50}, and apparent binding constant (K_{app}) for the interaction between **11**Cl and ctDNA from the DAPI and MG competitive binding assay.*

**Probe**	K_{SV} (10^3 M^{-1})	k_q (10^{12} M^{-1})	C_{50} (10^{-4} M)	K_{app} (10^5 M^{-1})
DAPI	8.8 ± 0.6	3.3 ± 0.3	1.1	4.4 ± 0.3
MG	4.8 ± 0.7	0.48 ± 0.07	2.1	0.43 ± 0.04

7. Circular dichroism

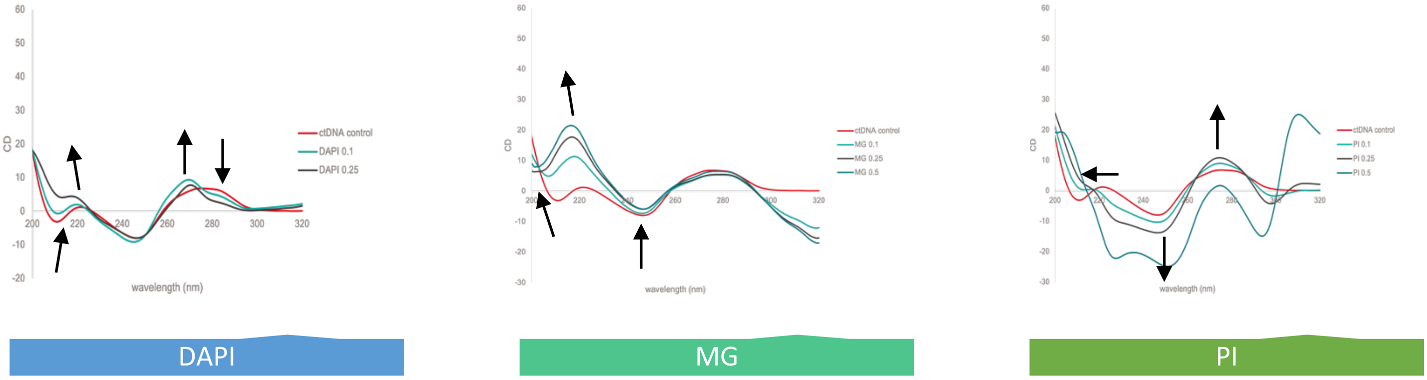

Figure S33. *Circular dichroism spectra of dsDNA incubated with DAPI (left), MG (center) and PI (right).*

Table S10. *Summary of the CD band changes on dsDNA induced by DAPI, MG, and PI.*

Probe	**210 nm**		**220 nm** *Hydrogen bonding*		**246 nm** *Helicity*		**268–280 nm** *Base-pair stacking interactions*	
DAPI *Minor groove*	Hypochromic, Δ↓	Red shift, λ↑	Hyperchromic, Δ↑	Blue shift, λ↓	Unchanged	Unchanged	*268 nm –* Hyperchromic, Δ↑ *280 nm –* Hypochromic, Δ↓	Unchanged
MG *Major groove*	Hypochromic, Δ↓	Blue shift, λ↓	Hyperchromic, Δ↑	Blue shift, λ↓	Hypochromic, Δ↓	Unchanged	Unchanged	Unchanged
PI *Intercalation*			Unchanged	Blue shift, λ↓	Hyperchromic, Δ↑	Unchanged	Hyperchromic, Δ↑	Unchanged

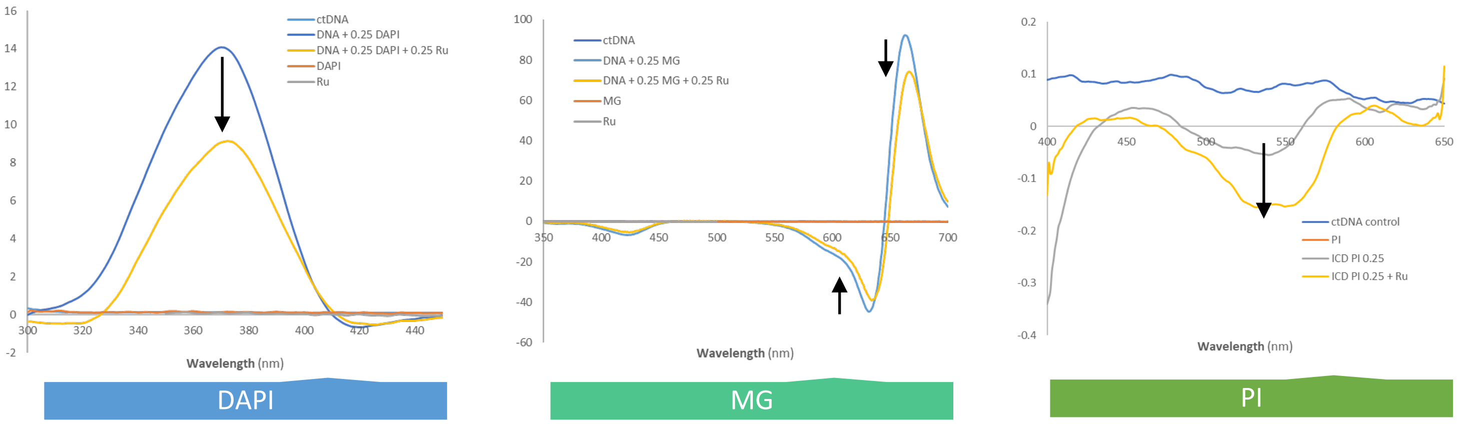

Figure S34. *Induced circular dichroism spectra of dsDNA incubated with DAPI (left), MG (center) and PI (right).*

8. Tandem mass spectrometry of 11Cl and DNA oligomers

11Cl was incubated with 14-mer ssDNA 5’−3’ (ATACATGGTACATA) and 6-mer dsDNA 5’−3’ (AGGCAG) (10 µM) in a 3:1 ratio at 37 °C in MilliQ (pH 7). After 24 and 72 h, respectively, the solution is centrifuged with an Amicon® Ultra 0.5 mL centrifugal filter 3kDa MWCO to remove the unreacted metal complex and excess salts. Mass spectra were recorded on an LTQ Orbitrap Elite FTMS instrument (Thermo Scientific) operating in negative mode with a HESI-II probe in an Ion Max ion Source interface. The ionization voltage was set at −1.4 kV and the ion transfer capillary temperature at 120 °C. For tandem MS data analysis, FTMS spectra were obtained in the 160-2000 m/z range in the reduced profile mode with a resolution set to 120k. In all spectra, 10 microscan was acquired with a maximum injection time value of 2000 ms. High-resolution mass spectra of the ruthenated oligonucleotides were analyzed using the automated tool termed Analysis of Oligonucleotide Modifications from Mass Spectra (Aom^{2}s) [18]. Aom^{2}s was used to calculate theoretical MS and MS/MS ions from oligonucleotide sequences with desired metal adducts. Subsequently, Aom^{2}s automatically matches theoretical isotopic patterns to the experimental isotopically resolved mass spectrum and generates graphic representations of the oligonucleotide as a fragment map. Aom^{2}s input parameter included the loss of protons ((H^+)−1−−10); variables groups for MS analysis included the different ruthenium adducts Ru^{2+}, $RuCl^+$, $[Ru(PPh_3)]^{2+}$, $[Ru(PPh_3)Cl]^+$, $[Ru(PPh_3)_2]^{2+}$, $[Ru(PPh_3)_2Cl]^+$, $[Ru(\eta^6\text{-toluene})(PPh_3)]^{2+}$, $[Ru(\eta^6\text{-toluene})(PPh_3)_2]^{2+}$ and $[Ru(\eta^6\text{-toluene})(PPh_3)_2Cl]^+$; ions ($K^+$ and Na^+); and modifiable charge (0 to +2). MSMS of ruthenium-containing adducts were fragmented by collision-induced dissociation (CID) in the linear ion trap using an isolation window of 8 Da, with product ion detected in the Orbitrap with a resolution set to 120 K. Variables groups for MS/MS analysis included the different ruthenium adducts Ru^{2+}, $[Ru(PPh_3)]^{2+}$, $[Ru(\eta^6\text{-toluene})]^{2+}$and $[Ru(\eta^6\text{-toluene})(PPh_3)]^{2+}$. Top and bottom zone widths were selected according to the isotope distribution of Ru (−2.5 to 7.5) and allowed experimental mass error and minimal similarity to be set to 10 ppm and 70%, respectively.

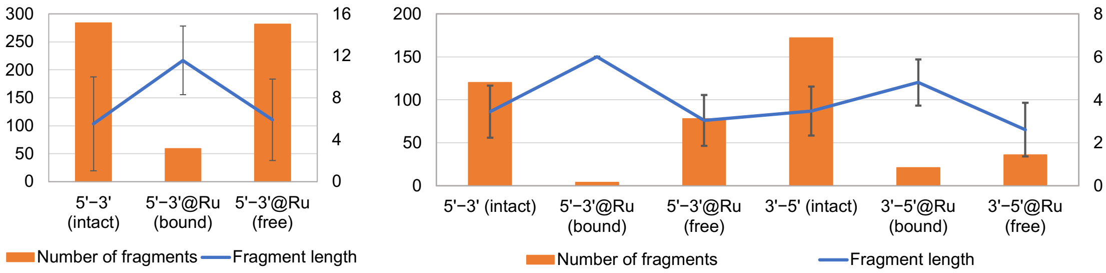

Figure S35. *MS² CID fragmentation of the isolated ruthenium-bound (A), ruthenium-free (B) and intact (C) 14-mer ssDNA ions. Fragment labels in black and gray have an average similarity above and below 90%, respectively.*

A

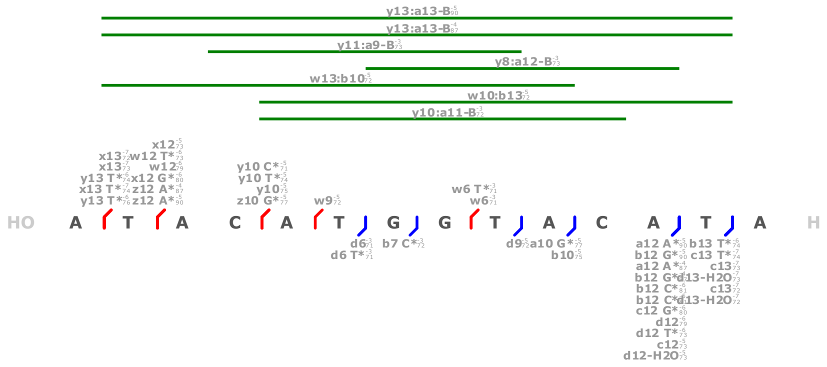

B

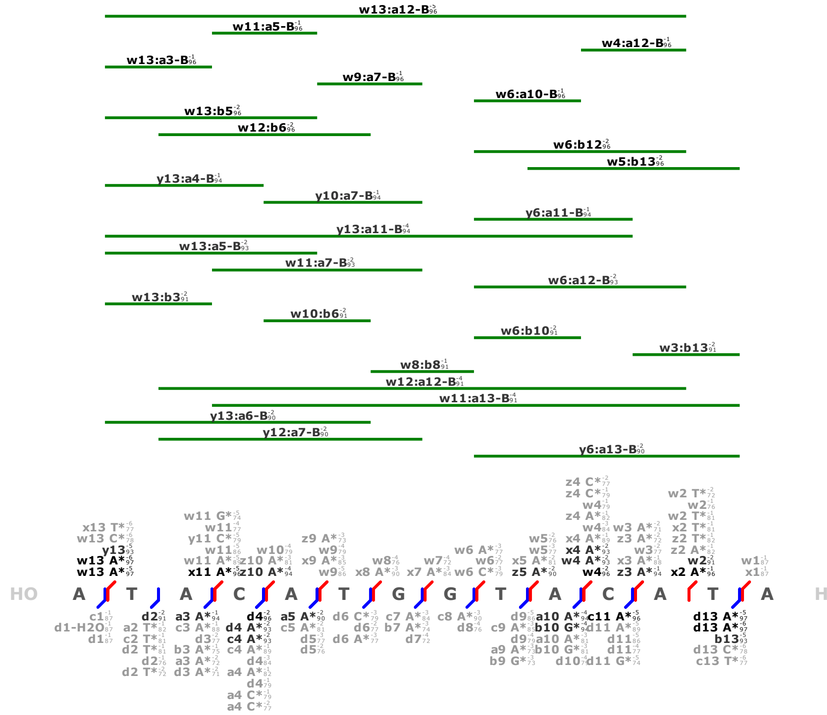

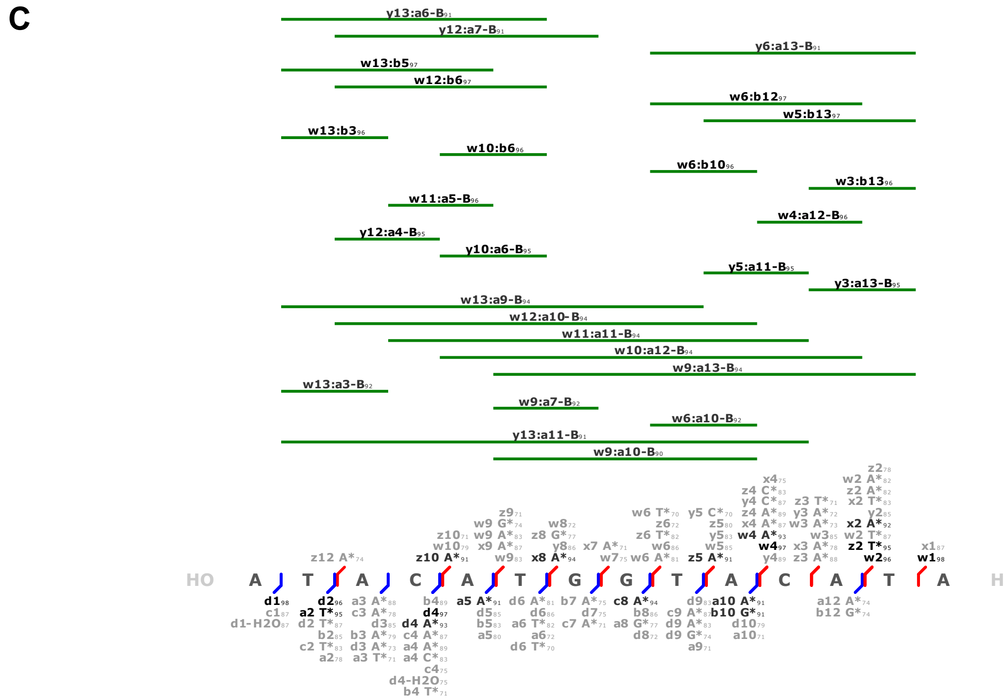

Figure S36. *MS² CID fragmentation of the isolated 14-mer ssDNA ions:ruthenium-bound (A), ruthenium-free (B) and intact oligonucleotide (C). Fragment labels in black and gray have an average similarity above and below 90%, respectively.*

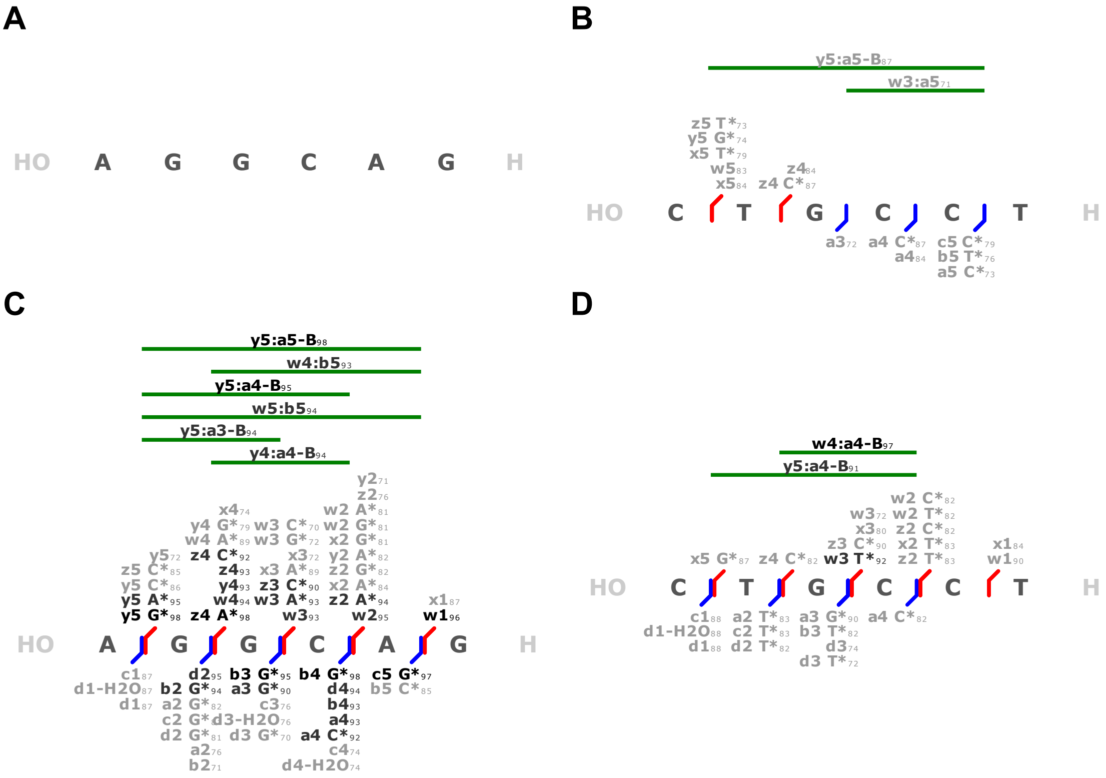

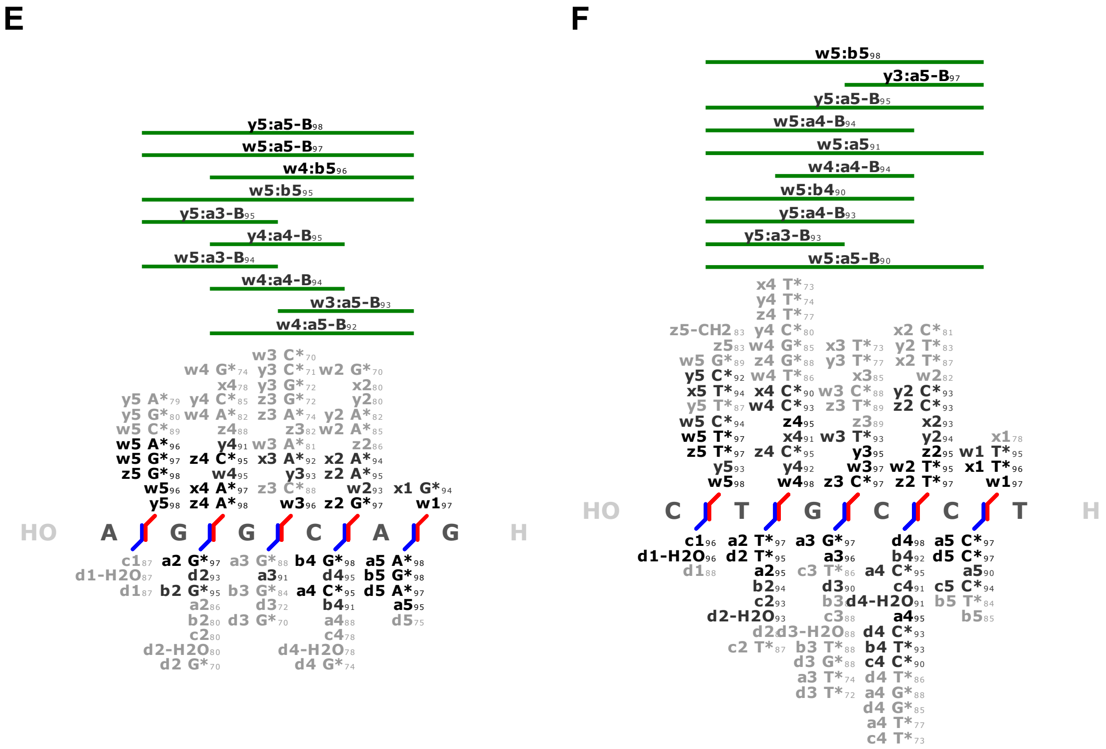

Figure S37. *MS² CID fragmentation of the isolated 6-mer dsDNA ions (left and right figures correspond to the 5’−3’ and the 3’−5’ strands, respectively): ruthenium-bound (A, B), ruthenium-free (C,D) and intact (E,F). Fragment labels in black and gray have an average similarity above and below 90%, respectively.*

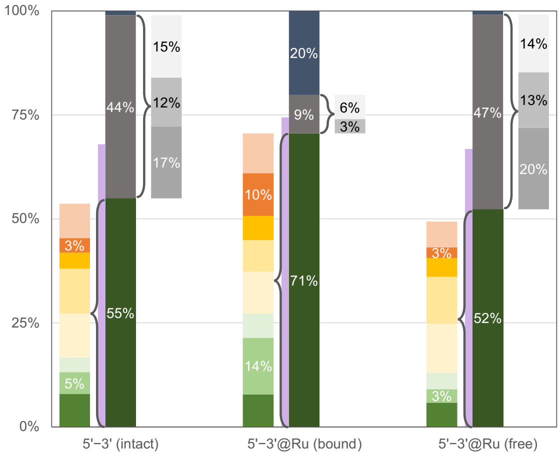

Figure S38. *Detailed analysis of the fragmentation of the intact, ruthenium-bound and ruthenium-free 14-mer oligonucleotide isolated ions: terminal fragments; internal fragments; and not fragments. Terminal fragmentation mechanisms of type a, ; b, ; c, ; d, ; w, ; x, ; y, ; and z, . Internal fragmentation mechanisms of type W/A, ; W/B, ; and Y/A, . Fragmentation mechanisms including base loss .*

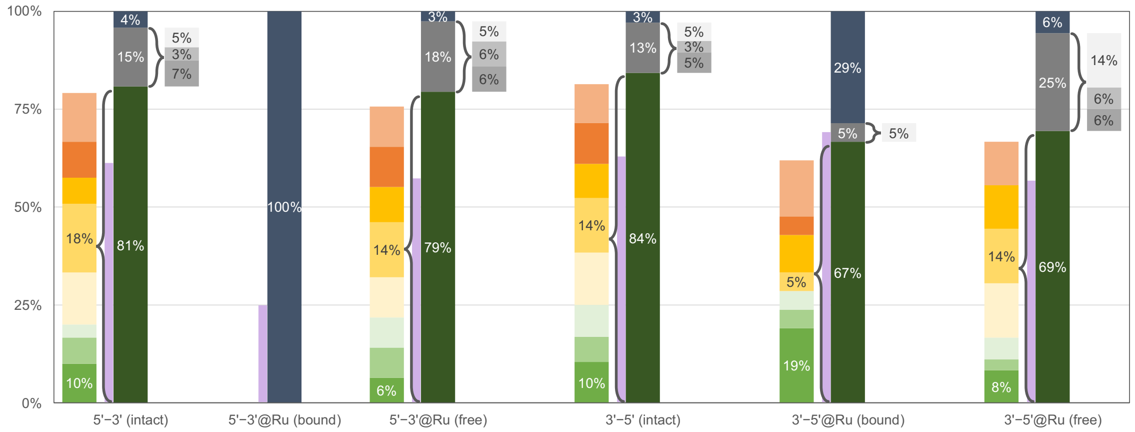

Figure S39. *Detailed analysis of the fragmentation of the intact, ruthenium-bound and ruthenium-free 6-mer oligonucleotide isolated ions: ■ terminal fragments; ■ internal fragments; and ■ not fragments. Terminal fragmentation mechanisms of type a, ■; b, ■; c, ■; d, ■; w, ■; x, ■; y, ■; and z, ■. Internal fragmentation mechanisms of type W/A, ■; W/B, ■; and Y/A, ■. Fragmentation mechanisms including base loss ■.*

9. Molecular docking

The docking files were prepared using AutoDock Tools. After removing the solvent molecules, cocrystallized ligands, and cofactors, all the hydrogens atoms were added to DNA, Gasteiger charges were calculated, and then non-polar hydrogen atoms were merged. The free torsion tree was assigned, and the ruthenium atom parameters were added. A grid box was created with 96 × 96 × 96 points and a resolution of 0.375 Å (which is roughly a quarter of the length of a carbon-carbon single bond), in order to include the entire DNA fragment. Standard AutoDock force fields were used to calculate the energies and the best conformation was optimized by applying a Lamarckian Genetic Algorithm (LGA). With an initial population size of 300 and several evaluations of 2500000, the GA was allowed to run up to 27000 generations to find one best individual. Each experiment was set to 100 GA runs. The gene mutation and crossover rate were fixed at 2% and 80%, respectively. Clustering was performed to analyze the convergence of the simulations. The resulting poses were visualized with Visual Molecular Dynamics (VMD) [19] (University of Illinois, USA) and Discovery Studio Visualizer (BIOVIA, Dassault Systèmes).

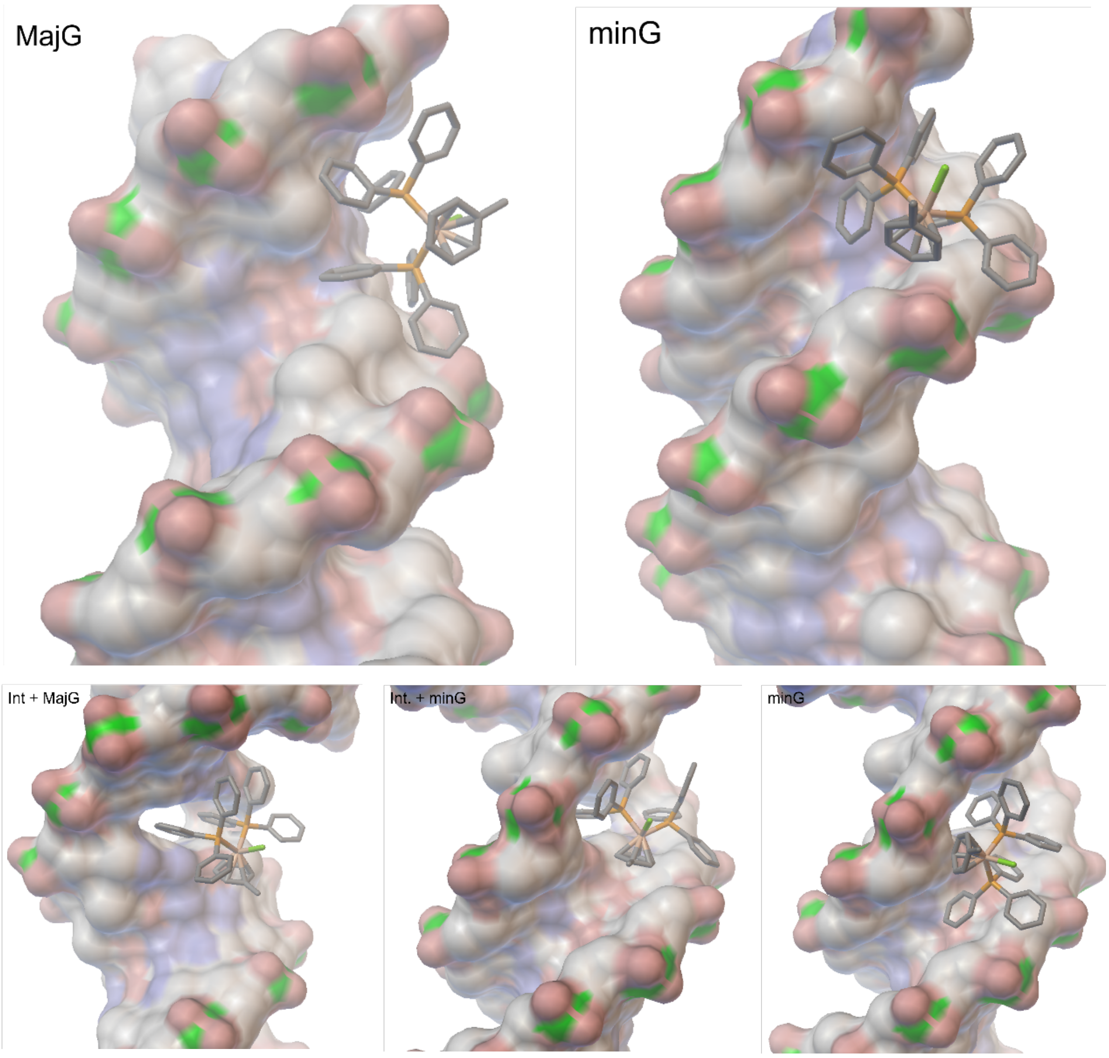

Figure S40. *Docked conformations for* ***11***$^{+}$ *interacting with 5T4W, minor groove (top); and 1G3X, intercalator (bottom).*

Table S11. *Binding energy for DAPI and MG for the different DNA structures.*

PDB structure	1BNA	5T4W	1G3X
**DAPI**	−9.49 kcal/mol	−9.61 kcal/mol	−9.95 kcal/mol
**MG**	−9.29 kcal/mol	−9.27 kcal/mol	−10.57 kcal/mol

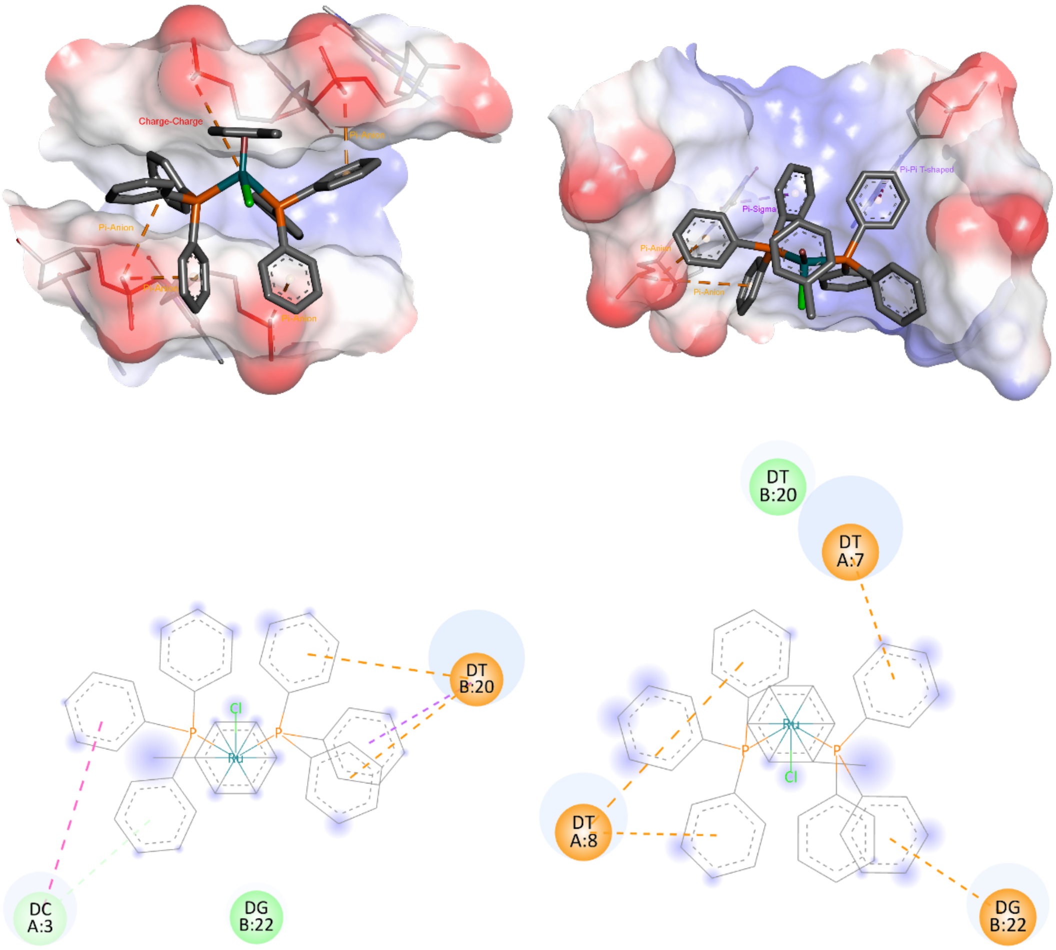

Figure S41. *3D (top) and 2D (bottom) interaction maps of the docking results of **11**$^{+}$ interacting with the minor (left) and the major (right) groove of 1BNA. Charge-charge (red), van der Waals (green), π-anion (orange), π-sigma (purple), T-shaped π-π (pink), and π-HBD (light green). Blue shading indicates solvent accessibility.*

10.NMR spectra of the synthesized compounds

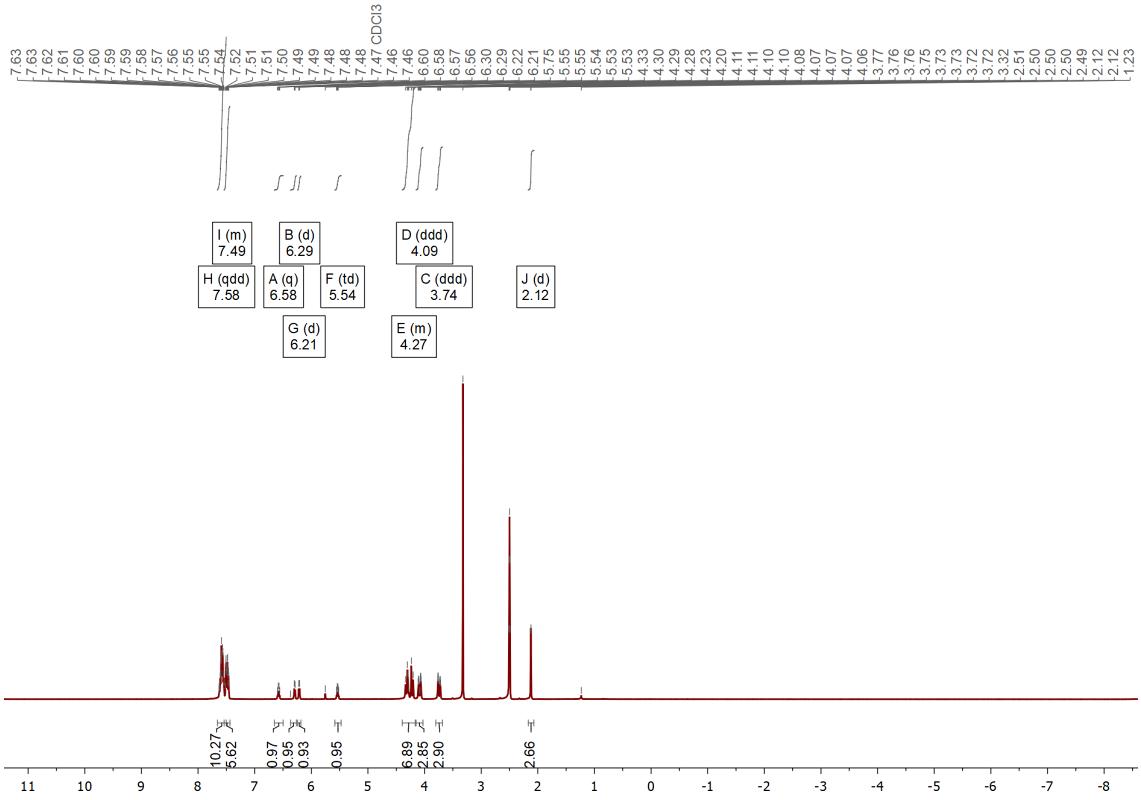

Figure S42. *^{1}H NMR spectra of **10** in DMSO-d$_6$.*

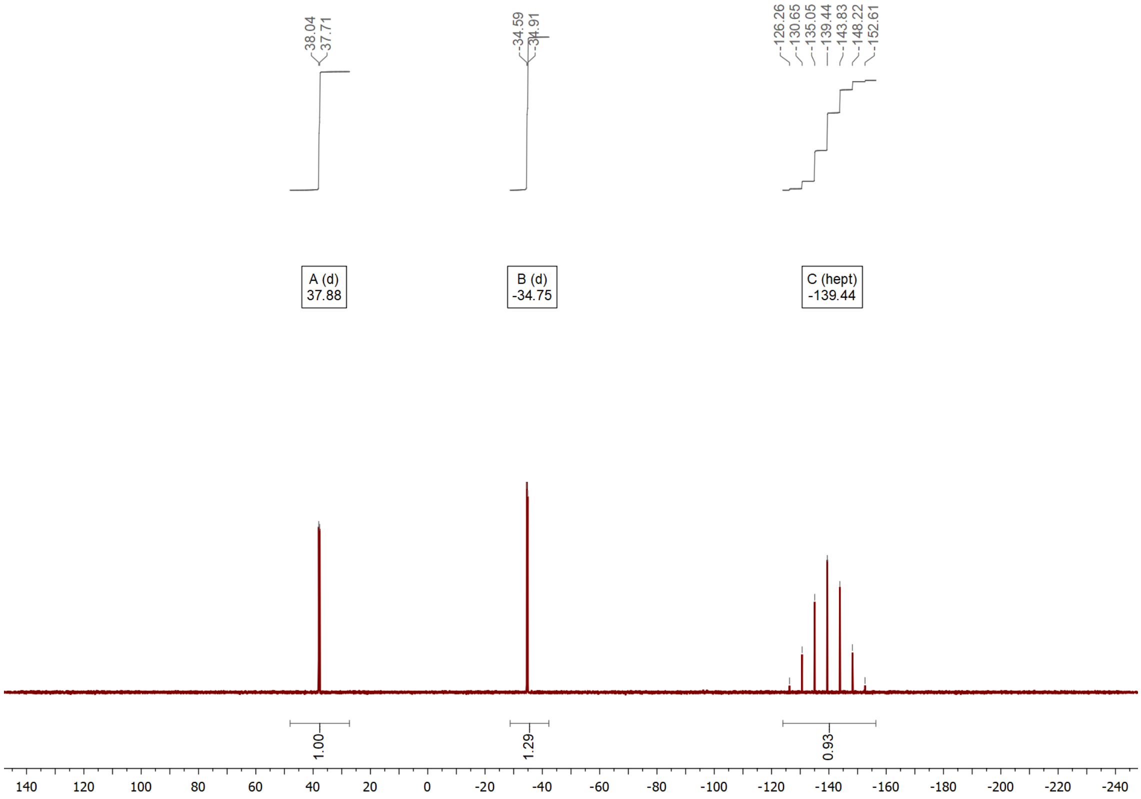

Figure S43. *^{31}P NMR spectra of **10** in DMSO-d$_6$.*

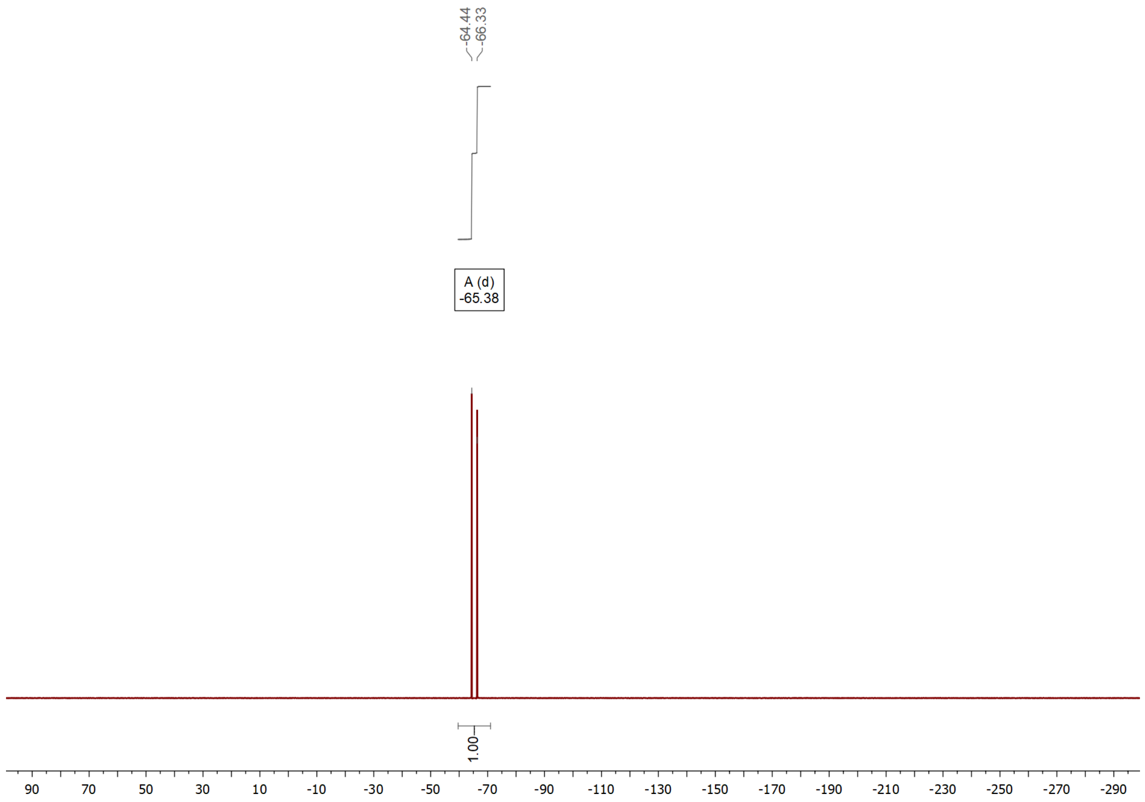

Figure S44. *^{19}F NMR spectra of **10** in DMSO-d$_6$.*

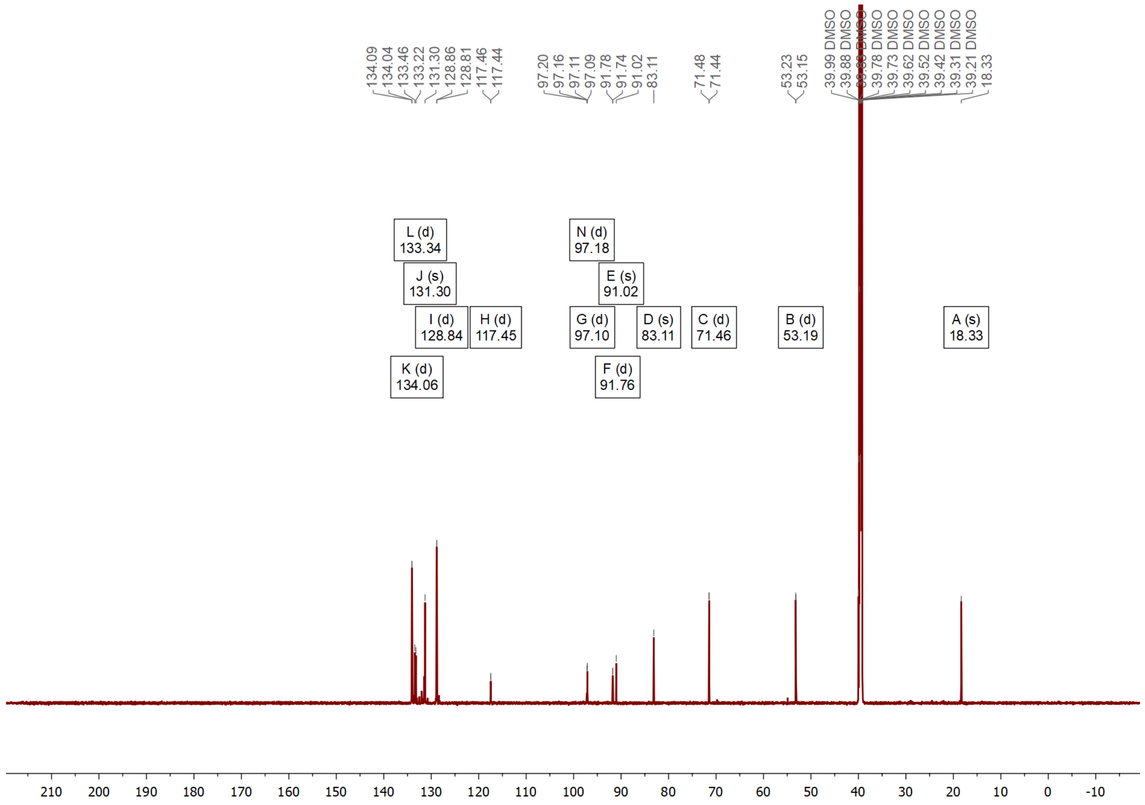

Figure S45. *^{13}C NMR spectra of **10** in DMSO-d$_6$.*

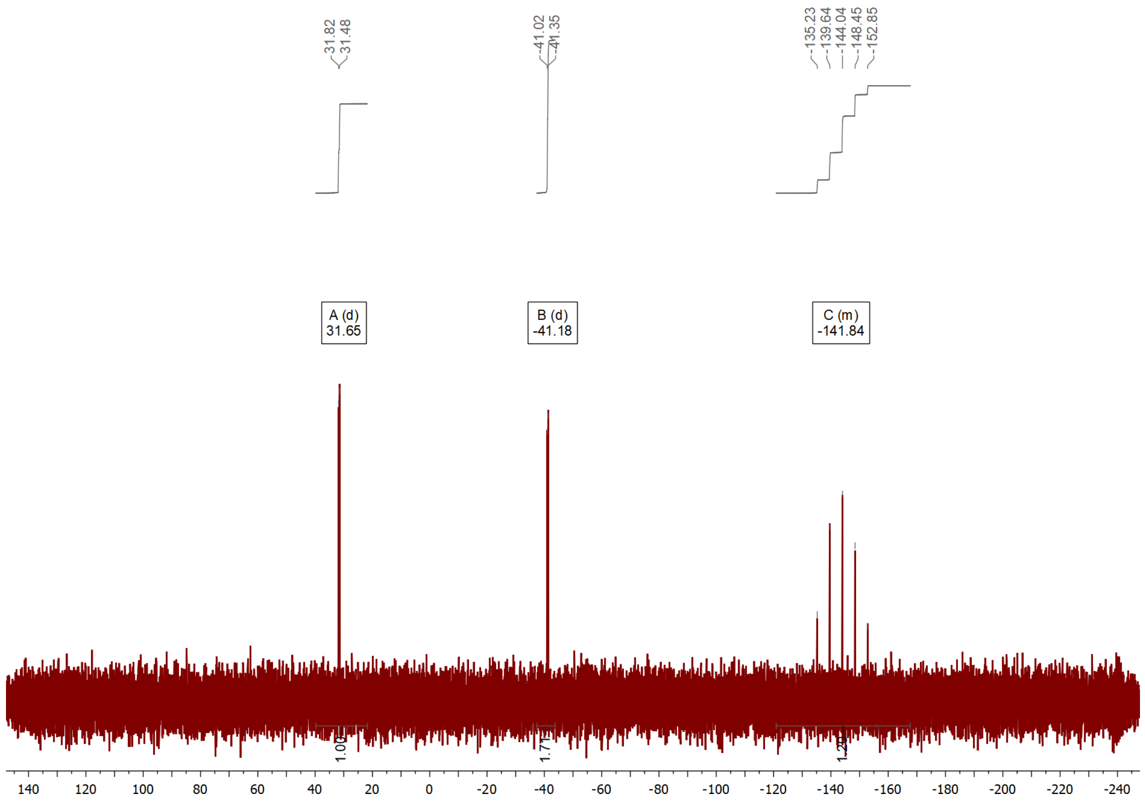

Figure S46. *^{31}P NMR spectra of **10** in chloroform-d.*

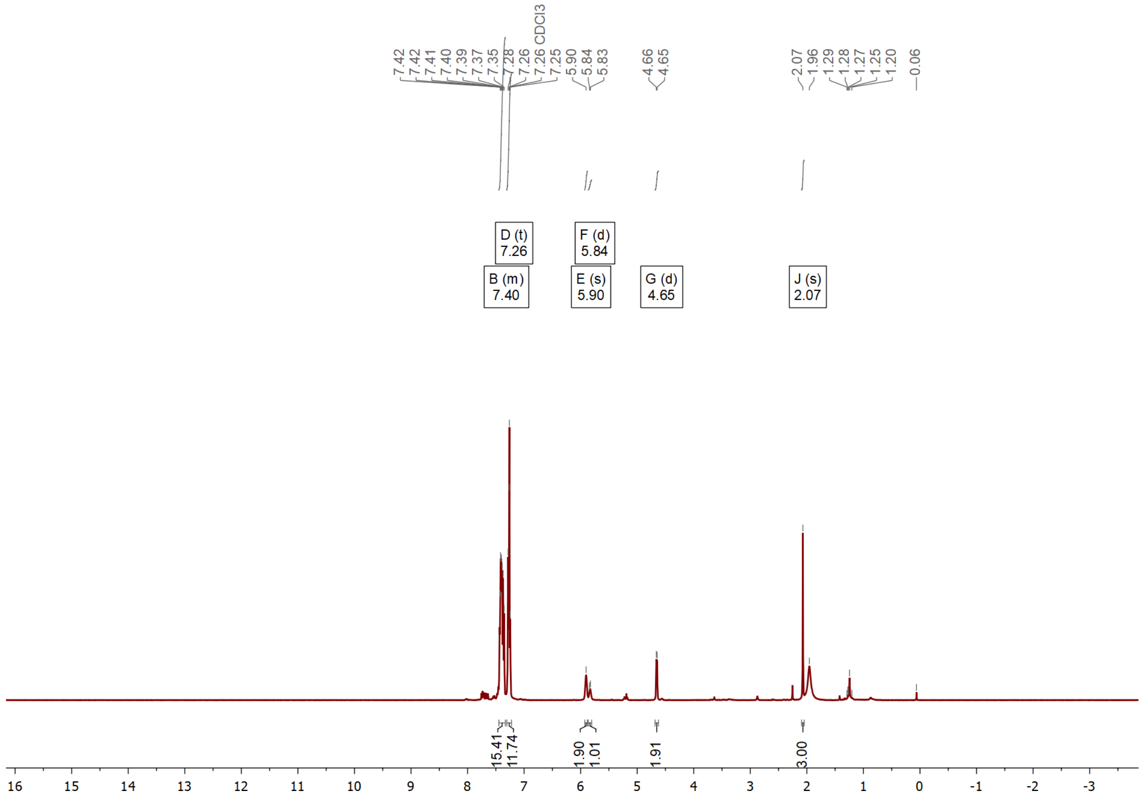

Figure S47. *^{1}H NMR spectra of **11**Cl in chloroform-d.*

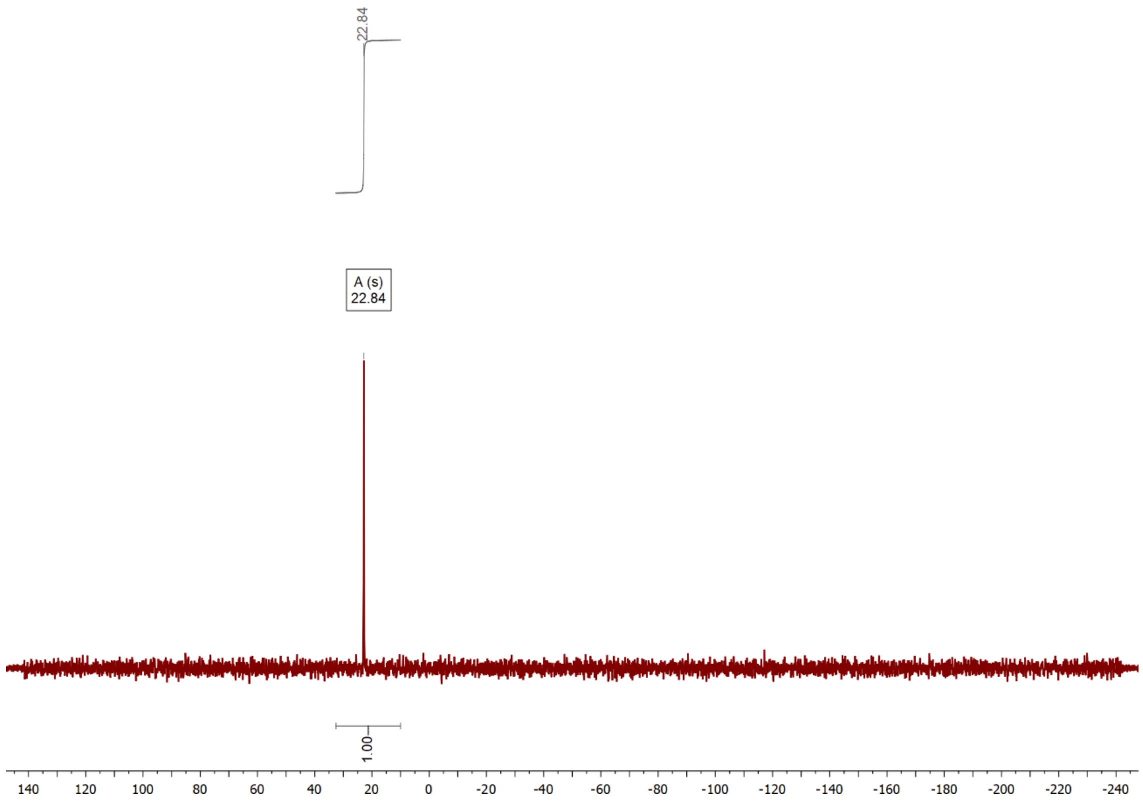

Figure S48. *^{31}P NMR spectra of **11**Cl in chloroform-d.*

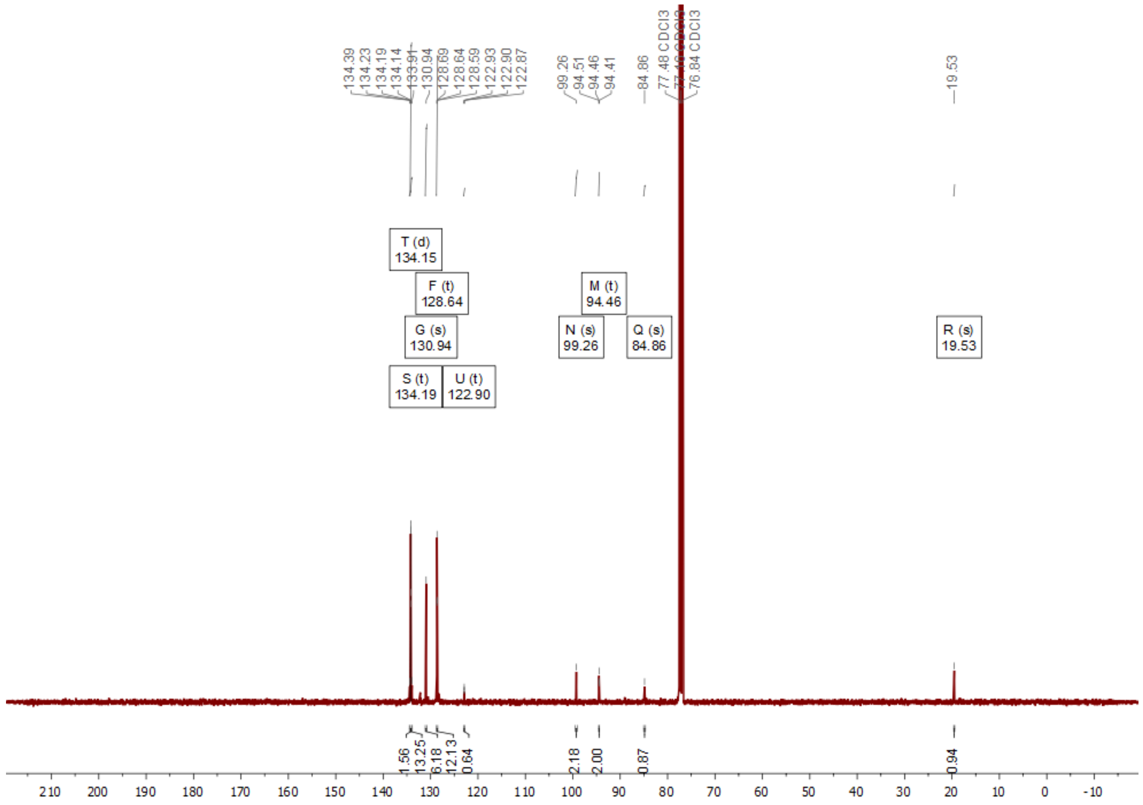

Figure S49. *^{13}C NMR spectra of **11**Cl in chloroform-d.*

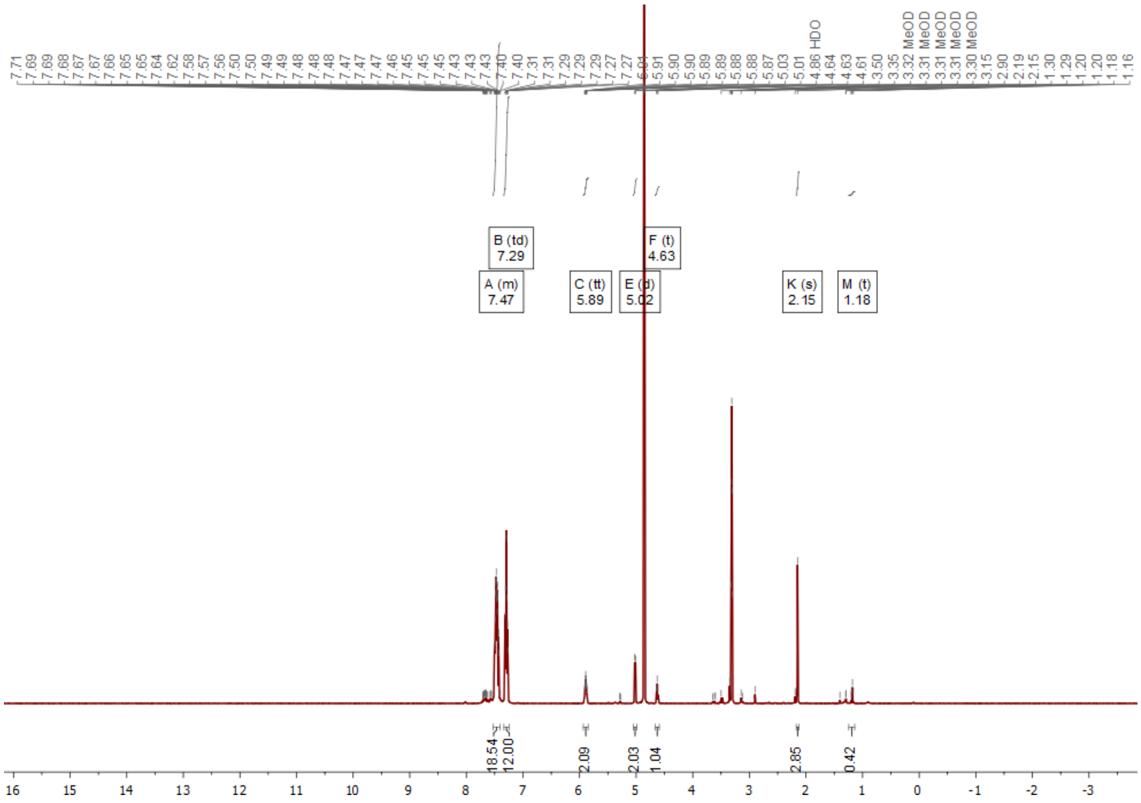

Figure S50. *^{1}H NMR spectra of **11**Cl in methanol-d$_4$.*

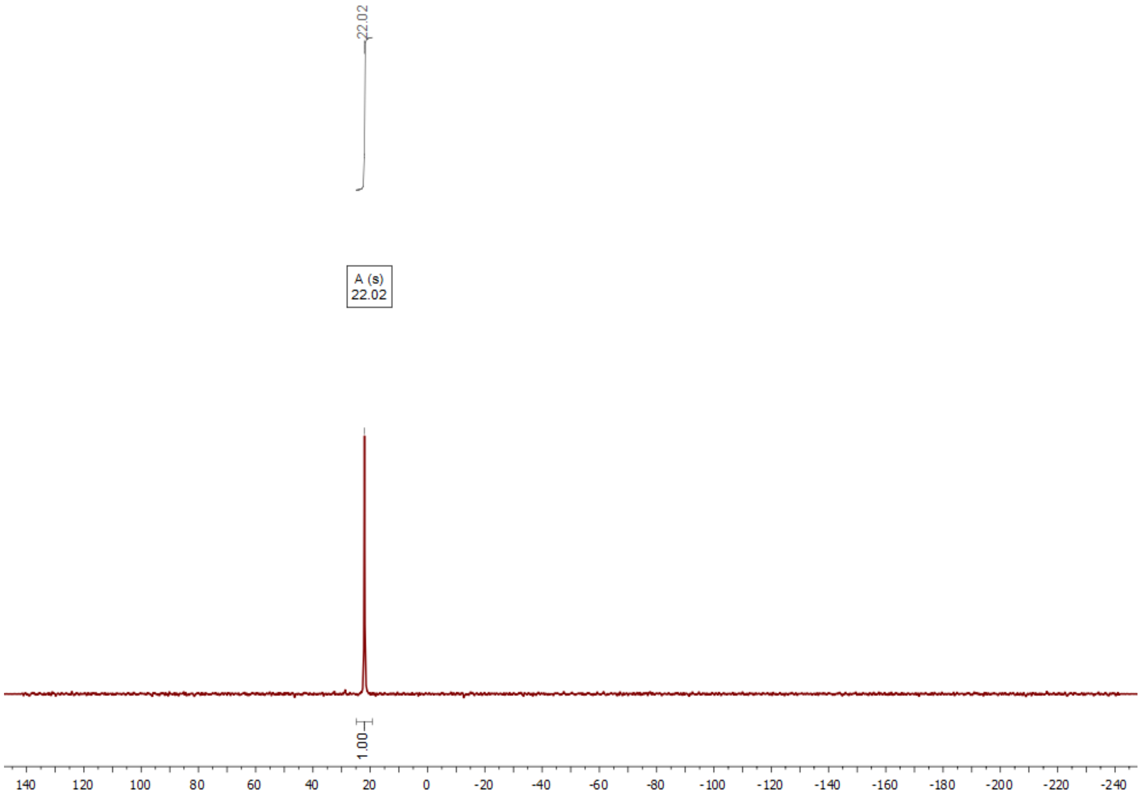

Figure S51. *^{31}P NMR spectra of **11**Cl in methanol-d$_4$.*

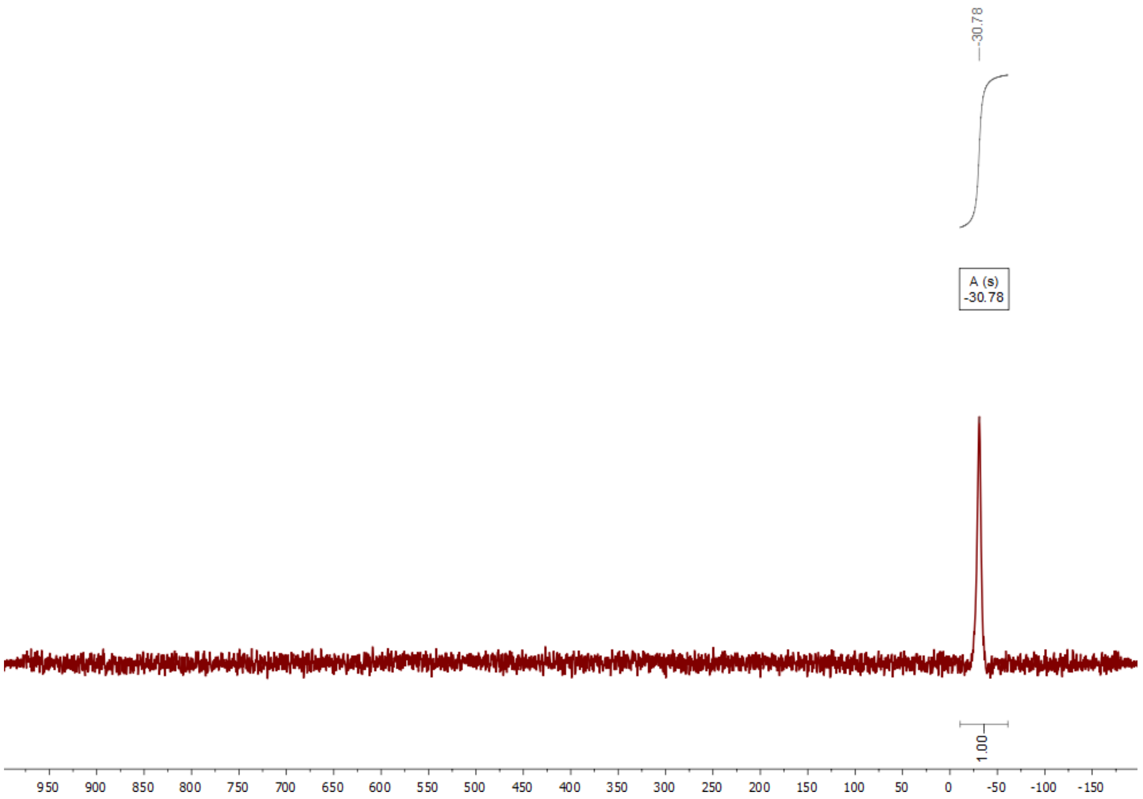

Figure S52. *[37]Cl NMR spectra of* ***11****Cl in methanol-d*$_4$.

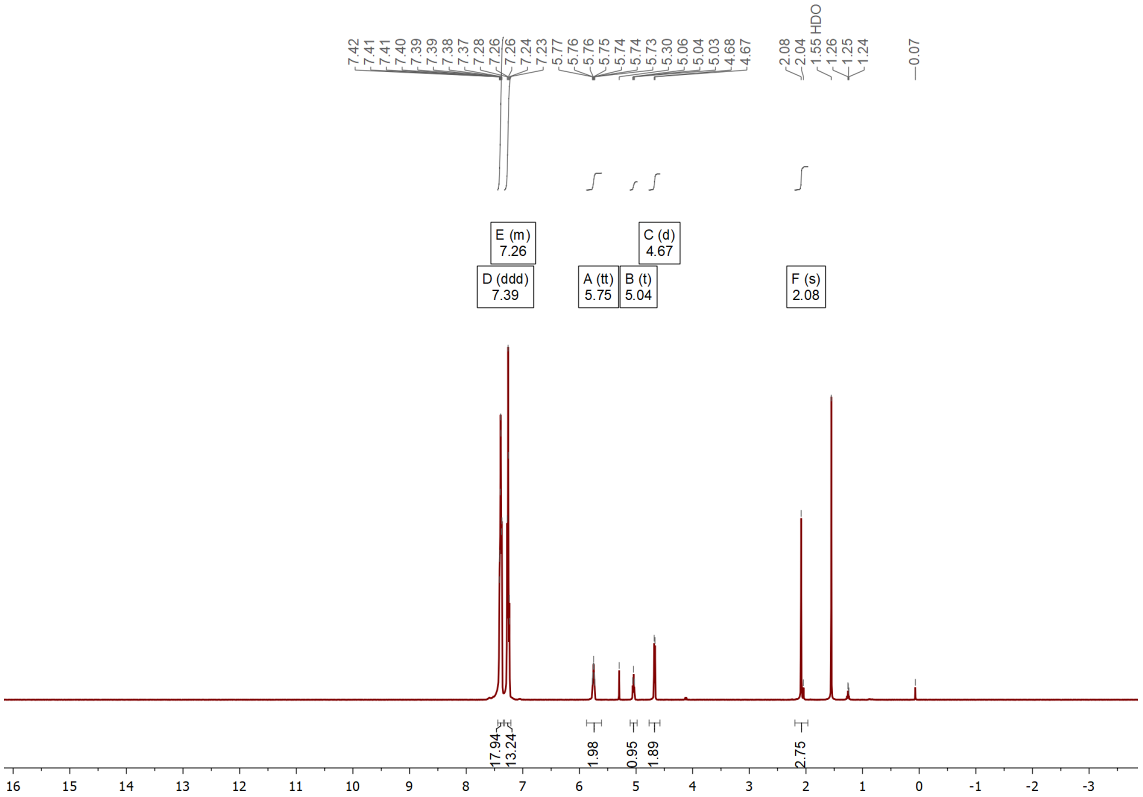

Figure S53. *^{1}H NMR spectra of* ***11****PF*$_6$ *in chloroform-d.*

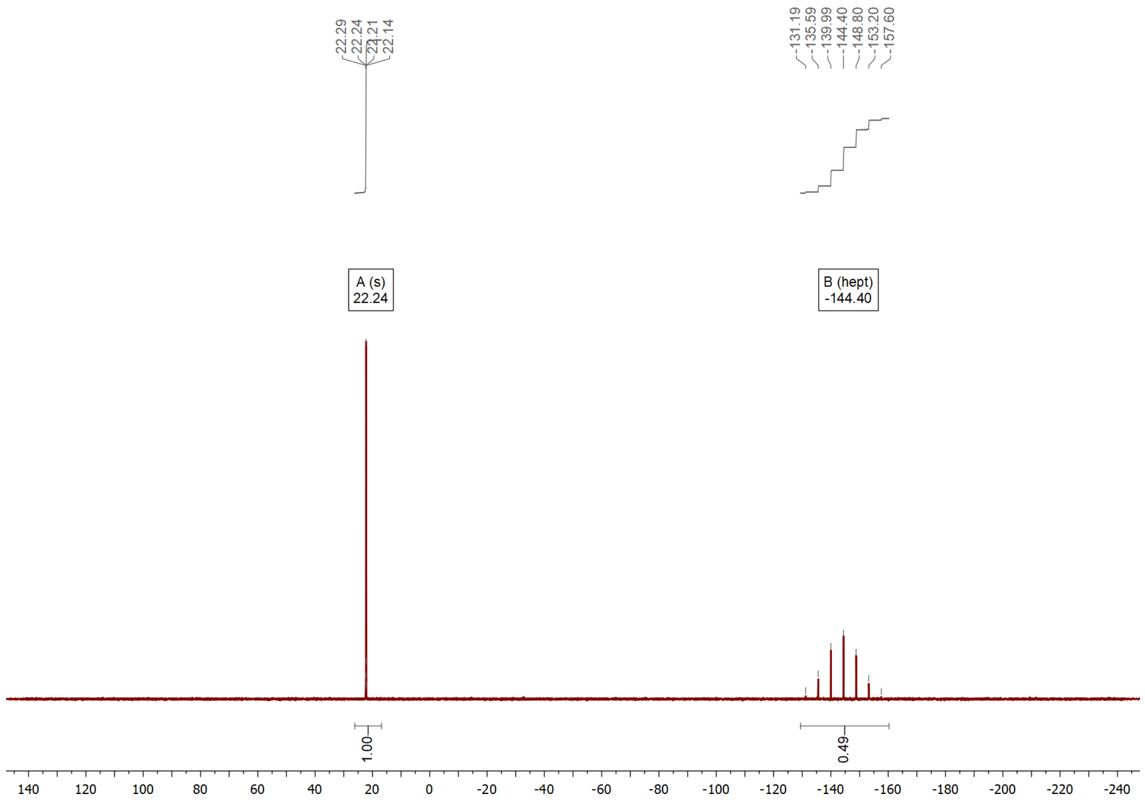

Figure S54. *^{31}P NMR spectra of* ***11****PF_6 in chloroform-d.*

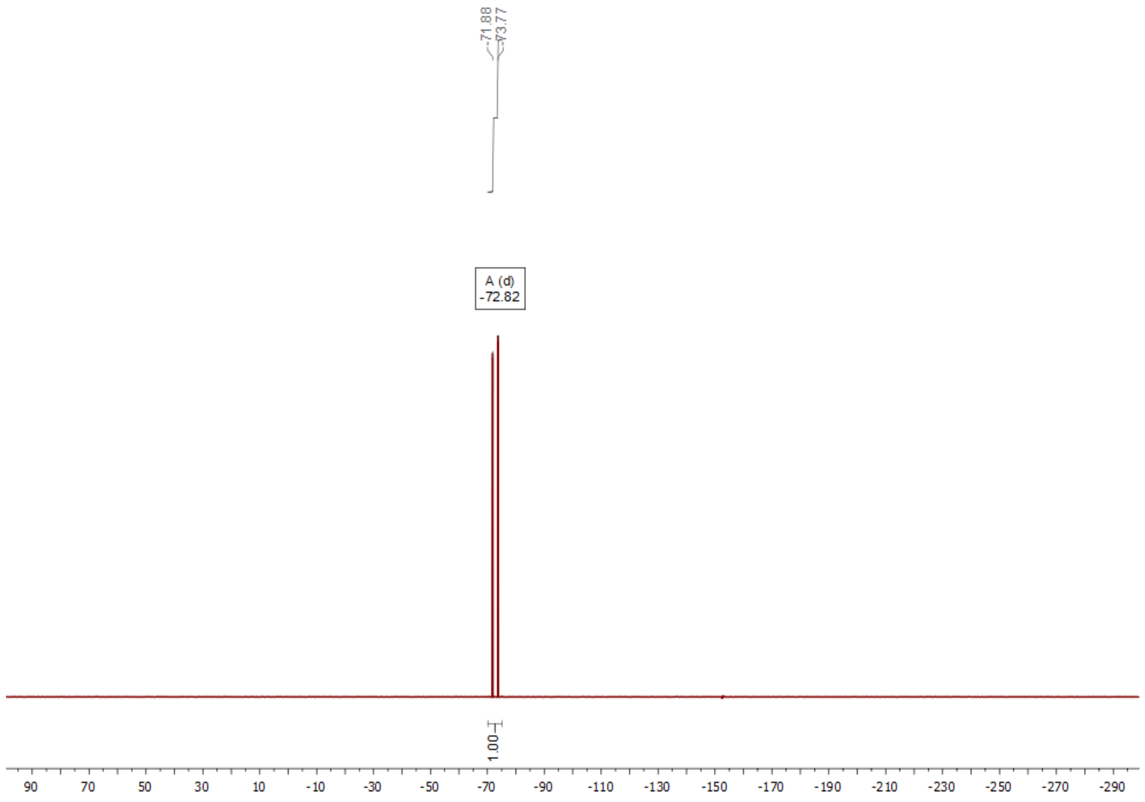

Figure S55. *^{19}F NMR spectra of* ***11****PF_6 in chloroform-d.*

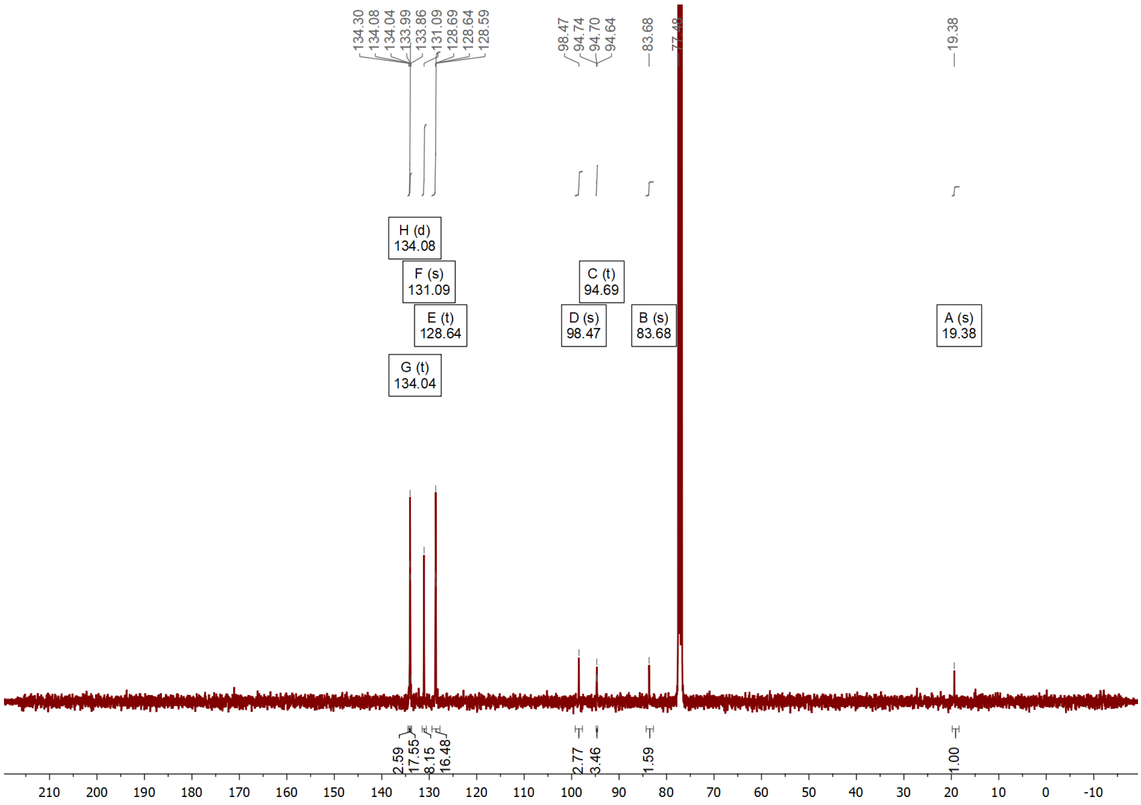

Figure S56. *^{13}C NMR spectra of **11**PF_6 in chloroform-d.*

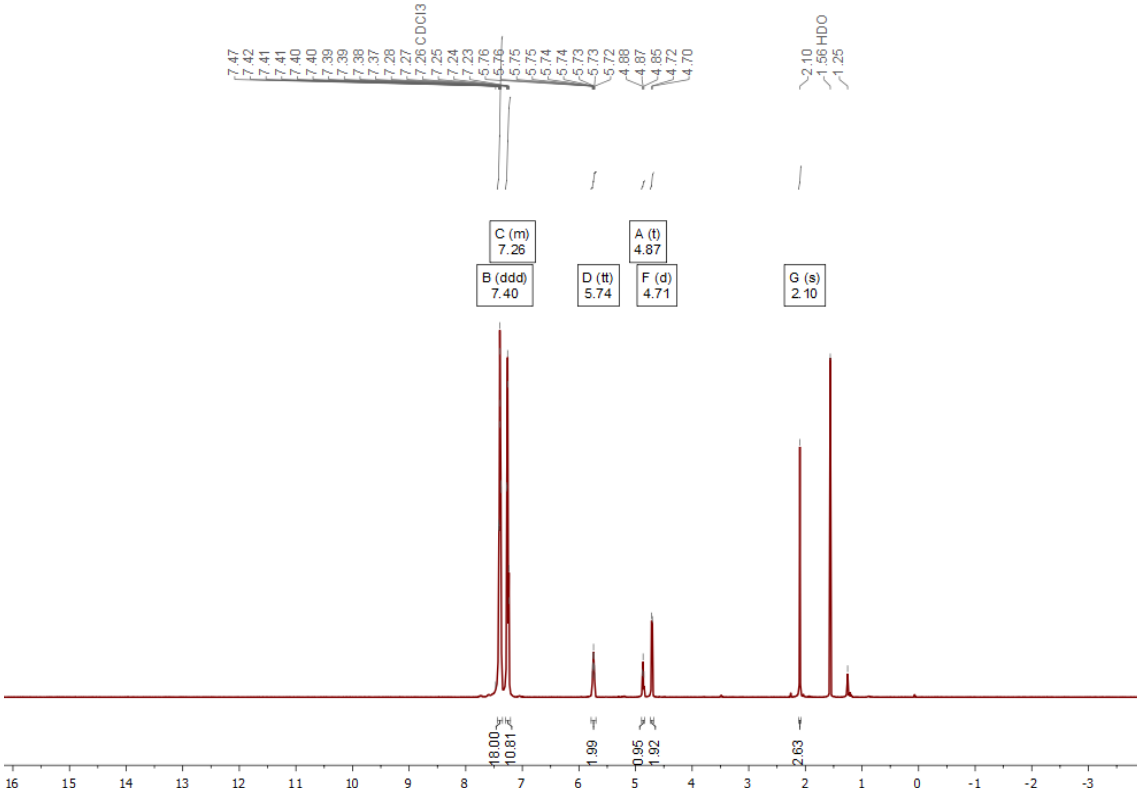

Figure S57. *^{1}H NMR spectra of **11**SbF_6 in chloroform-d.*

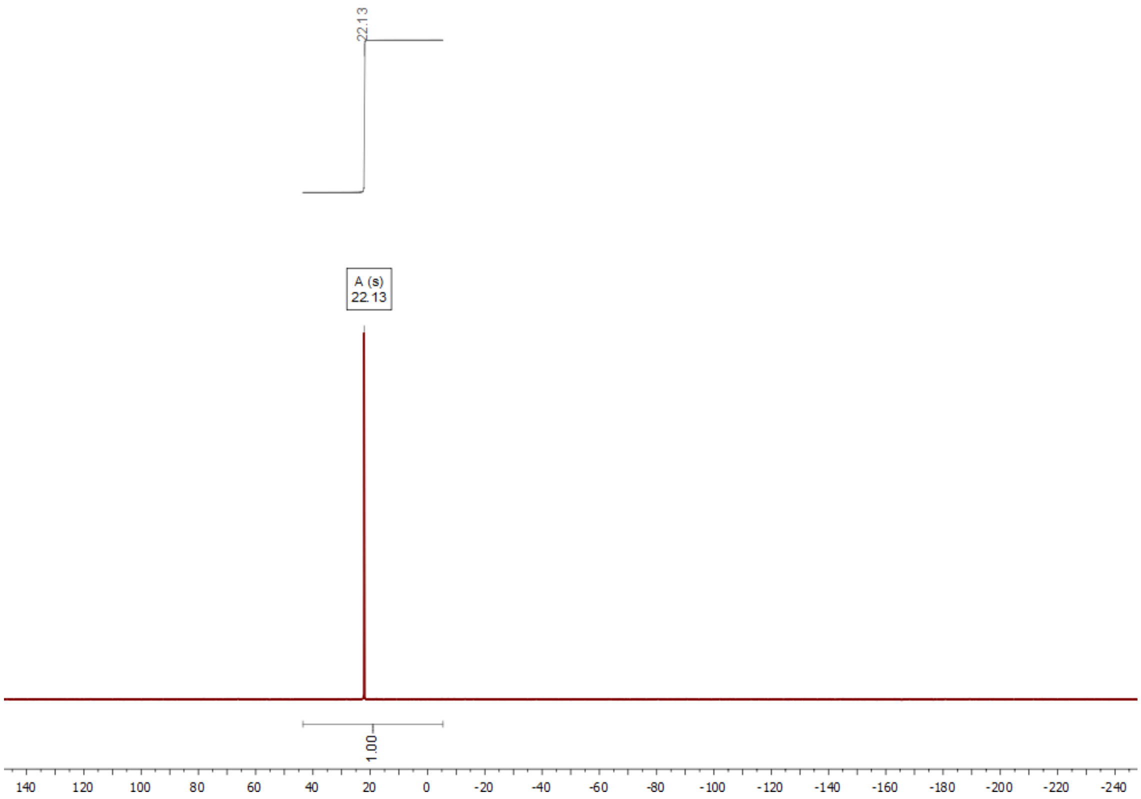

Figure S58. *^{31}P NMR spectra of **11**SbF$_6$ in chloroform-d.*

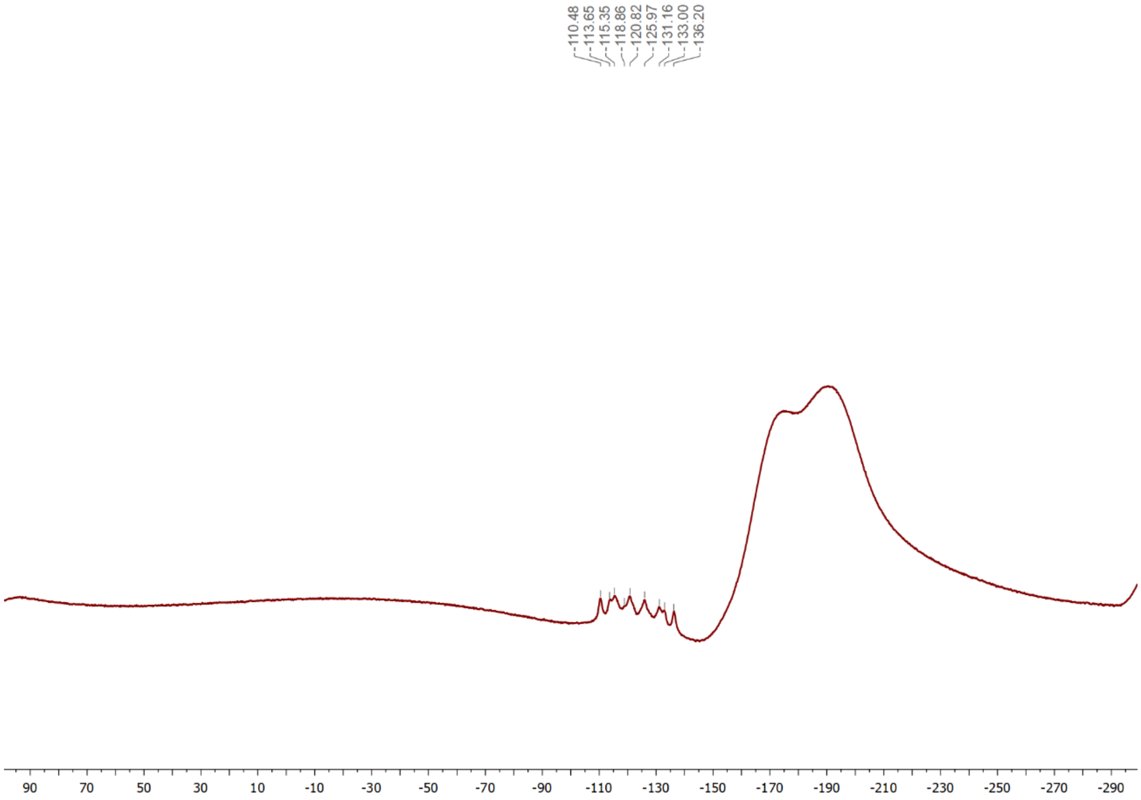

Figure S59. *^{19}F NMR spectra of **11**SbF$_6$ in chloroform-d.*

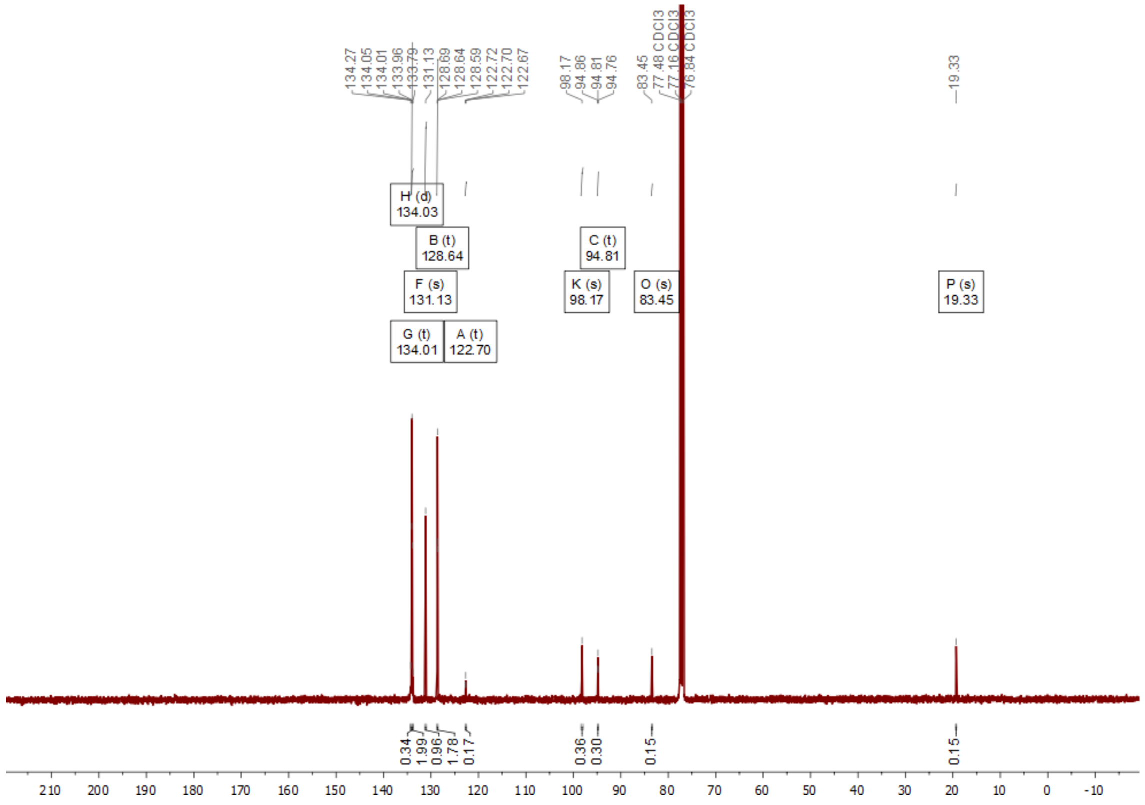

Figure S60. *^{13}C NMR spectra of **11**SbF_6 in chloroform-d.*

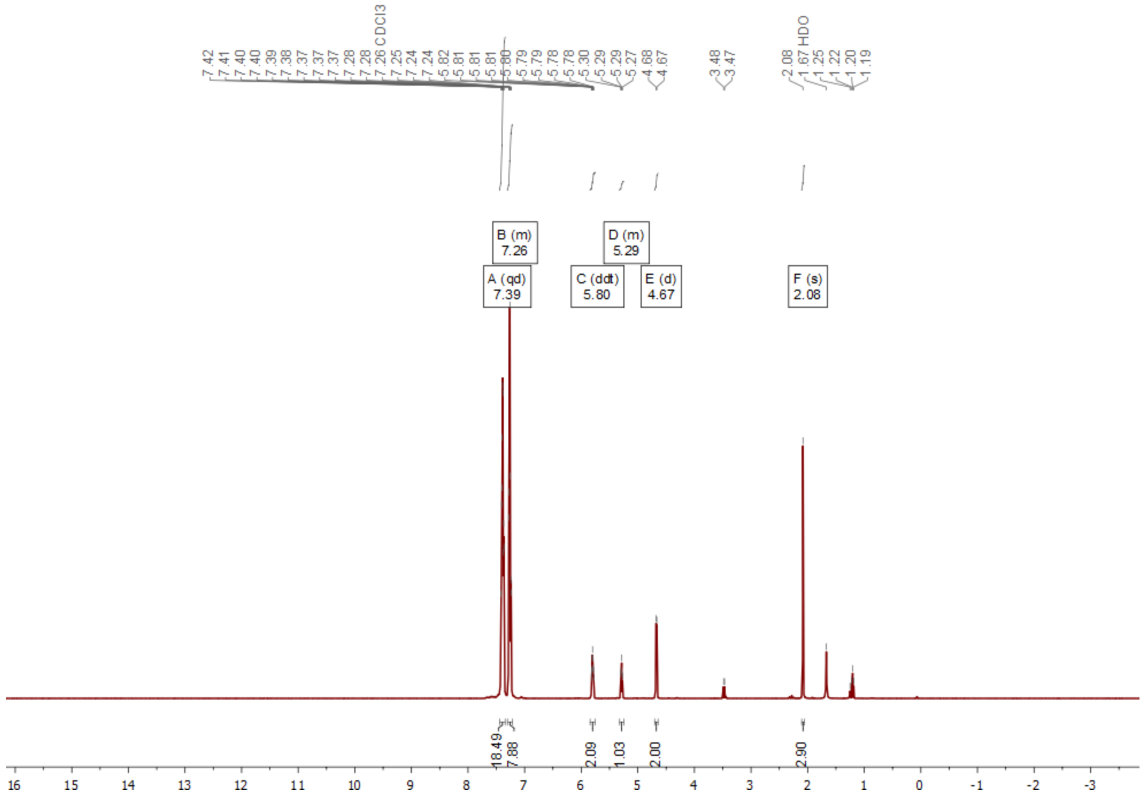

Figure S61. *^{1}H NMR spectra of **11**OTf in chloroform-d.*

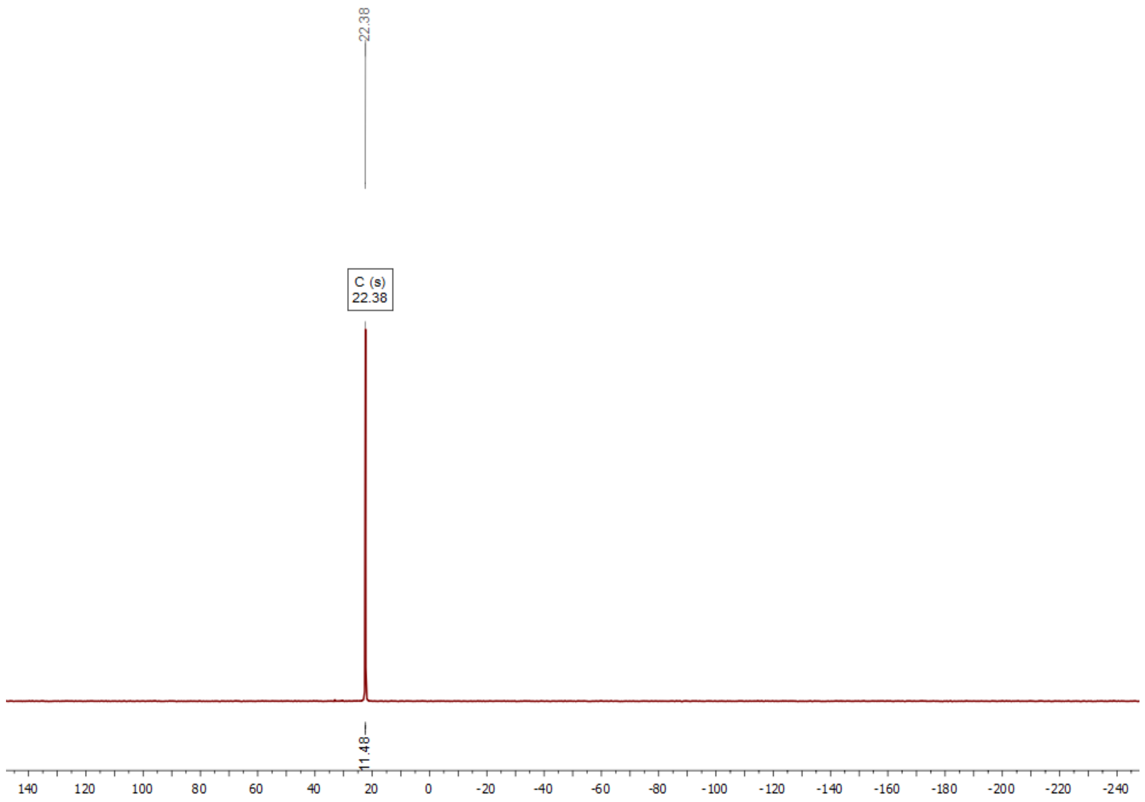

Figure S62. *^{31}P NMR spectra of **11**OTf in chloroform-d.*

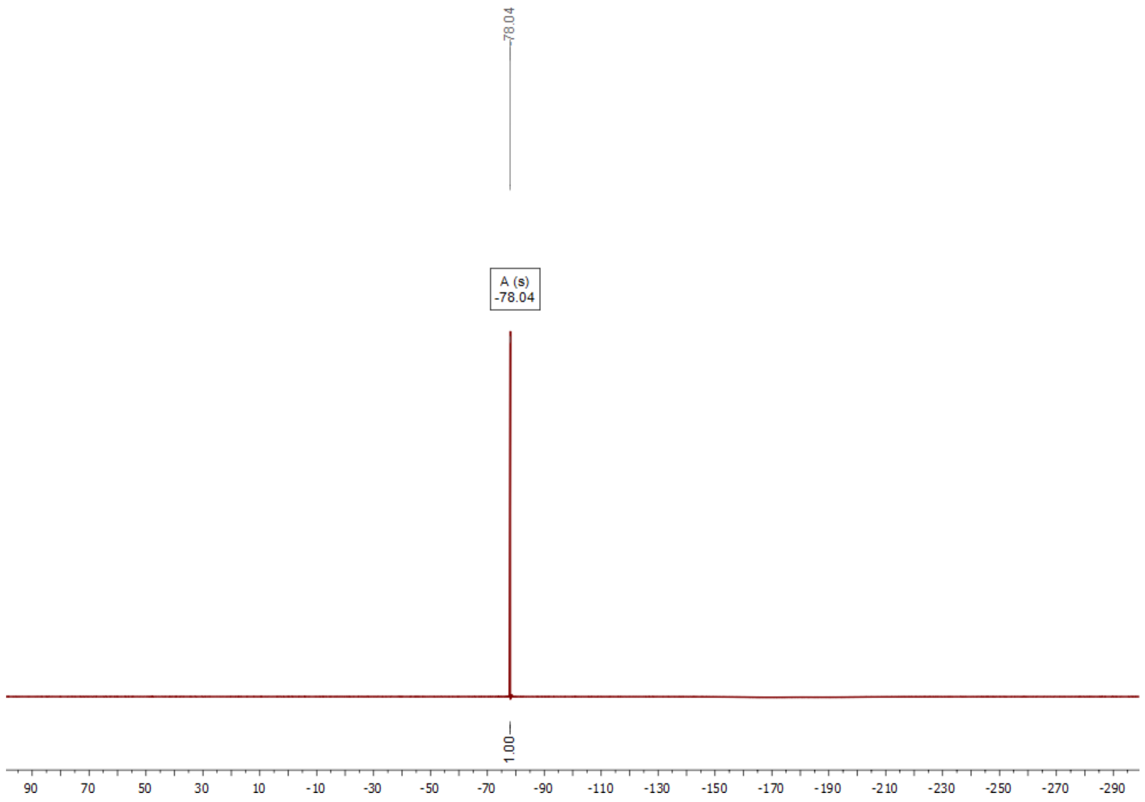

Figure S63. *^{19}F NMR spectra of **11**OTf in chloroform-d.*

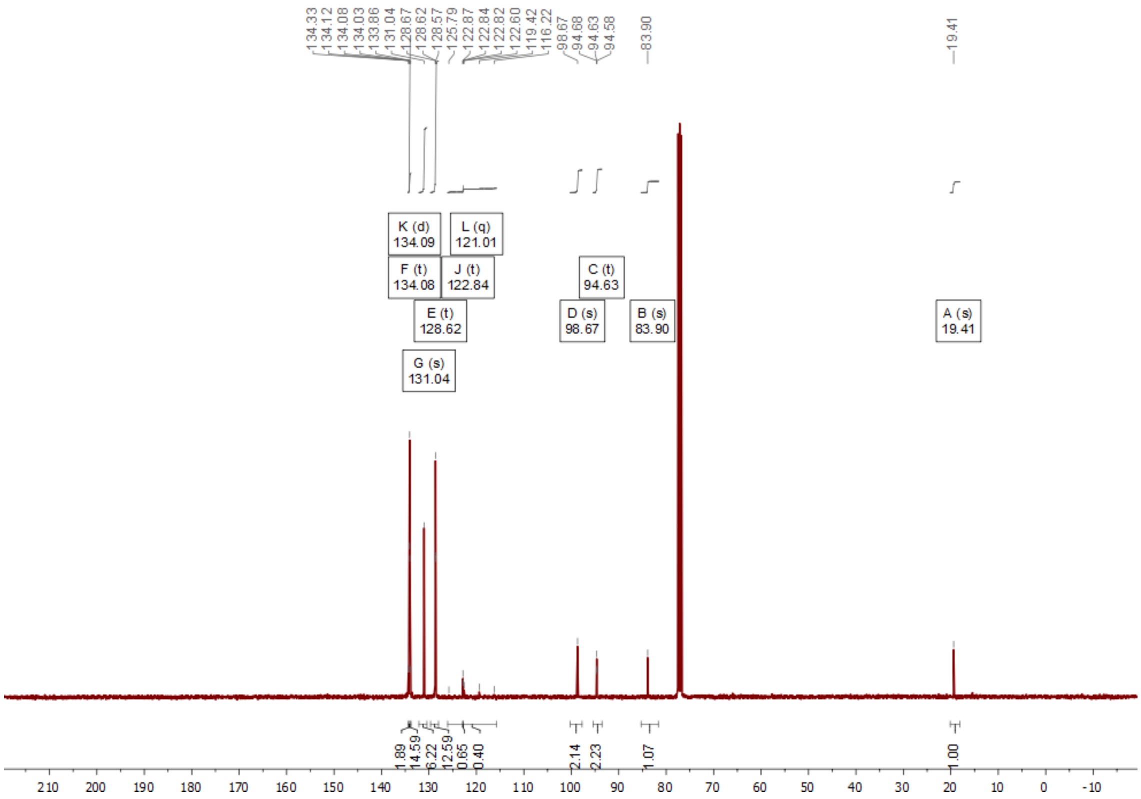

Figure S64. *^{13}C NMR spectra of* ***11****OTf in chloroform-d.*

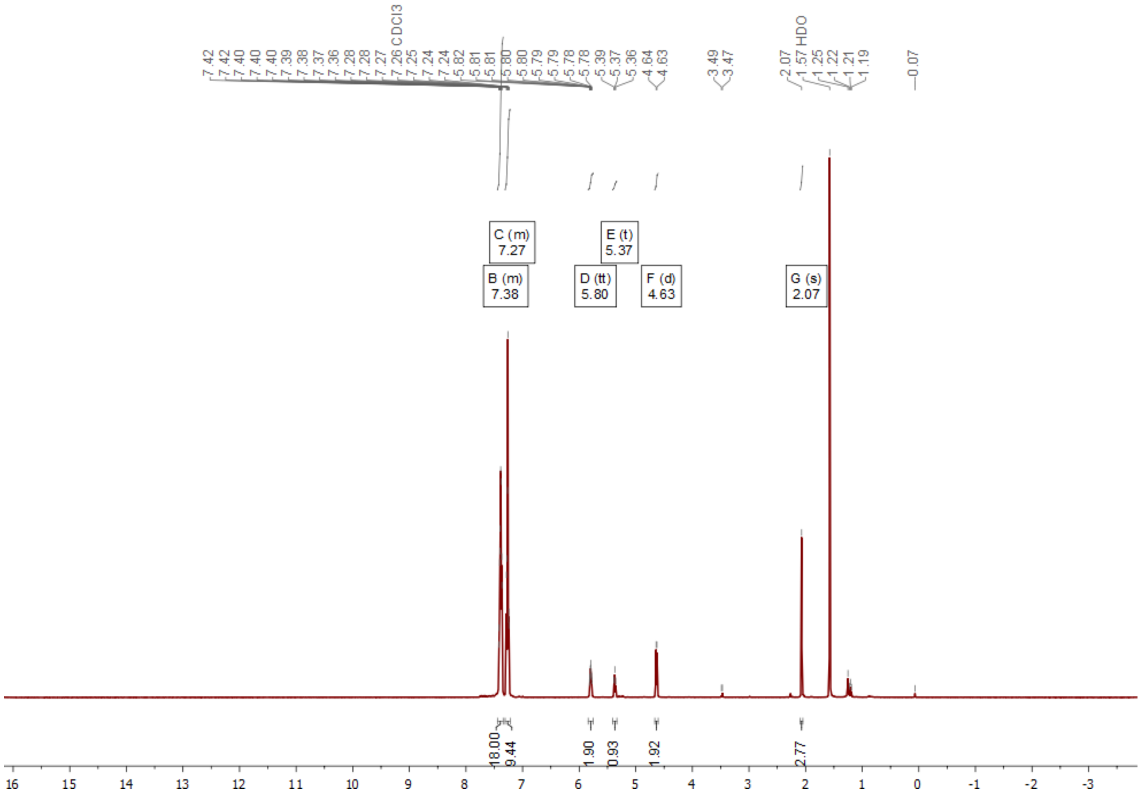

Figure S65. *^{1}H NMR spectra of* ***11****BF_4 in chloroform-d.*

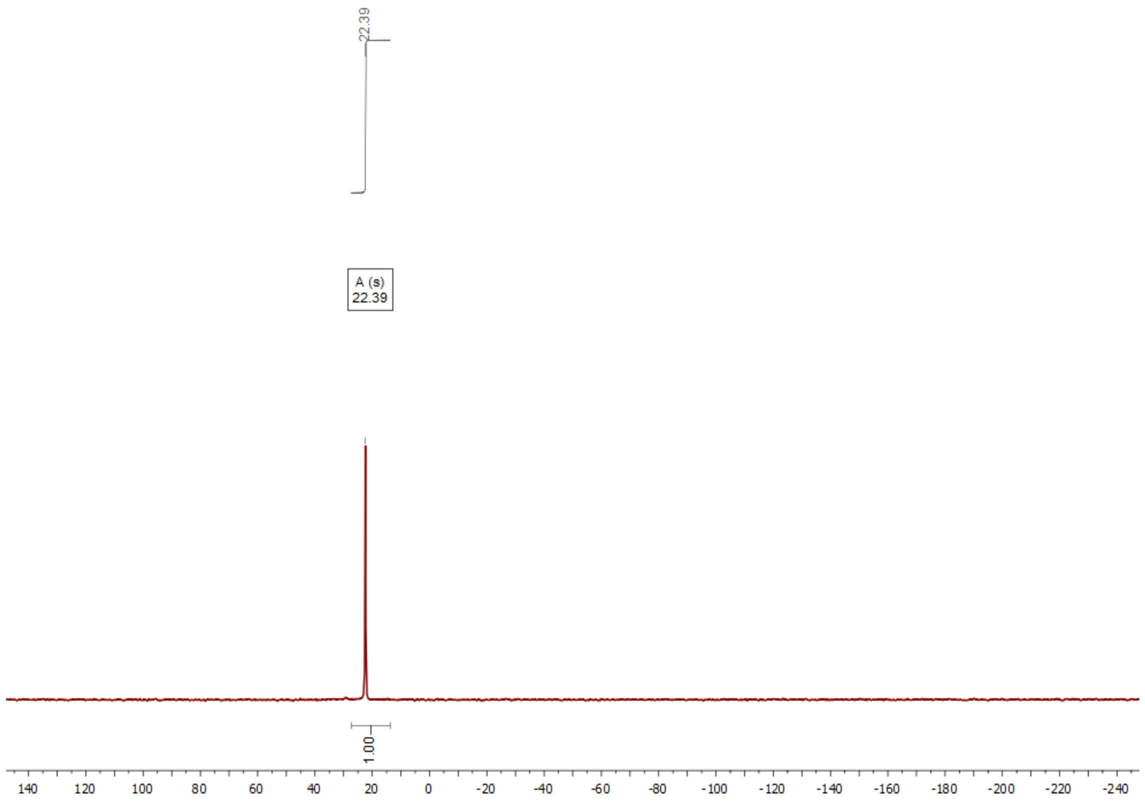

Figure S66. *^{31}P NMR spectra of **11**BF_4 in chloroform-d.*

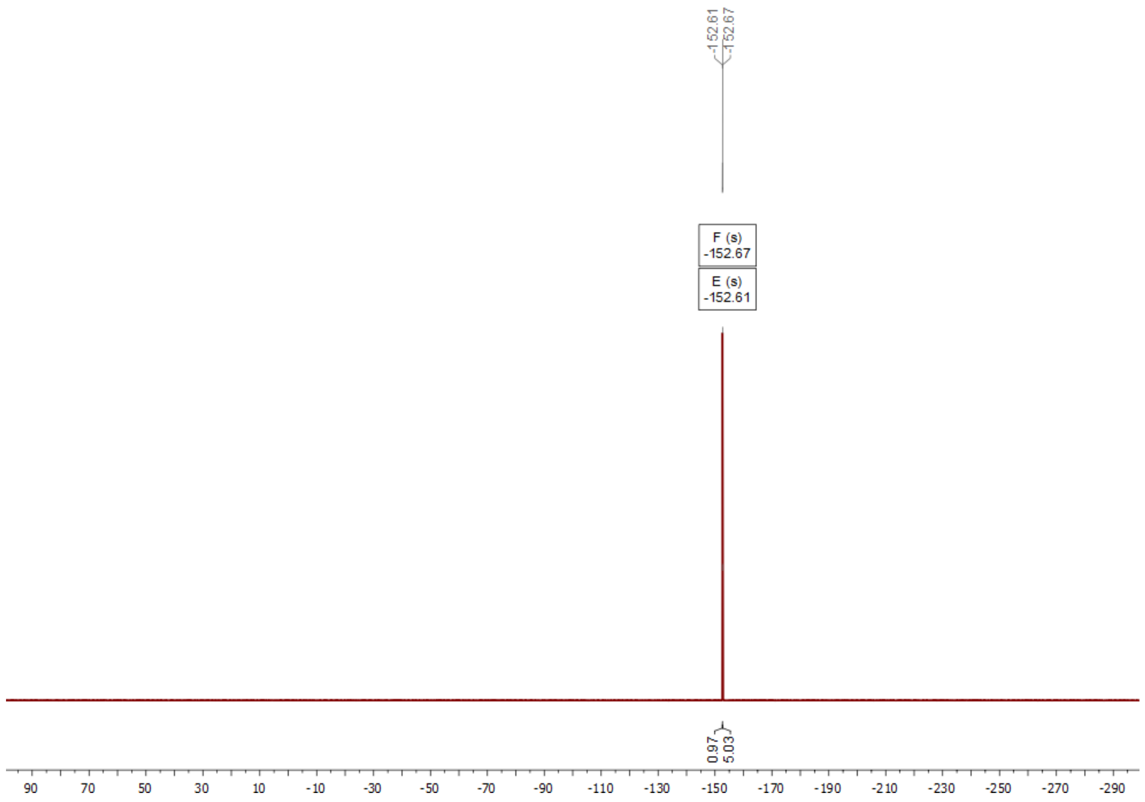

Figure S67. *^{19}F NMR spectra of **11**BF_4 in chloroform-d.*

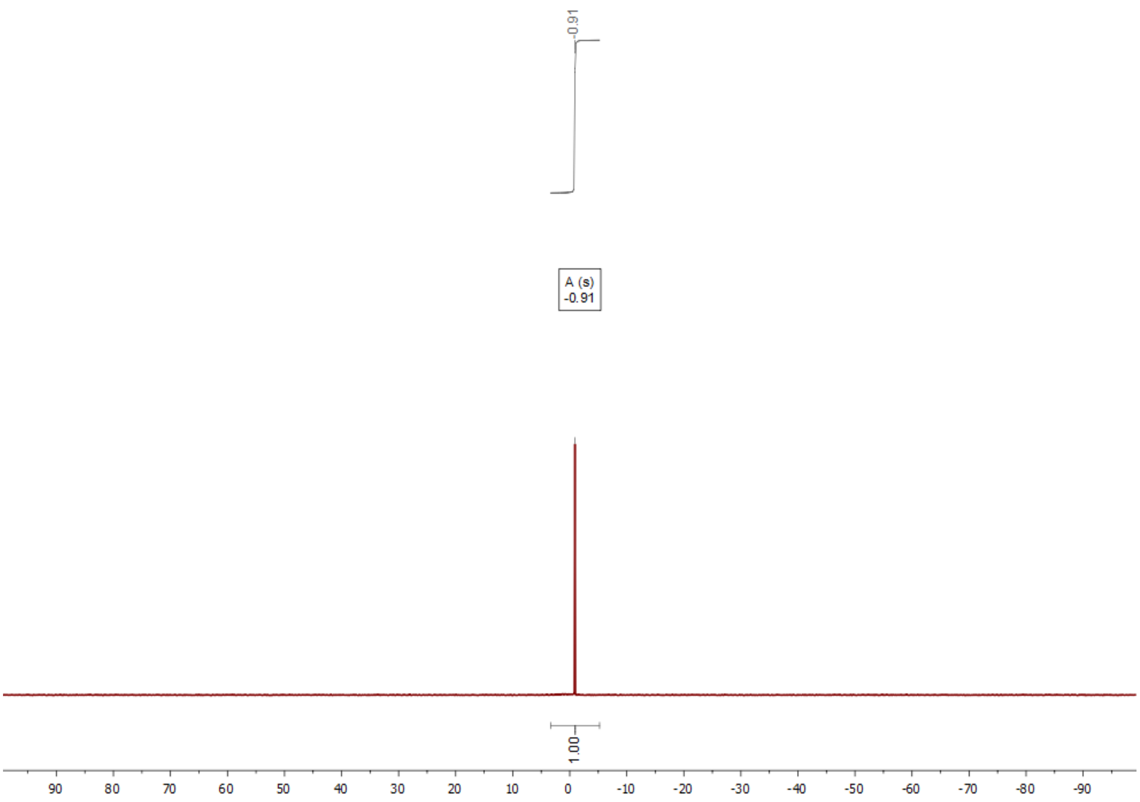

Figure S68. *^{11}B NMR spectra of **11**BF_4 in chloroform-d.*

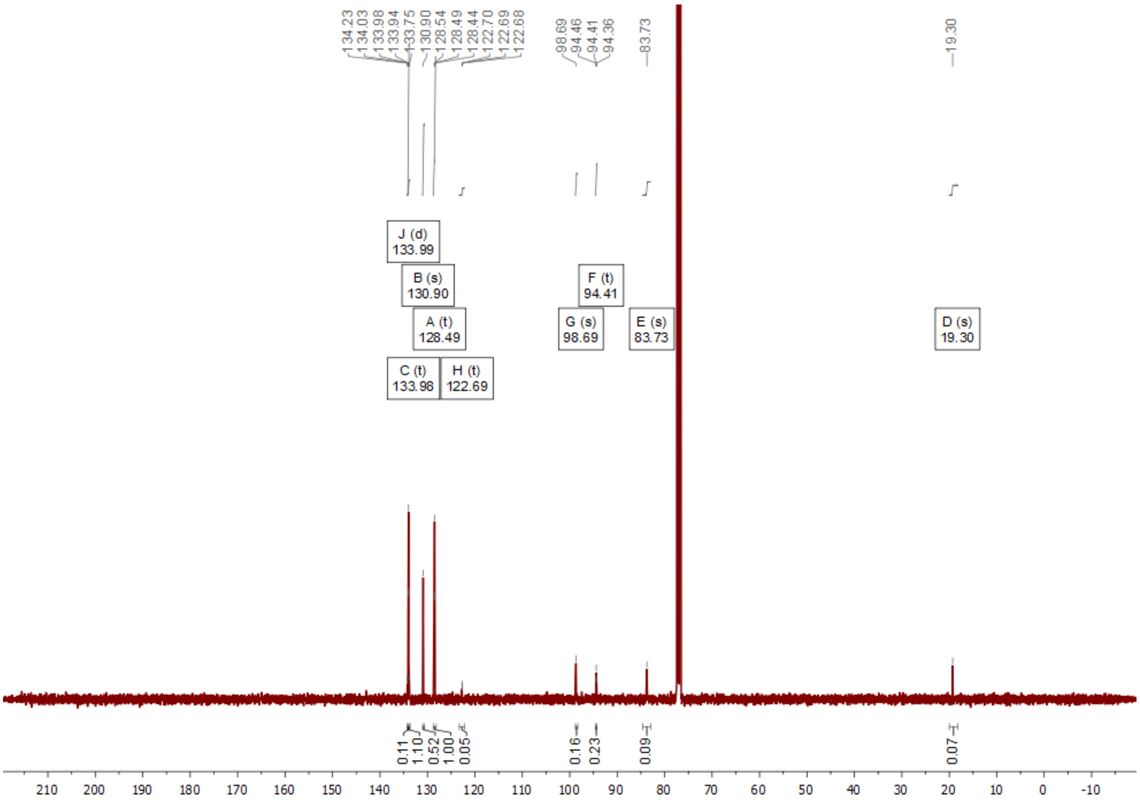

Figure S69. *^{13}C NMR spectra of **11**BF_4 in chloroform-d.*

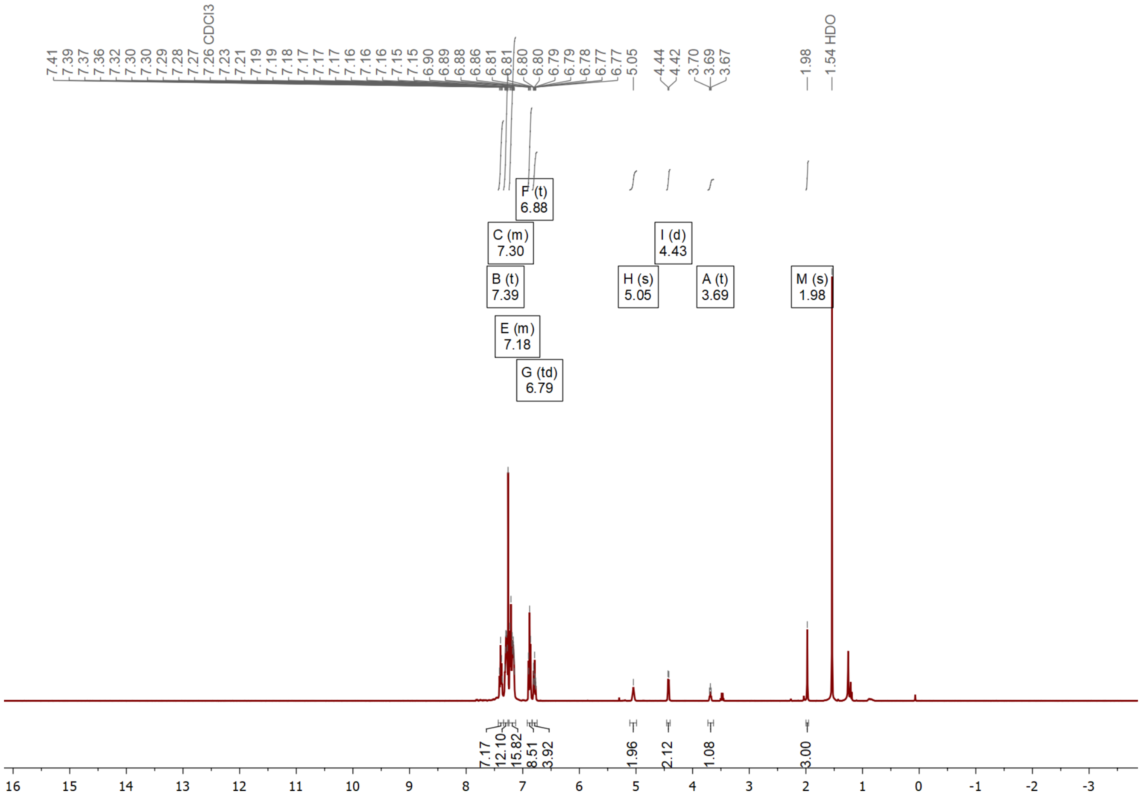

Figure S70. *^{1}H NMR spectra of* ***11***BPh_4 *in chloroform-d.*

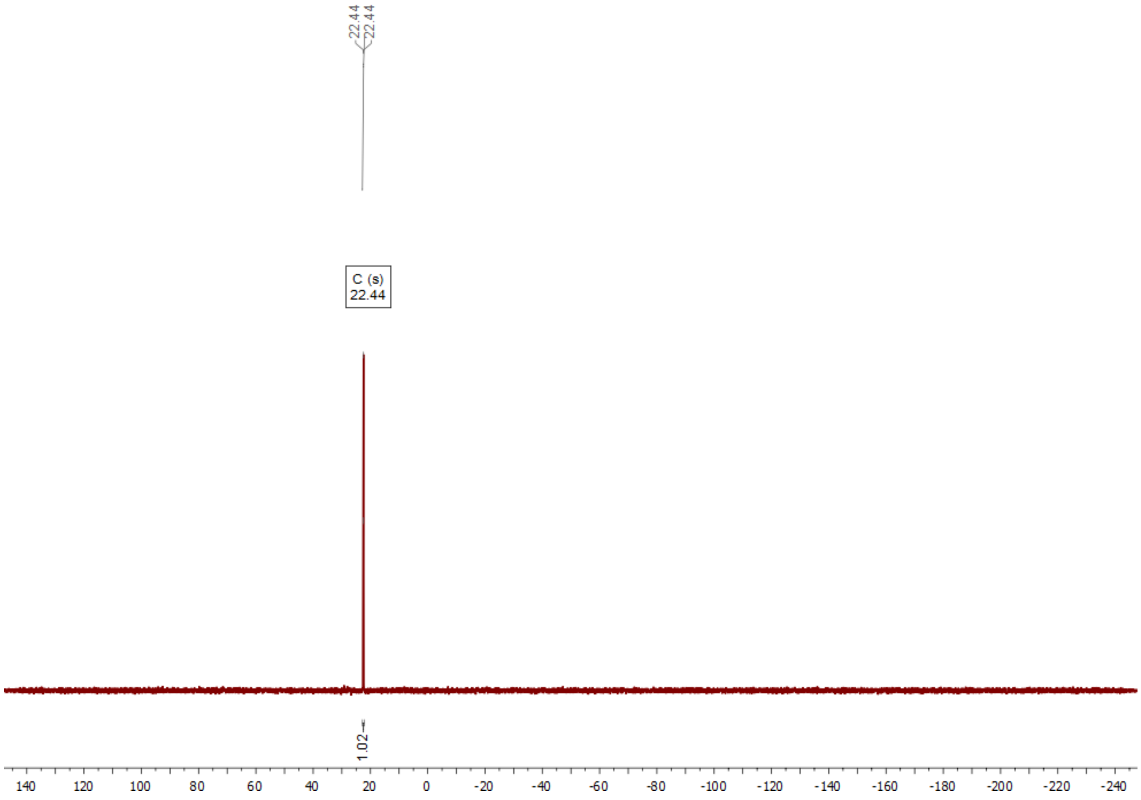

Figure S71. *^{31}P NMR spectra of* ***11***BPh_4 *in chloroform-d.*

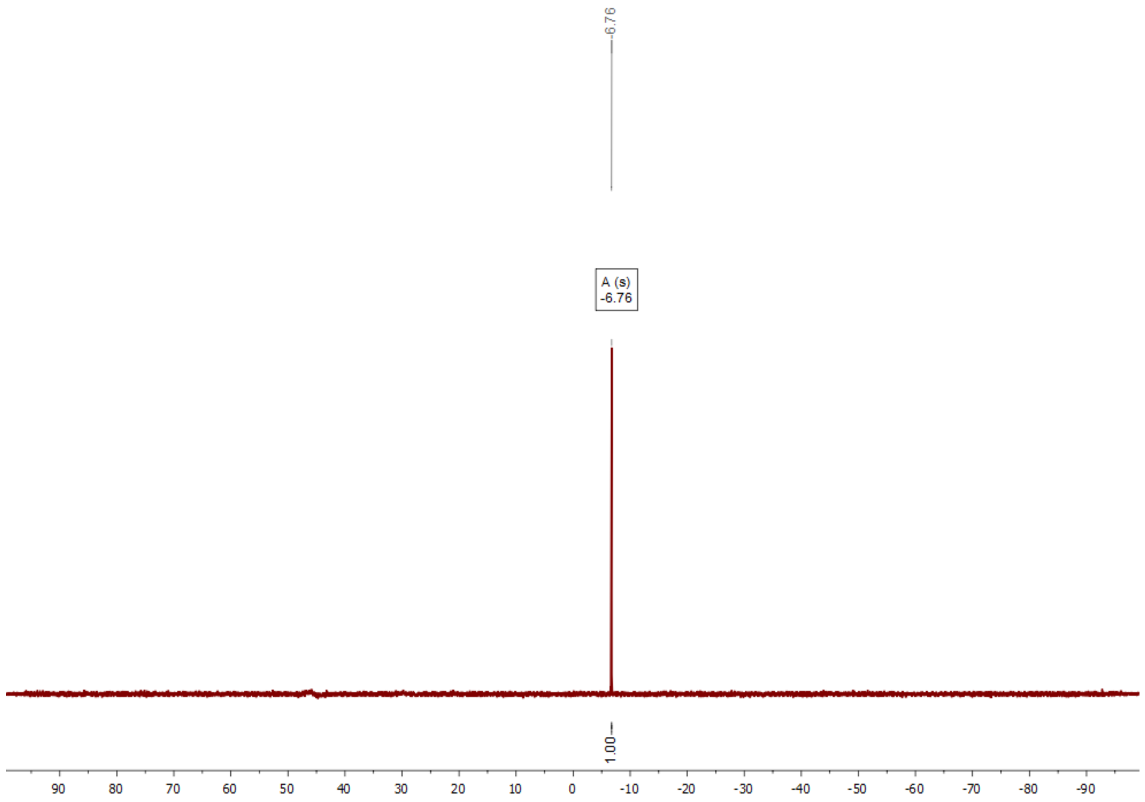

Figure S72. *^{11}B NMR spectra of **11**BPh$_4$ in chloroform-d.*

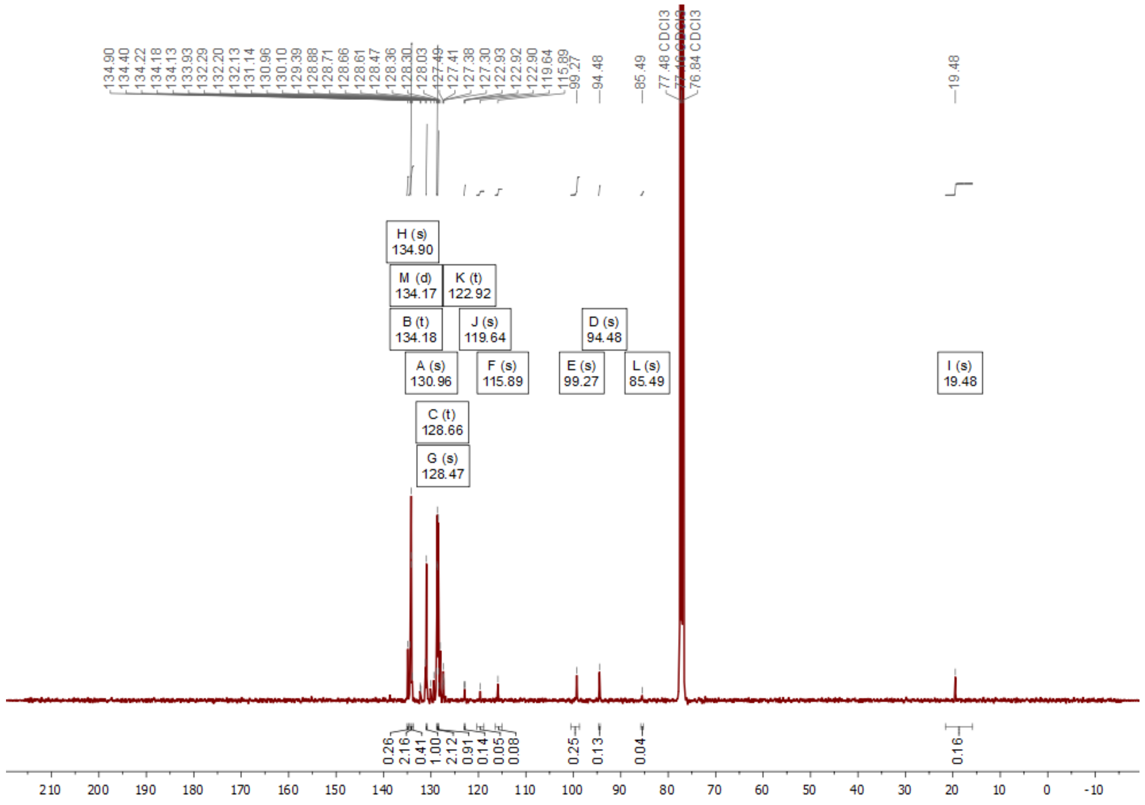

Figure S73. *^{13}C NMR spectra of **11**BPh$_4$ in chloroform-d.*

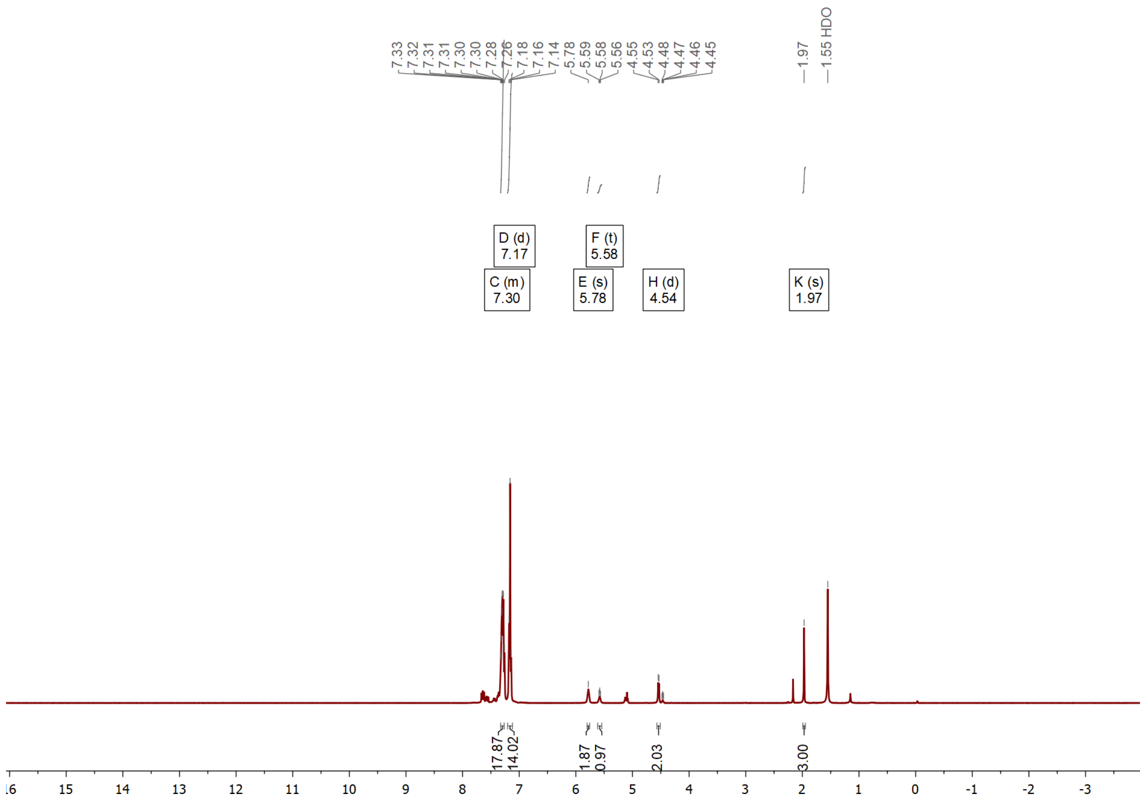

Figure S74. *^{1}H NMR spectra of **11**NO$_3$ in chloroform-d.*

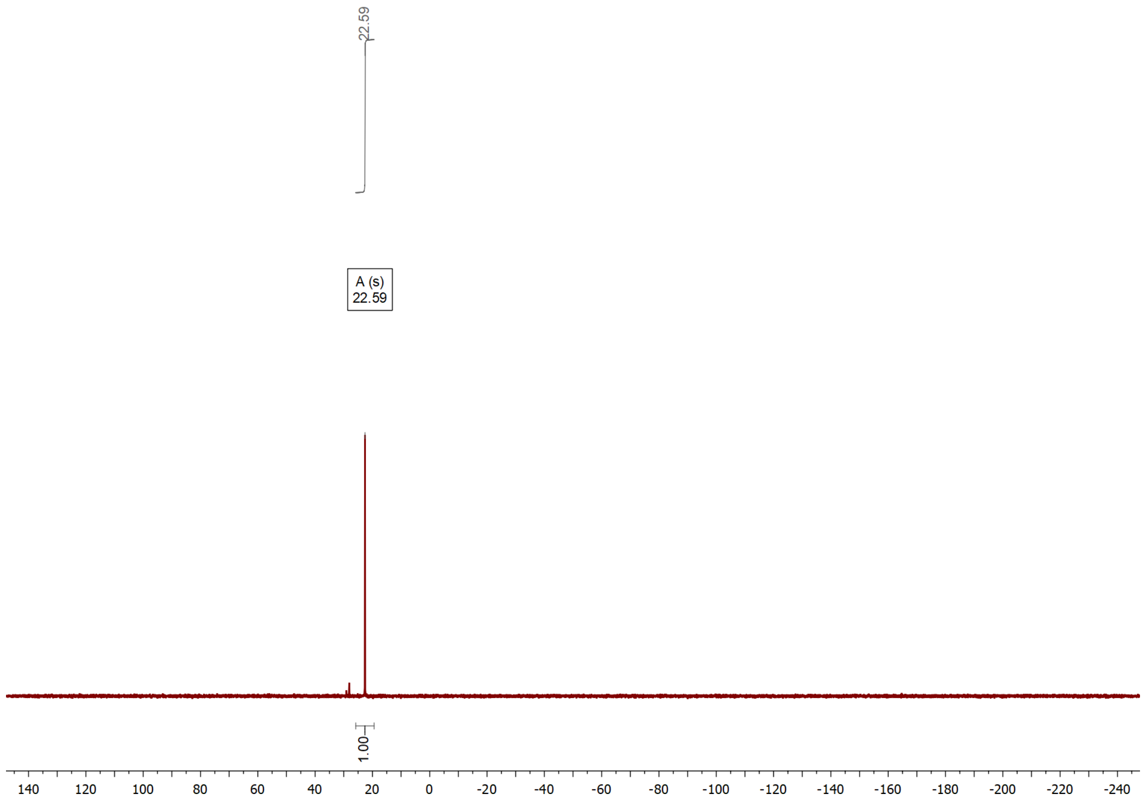

Figure S75. *^{31}P NMR spectra of **11**NO$_3$ in chloroform-d.*

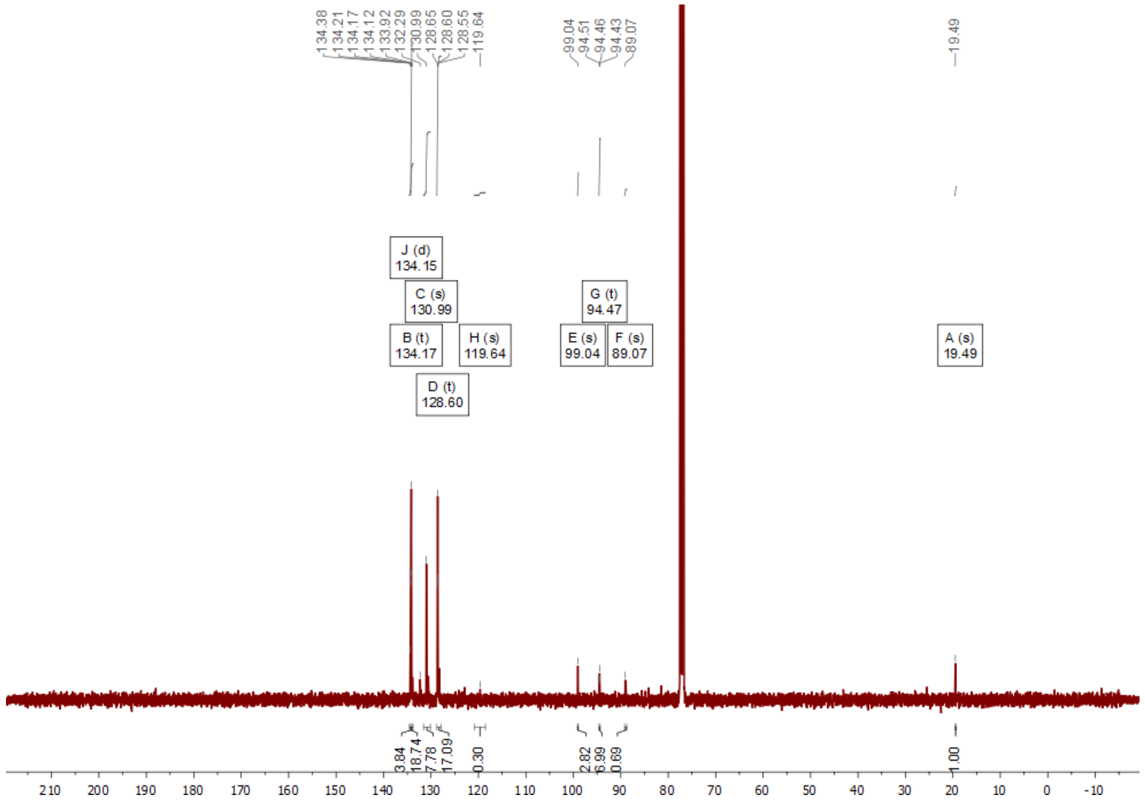

Figure S76. *^{13}C NMR spectra of **11**NO_3 in chloroform-d.*

References

1. M. A. Bennett and A. K. Smith, Arene ruthenium(II) complexes formed by dehydrogenation of cyclohexadienes with ruthenium(III) trichloride, *Journal of the Chemical Society, Dalton Transactions*, 1974, DOI: 10.1039/DT9740000233, 233-241.
2. A. B. Chaplin and P. J. Dyson, Catalytic Activity of Bis-phosphine Ruthenium(II)−Arene Compounds: Structure−Activity Correlations, *Organometallics*, 2007, **26**, 2447-2455.
3. D. N. Akbayeva, L. Gonsalvi, W. Oberhauser, M. Peruzzini, F. Vizza, P. Brüggeller, A. Romerosa, G. Sava and A. Bergamo, Synthesis, catalytic properties and biological activity of new water soluble ruthenium cyclopentadienyl PTA complexes [(C5R5)RuCl(PTA)2] (R = H, Me; PTA = 1,3,5-triaza-7-phosphaadamantane), *Chemical Communications*, 2003, DOI: 10.1039/B210102E, 264-265.
4. C. Scolaro, T. J. Geldbach, S. Rochat, A. Dorcier, C. Gossens, A. Bergamo, M. Cocchietto, I. Tavernelli, G. Sava, U. Rothlisberger and P. J. Dyson, Influence of Hydrogen-Bonding Substituents on the Cytotoxicity of RAPTA Compounds, *Organometallics*, 2006, **25**, 756-765.
5. C. Scolaro, A. Bergamo, L. Brescacin, R. Delfino, M. Cocchietto, G. Laurenczy, T. J. Geldbach, G. Sava and P. J. Dyson, In Vitro and in Vivo Evaluation of Ruthenium(II)−Arene PTA Complexes, *Journal of Medicinal Chemistry*, 2005, **48**, 4161-4171.
6. C. S. Allardyce, P. J. Dyson, D. J. Ellis, P. A. Salter and R. Scopelliti, Synthesis and characterisation of some water soluble ruthenium(II)–arene complexes and an investigation of their antibiotic and antiviral properties, *Journal of Organometallic Chemistry*, 2003, **668**, 35-42.
7. W. H. Ang, E. Daldini, C. Scolaro, R. Scopelliti, L. Juillerat-Jeannerat and P. J. Dyson, Development of Organometallic Ruthenium−Arene Anticancer Drugs That Resist Hydrolysis, *Inorganic Chemistry*, 2006, **45**, 9006-9013.
8. B. S. Murray, L. Menin, R. Scopelliti and P. J. Dyson, Conformational control of anticancer activity: the application of arene-linked dinuclear ruthenium(ii) organometallics, *Chemical Science*, 2014, **5**, 2536-2545.
9. J. R. Polam and L. C. Porter, Arene complexes of Ru(II) Part I. Synthesis, characterization and X-ray crystal structure of [(η6-C6H5CH3)RuCl(PPh3)2][BF4], *Inorganica Chimica Acta*, 1993, **205**, 119-121.
10. OECD, *Test No. 107: Partition Coefficient (n-octanol/water): Shake Flask Method*, 1995.
11. L. Hajji, C. Saraiba-Bello, A. Romerosa, G. Segovia-Torrente, M. Serrano-Ruiz, P. Bergamini and A. Canella, Water-Soluble Cp Ruthenium Complex Containing 1,3,5-Triaza-7-phosphaadamantane and 8-Thiotheophylline Derivatives: Synthesis, Characterization, and Antiproliferative Activity, *Inorganic Chemistry*, 2011, **50**, 873-882.
12. C. Fink, L. Chen and G. Laurenczy, Homogeneous Catalytic Formic Acid Dehydrogenation in Aqueous Solution using Ruthenium Arene Phosphine Catalysts, *Zeitschrift für anorganische und allgemeine Chemie*, 2018, **644**, 740-744.
13. L. Biancalana, E. Zanda, M. Hadiji, S. Zacchini, A. Pratesi, G. Pampaloni, P. J. Dyson and F. Marchetti, Role of the (pseudo)halido ligand in ruthenium(ii) p-

cymene α-amino acid complexes in speciation, protein reactivity and cytotoxicity, *Dalton Transactions*, 2021, **50**, 15760-15777.
14. A. Guerriero, W. Oberhauser, T. Riedel, M. Peruzzini, P. J. Dyson and L. Gonsalvi, New Class of Half-Sandwich Ruthenium(II) Arene Complexes Bearing the Water-Soluble CAP Ligand as an in Vitro Anticancer Agent, *Inorganic Chemistry*, 2017, **56**, 5514-5518.
15. B. S. Murray, S. Crot, S. Siankevich and P. J. Dyson, Potential of Cycloaddition Reactions To Generate Cytotoxic Metal Drugs In Vitro, *Inorganic Chemistry*, 2014, **53**, 9315-9321.
16. Y. Marcus, Thermodynamics of solvation of ions. Part 5.—Gibbs free energy of hydration at 298.15 K, *Journal of the Chemical Society, Faraday Transactions*, 1991, **87**, 2995-2999.
17. J. P. Tam, C. R. Wu, W. Liu and J. W. Zhang, Disulfide bond formation in peptides by dimethyl sulfoxide. Scope and applications, *Journal of the American Chemical Society*, 1991, **113**, 6657-6662.
18. D. Ortiz, N. Gasilova, F. Sepulveda, L. Patiny, P. J. Dyson and L. Menin, Aom2S: A new web-based application for DNA/RNA tandem mass spectrometry data interpretation, *Rapid Communications in Mass Spectrometry*, 2020, **34**, e8927.
19. W. Humphrey, A. Dalke and K. Schulten, VMD: Visual molecular dynamics, *Journal of Molecular Graphics*, 1996, **14**, 33-38.
